# Identification of a combined hypoxia and lactate metabolism prognostic signature in lung adenocarcinoma

**DOI:** 10.1186/s12890-024-03132-4

**Published:** 2024-07-04

**Authors:** Jingyang Sun, Rongxuan Jiang, Liren Hou, Lei Wang, Meng Li, Huanhuan Dong, Niuniu Dong, Yihan Lin, Zijiang Zhu, Guangjian Zhang, Yanpeng Zhang

**Affiliations:** 1 Department of Thoracic Surgery, The First Affiliated Hospital of Xi’an Jiaotong University, Xi’an, Shaanxi 710061 China; 2 Key Laboratory of Enhanced Recovery After Surgery of Integrated Chinese and Western Medicine, The First Affiliated Hospital of Xi’an Jiaotong University, Xi’an, Shaanxi 710061 China; 3 Biobank, The First Affiliated Hospital of Xi’an Jiaotong University, Xi’an, Shaanxi 710061 China; 4 Department of Respiratory and Critical Care Medicine, The First Affiliated Hospital of Xi’an Jiao Tong University, Xi’an, China; 5 Department of Thoracic Surgery, Gansu Province Central Hospital, Lanzhou, Gansu 730070 China

**Keywords:** LUAD, Hypoxia, Immunotherapy, Lactate metabolism, Lasso

## Abstract

**Background:**

In the tumor microenvironment (TME), a bidirectional relationship exists between hypoxia and lactate metabolism, with each component exerting a reciprocal influence on the other, forming an inextricable link. The aim of the present investigation was to develop a prognostic model by amalgamating genes associated with hypoxia and lactate metabolism. This model is intended to serve as a tool for predicting patient outcomes, including survival rates, the status of the immune microenvironment, and responsiveness to therapy in patients with lung adenocarcinoma (LUAD).

**Methods:**

Transcriptomic sequencing data and patient clinical information specific to LUAD were obtained from comprehensive repositories of The Cancer Genome Atlas (TCGA) and Gene Expression Omnibus (GEO). A compendium of genes implicated in hypoxia and lactate metabolism was assembled from an array of accessible datasets. Univariate and multivariate Cox regression analyses were employed. Additional investigative procedures, including tumor mutational load (TMB), microsatellite instability (MSI), functional enrichment assessments and the ESTIMATE, CIBERSORT, and TIDE algorithms, were used to evaluate drug sensitivity and predict the efficacy of immune-based therapies.

**Results:**

A novel prognostic signature comprising five lactate and hypoxia-related genes (LHRGs), PKFP, SLC2A1, BCAN, CDKN3, and ANLN, was established. This model demonstrated that LUAD patients with elevated LHRG-related risk scores exhibited significantly reduced survival rates. Both univariate and multivariate Cox analyses confirmed that the risk score was a robust prognostic indicator of overall survival. Immunophenotyping revealed increased infiltration of memory CD4 + T cells, dendritic cells and NK cells in patients classified within the high-risk category compared to their low-risk counterparts. Higher probability of mutations in lung adenocarcinoma driver genes in high-risk groups, and the MSI was associated with the risk-score. Functional enrichment analyses indicated a predominance of cell cycle-related pathways in the high-risk group, whereas metabolic pathways were more prevalent in the low-risk group. Moreover, drug sensitivity analyses revealed increased sensitivity to a variety of drugs in the high-risk group, especially inhibitors of the PI3K-AKT, EGFR, and ELK pathways.

**Conclusions:**

This prognostic model integrates lactate metabolism and hypoxia parameters, offering predictive insights regarding survival, immune cell infiltration and functionality, as well as therapeutic responsiveness in LUAD patients. This model may facilitate personalized treatment strategies, tailoring interventions to the unique molecular profile of each patient’s disease.

**Supplementary Information:**

The online version contains supplementary material available at 10.1186/s12890-024-03132-4.

## Introduction

Lung adenocarcinoma (LUAD) is a common malignant tumor originating from glandular epithelial cells of the lung [1]. It is often associated with various factors, such as smoking, environmental pollution, and genetic factors [2]. The pathogenesis of LUAD is complex and involves gene mutations, abnormal protein expression, and dysregulation of noncoding RNAs [3]. Despite some progress made by researchers worldwide in the treatment of lung cancer, the prognosis of LUAD patients remains unfavorable, with low survival rates. The development of molecular biology detection techniques, such as gene mutation detection and protein expression analysis, has provided new means for the diagnosis of LUAD. However, the complexity and heterogeneity of LUAD treatment still pose challenges. On the one hand, there are differences in the molecular mechanisms and treatment responses of LUAD patients, leading to variations in individual responses to treatment. On the other hand, early diagnosis and prognosis assessment of LUAD still present difficulties, and more accurate and reliable prognostic biomarkers are needed to guide clinical decisions.

Hypoxia is a common feature of all solid tumors [4, 5]. Several factors associated with cancer, such as uncontrollable tumor growth, dysfunctional microangiogenesis within the tumor, and tumor-induced anemia, can lead to hypoxia [6, 7]. Numerous studies have shown that hypoxia modulates tumor proliferation, angiogenesis, invasiveness, metastasis and resistance to radiotherapy [6]. Hypoxia can lead to immunosuppression by regulating angiogenesis, promoting immunosuppression, and enhancing tumor resistance to drugs. HIF-1, a member of the hypoxia-inducible factor (HIF) family, plays a crucial role in regulating the expression of various genes that contribute to the adaptation and advancement of tumor cells [8]. Numerous research studies indicate that increased levels of HIF-1 in different types of solid tumors, such as breast, colon, stomach, and lung cancers, are linked to lower survival rates. Additionally, HIF-1-driven changes in metabolism lead to the buildup of lactate within tumors [9].

In a hypoxic environment, tumor cells convert glucose to lactic acid via the lactic acid fermentation pathway rather than completely oxidizing it. This increase in lactate metabolism is known as lactic acidosis. Lactic acid, a byproduct of anaerobic glycolysis, has been linked to the development, advancement, tumor microenvironment, spread, and resistance to cancer treatment [10]. Similarly, increased levels of lactate metabolites in the tumor microenvironment create an immunosuppressive environment conducive to cancer cell proliferation and immune evasion. Lactate is not only a byproduct of glycolysis but also a key regulator of signal transduction pathways in normal and malignant tissues [11, 12].

Lactate produced as an end product of lactic acid fermentation following glycolysis is exported from cells via monocarboxylate transporter 4 (MCT4) [13]. Cancer cells use monocarboxylate transporter protein 1 (MCT1) [14] to uptake lactate and convert it to pyruvate via the enzymatic activity of lactate dehydrogenase-B (LDH-B) [15]. However, under hypoxic conditions, the hypoxia-inducible factor HIF-1 mediates the induction of the glycolytic phenotype by increasing glucose influx and enhancing lactate efflux through the monocarboxylate transporter subtype MCT4 [16]. The hypoxic environment elevates the expression levels of the monocarboxylate transporter MCT4 and hypoxia-inducible factor-1α (HIF-1α). Increased lactate efflux leads to increased lactate accumulation in the microenvironment, which in combination with HIF-1-induced metabolic reprogramming leads to a decrease in extracellular extracellular fluid pH through upregulation of glycolysis. Acidification of the TME exacerbates hypoxia, and hypoxia-induced high expression of MCTs contributes to the proliferation of cancer cells that are better able to maintain the appropriate pH to promote tumor growth [17]. Thus, hypoxia and lactate metabolism reinforce each other and together lead to a malignant tumor microenvironment [13].

Both hypoxia [18, 19] and altered lactate metabolism [20] in lung adenocarcinoma are important factors influencing the prognosis of patients receiving tumor therapy. Most of the previously reported prognostic models have analyzed hypoxia [21–23] and lactate metabolism [24, 25] individually, and the resulting prognostic models lack completeness. Hypoxia and lactate are not independent of each other and therefore deserve to be analyzed together. In the present study, by including both lactate metabolism related genes (LMRGs) and hypoxia related genes (HRGs) in LUAD, the aim was to analyze them in a comprehensive and systematic way to construct a valid prognostic model.

## Method and material

### Data collection and preprocessing

The TCGA-GDC (www.portal.gdc.cancer.gov) data portal contains 459 LUAD and 51 normal tissue samples. For the external validation group, the GSE31210 (*n* = 246), GSE42127 (*n* = 176), and GSE72094 (*n* = 442) datasets were downloaded from the GEO database (www.ncbi.nlm.nih.gov/geo). The baseline characteristics of the training and validation groups are shown in Supplementary Table 1. We acquired gene expression profiles and clinical information for our research. “HYPOXIA, LACTATE” was used to search the Molecular Signatures Database (https://www.gsea-msigdb.org/gsea/msigdb), and 200 hypoxia genes and 284 lactate metabolism genes were identified.

### Construction of the lactate metabolism and hypoxia signature in LUAD

The “Wilcox test” function of the R package “limma” was used, and the Mann‒Whitney test was used to determine DEGs. Next, we employed univariate Cox regression analysis to evaluate the predictive value of lactate metabolism and hypoxia-related genes (LHRGs) for overall survival in the training cohort. The predictive LHRGs and DEGs were then compared, and the common genes were considered to be predictive DEGs. Tumor samples from TCGA were used to create the training datasets for the prognostic signature. The LASSO regression adds an L1 regularisation term to the standard linear regression, which is the sum of the absolute values of the regression coefficients. LASSO regression compresses some regression coefficients to zero, which means that the corresponding features are removed from the model. This property makes LASSO suitable for high-dimensional data because it automates variable selection to provide a combinatorial model of the genes. The LASSO regression method was utilized to identify potential genes. The procedure identified the best penalty parameter λ for each gene to calculate the risk score coefficient. Ultimately, the variables were linearly combined to obtain the formula. The following formula was used to construct the model:

$$RiskScore = N \sum i=1 \left(exp*coef\right)$$

The gene number is represented by ‘N’, the gene expression is represented by ‘exp’, and the gene’s corresponding coefficient is represented by ‘coef’. Using this model, patients with TCGA-LUAD were divided into groups based on high or low risk. Survival analysis by Kaplan‒Meier analysis was used to compare OS between the two groups. The ROC curve was created with the ‘timeROC’ R package (v4.21) to evaluate the sensitivity and specificity of the gene signature. The R software packages ‘stats’ and ‘Rtsne’ were utilized to reveal inherent characteristics in these two groups through principal component analysis (PCA) and t-distributed stochastic neighbor embedding (t-SNE). The TCGA cohort was analyzed using univariate and multivariate Cox regression to assess the risk score, taking into account sex, age, stage, and risk score. By applying the same approach utilized in the TCGA lasso analysis, we calculated a risk score for every individual, subsequently dividing patients from the GEO database into high- and low-risk categories.

### Predictive validation of the prognostic model

The effectiveness of this model was validated by the C-index, independent Cox prognosis analysis, age, sex, stage, and risk score. A nomogram was created to predict the survival of LUAD patients at 1, 3, and 5 years by incorporating the risk score and additional clinical data.

### Functional enrichment analysis

GSEA (www.gsea-msigdb.org/), Gene Ontology (GO) and Kyoto Encyclopedia of Genes and Genomes (KEGG) pathway analyses were performed using the “org.hs.eg.db” (version 3.10.0), “clusterprofiler” package (version 3.14.3), and “ggplot2” package (version 3.3.3) in R software. The analysis was based on Homo sapiens, and screening criteria of p.adjust < 0.1 and p value < 0.2 were adopted to identify the primary enriched functions and pathways.

### Differences in the tumor immune microenvironment patterns between risk groups

A sophisticated algorithm, CIBERSORT, employs a series of reference transcriptome information to estimate the proportion of 22 distinct immune cells found in bulk tumor sample expression data. This estimation is carried out using linear support vector regression principles. To evaluate immune-related functions, we utilized ssGSEA with the aid of the “GSVA” R package. The ESEIMATE algorithm was used to analyze immune cell and stromal cell infiltration in the tumor microenvironment (TME).

### Tumor mutational load and microsatellite instability

To examine the differences in somatic mutations between the low and high-risk groups, somatic mutations from TCGA were analysed by using the R package ‘maftools’ and the tumor mutation burden (TMB) of each patient was assessed between the two groups. In the analysis of TMB and microsatellite instability (MSI), the correlation between gene expression and TMB and MSI scores was calculated using Spearman correlation analysis, and p < 0.05 was considered statistically significant.

### Evaluation of drug sensitivity and efficacy of immunotherapy

The ‘oncoPredict’ R package was utilized to predict the response of various sample groups to chemotherapy by calculating the 50% maximum inhibitory concentration (IC50) through ridge regression. We used the Wilcoxon signed-rank test to analyze the IC50 values of the different groups. Additionally, the TIDE algorithm, available at http://tide.dfci.harvard.edu/, utilizes transcriptome data to evaluate the TME. Published research has confirmed its efficacy in predicting the outcome of immune checkpoint blockade (ICB) treatment. A p value less than 0.05 was considered to indicate statistical significance.

### Statistical analysis

R language (version 4.2.1) was utilized for bioinformatics analysis, excluding descriptive analysis. We corrected the p values for multiple testing by applying the t test and the Benjamini–Hochberg method, which accounts for the false discovery rate (FDR). A P value less than 0.05 was considered to indicate statistical significance.

## Results

**Fig. 1:**
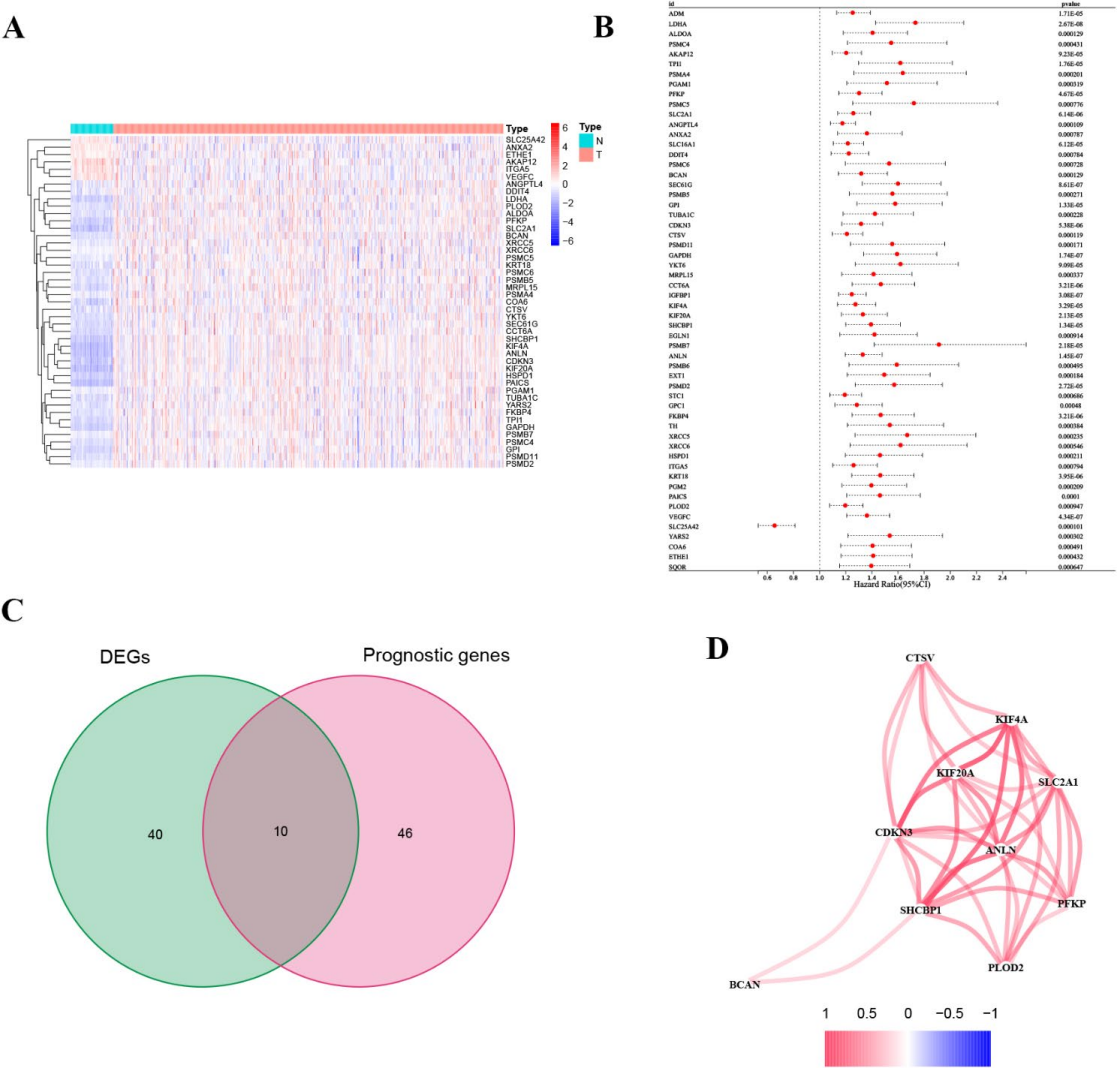
The expression and interaction of overlapping LH-related genes. (**A**) The expression profiles of the 50 differentially expressed genes. (**B**) The 56 prognostic LH-related genes in LUAD. (**C**) Venn diagram. (**C**) Expression of the 4 overlapping genes in LUAD. (**D**) Correlation network between 10 intersecting genes; *p* < 0.05 was considered to indicate statistical significance

We identified 50 genes that were significantly differentially expressed between 541 tumor and 59 normal tissues (*p* < 0.001, and logFC | > 2), as illustrated in the heatmap (Fig. 1A). A total of 504 patients were selected for the TCGA training set to identify genes related to prognosis after 96 patients with inadequate follow-up or insufficient clinical information were removed. Fifty-six prognostic genes were selected based on OS in the TCGA cohort using univariate Cox regression analysis. Next, we utilized a Venn diagram to discover the 10 predictive DEGs (Fig. 1C). The Pearson correlation coefficient was calculated for the 10 prognostic DEGs to assess potential interactions among these genes with a threshold of 0.2 (Fig. 1D).

### Construction of the prognostic signature

**Fig. 2:**
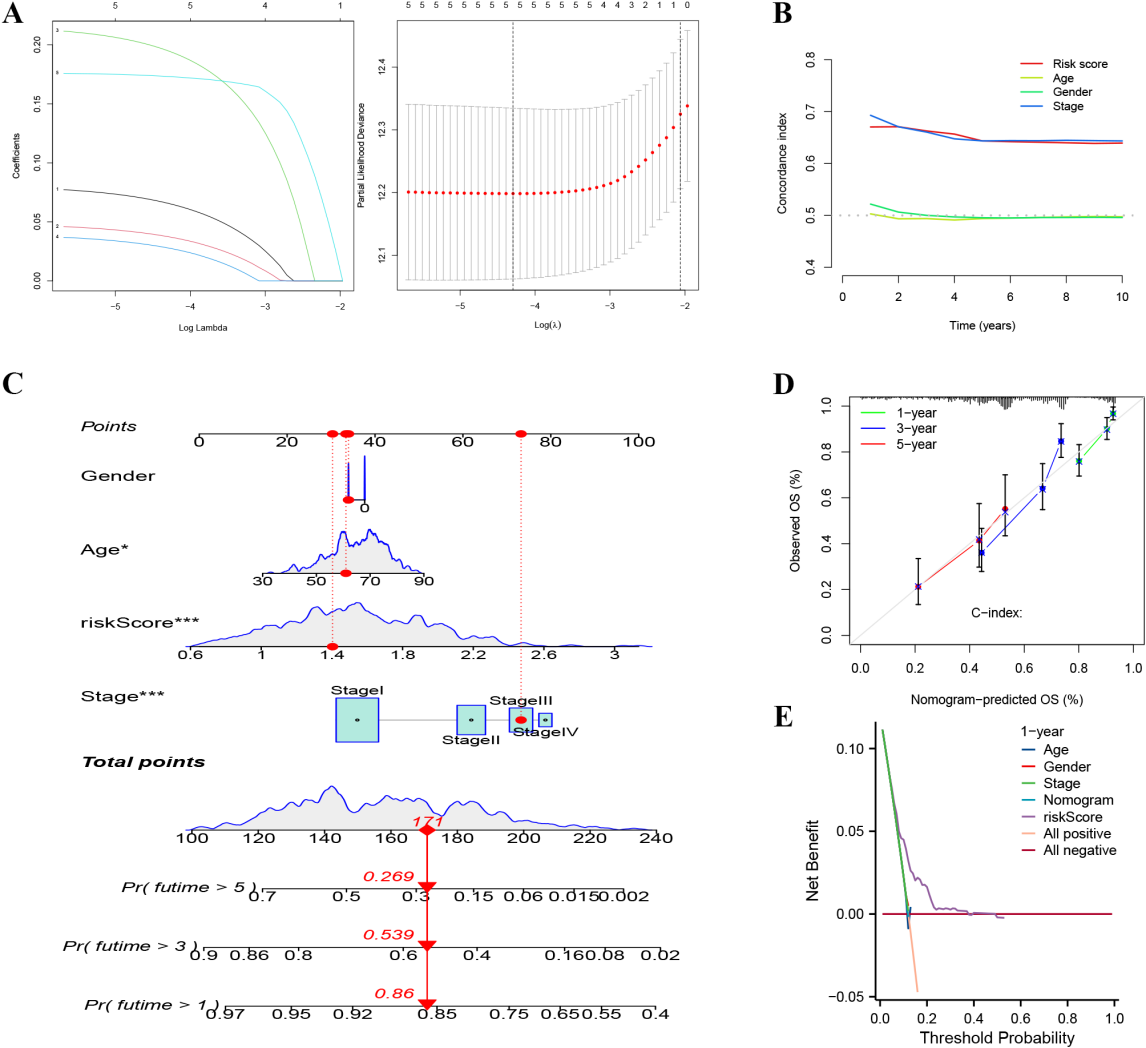
Construction of a five-gene HL signature in the training cohort. (**A**) Association between the coefficients of genes and log(lamba), deviance and log(lamba). (**B**) C-index curve analysis of risk-model. (**C**) The nomogram based on the risk-related prognostic signature and clinicopathological factors, including sex, age, and stage. (**D**) Calibration curves for the nomogram for predicting 1-, 3-, and 5-year survival in LUAD patients from the TCGA. (**E**) DCA curve of risk-model

The LASSO algorithm is a prognostic signature for LUAD patients based on five LHRGs (Fig. 2A). The risk score for this distinctive signature was calculated as follows: risk score = (0.0607×exp (PFKP)) + (0.0338×exp (SLC2A1)) + (0.1873×exp (BCAN)) +(0.0240×exp (CDKN3)) + (0.1719×exp (ANLN)). Patients were divided into two distinct groups according to the median risk score obtained from the training cohort: high-risk (*N* = 251) and low-risk (*N* = 252). The c-index analysis indicated that the risk scores from the prognostic model had strong discriminatory ability and high accuracy when considering various clinical information (Fig. 2B). The nomogram incorporates the risk score, stage, age, and sex and predicts the 1-, 3-, and 5-year OS of patients with LUAD (Fig. 2C). The calibration curves (Fig. 2D) demonstrated the concurrence in the observation and prediction of 1-, 3-, and 5-year survival in the training groups, indicating the reliability of the nomogram for predicting the survival outcomes of patients with LUAD at these time points. Decision curve analysis (DCA) suggested that the risk score had better predictive performance (Fig. 2E).

**Fig. 3:**
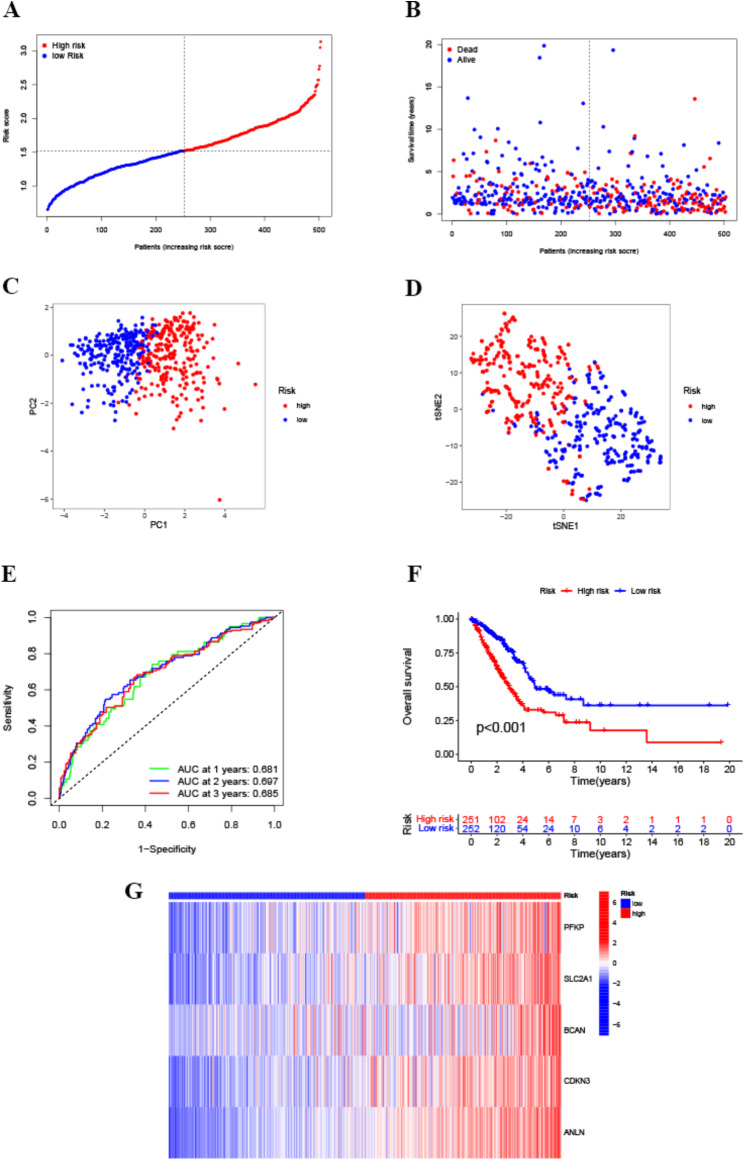
TCGA cohort prognostic signature construction. Distribution of risk scores according to the (**A**) OS and (**B**) survival status of TCGA-LUAD patients. (**C**) PCA and (**D**) t-SNE analysis of the training group. (**E**) ROC curve analysis and (**F**) Kaplan‒Meier survival analysis of patients stratified according to the five LH-related gene signatures. (**G**) Heatmap showing the expression profiles of the five LH-related genes in the high/low-risk groups

The samples in the training group were divided into high-risk and low-risk subgroups using the risk score calculated with the formula mentioned above (Fig. 3A), which revealed that the high-risk group had a greater likelihood of experiencing earlier mortality (Fig. 3B). PCA, t-SNE, and ROC curve analyses were performed to evaluate the predictive efficacy of the signature. As shown in Fig. 3C and D, both PCA and t-SNE analyses revealed that the prognostic signature effectively separated patients into high-/low-risk groups. The ROC curve was subsequently used to assess the prognostic significance of the marker for overall survival in individuals diagnosed with LUAD, with AUC values of 0.681, 0.697, and 0.685 at 1, 2, and 3 years, respectively (Fig. 3E). The Kaplan‒Meier plot indicated that individuals in the high-risk category had a notably reduced overall survival compared to those in the low-risk category (Fig. 3F, *p* < 0.01). In Fig. 3G, the expression levels of the five genes that were differentially regulated are displayed for each sample in the two risk groups. CDKN3, ANLN, SLC2A1 were differentially expressed in tumor tissues and normal tissues of esophageal cancer (ESCA), gastric carcinoma (STAD), clear cell carcinoma of the kidney (KIRC), squamous lung cancer (LUSC), hepatocellular carcinoma (LIHC), adenocarcinoma of the rectum (READ), cholangiocarcinoma (CHOL), and were expressed higher in tumor tissues than in normal tissues. PFKP was differentially expressed in other tumor types except for READ and was expressed higher in tumor tissues than in normal tissues. BCAN, although differentially expressed in multiple tumor types was also differentially expressed and had overall lower expression in the solid tumors mentioned above. These 5 genes are also highly expressed in LUSC (Supplementary Fig. 1).

### Validation of the lactate and hypoxia-related signature in the GEO cohort

**Fig. 4:**
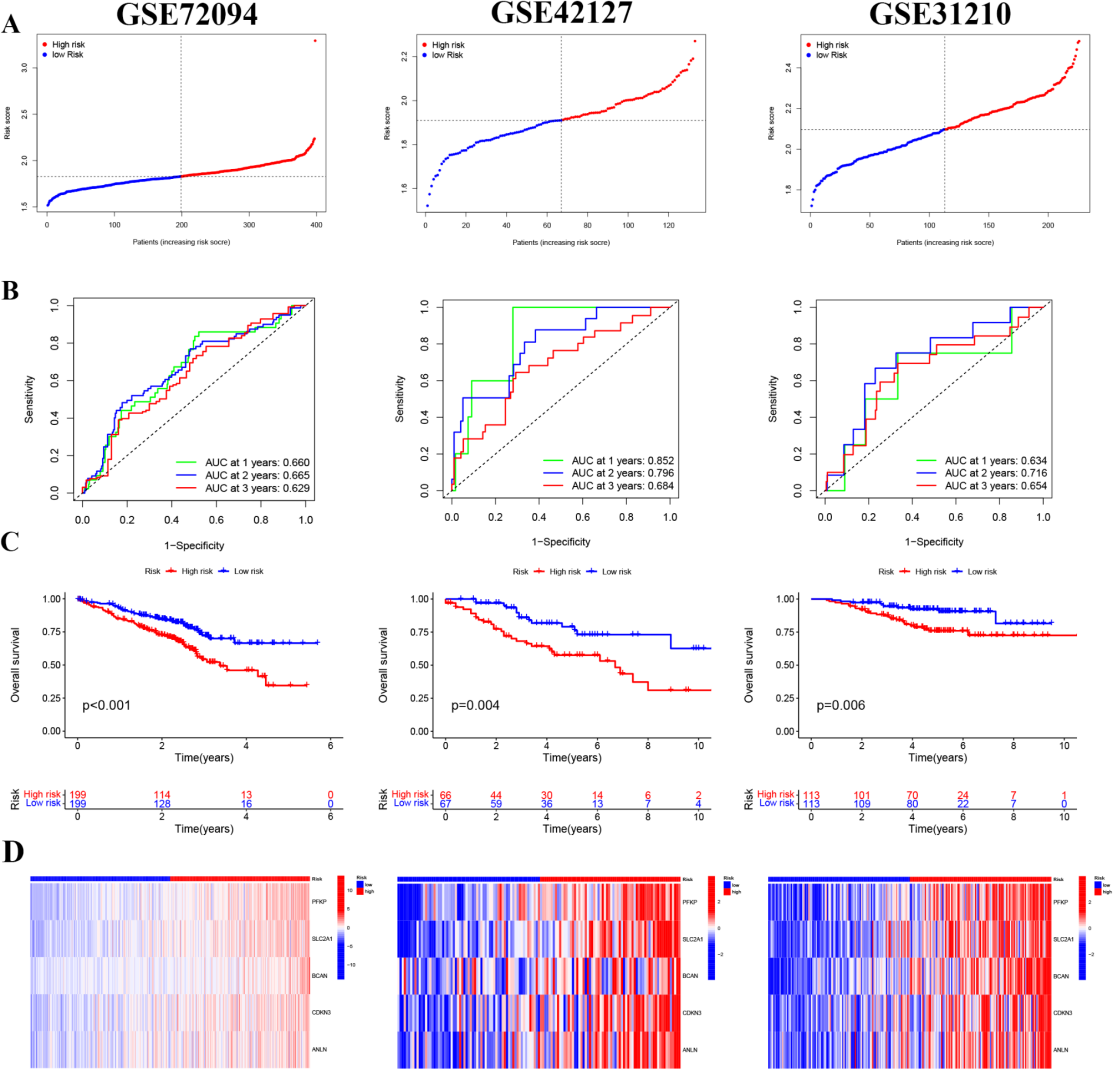
Validation of the five-gene signature in external cohorts: GSE72094 (left), GSE42127 (middle), and GSE31210 (right). (**A**) The risk score distribution of the three validation groups. (**B**) Time-dependent ROC curve analysis of the five-gene signature for predicting overall survival. (**C**) Kaplan‒Meier survival analysis of the high- and low-risk groups. (**D**) The expression profiles of the five HL genes in the validation cohort

The five-gene model established in the training group was applied to the GSE31210, GSE42127 and GSE72094 cohorts. Patients in the three GEO validation cohorts were divided into high- and low-risk groups (Fig. 4A). The AUC of GSE31210 reached 0.634, 0.716, and 0.654 at 1, 2, and 3 years, respectively; the AUCs of GSE42127 were 0.852, 0.796, and 0.684, respectively; and the AUCs of GSE72094 were 0.660, 0.665, and 0.629, respectively (Fig. 4B). K‒M curve analysis of the validation cohort demonstrated that patients in the high-risk group had shorter OS than did those in the low-risk group (Fig. 4C). The heatmap showed that the expression trends of the five-gene signature between the high- and low-risk samples in the GEO validation group were similar to those in the training group (Fig. 4D).

### Independent prognostic analysis

**Fig. 5:**
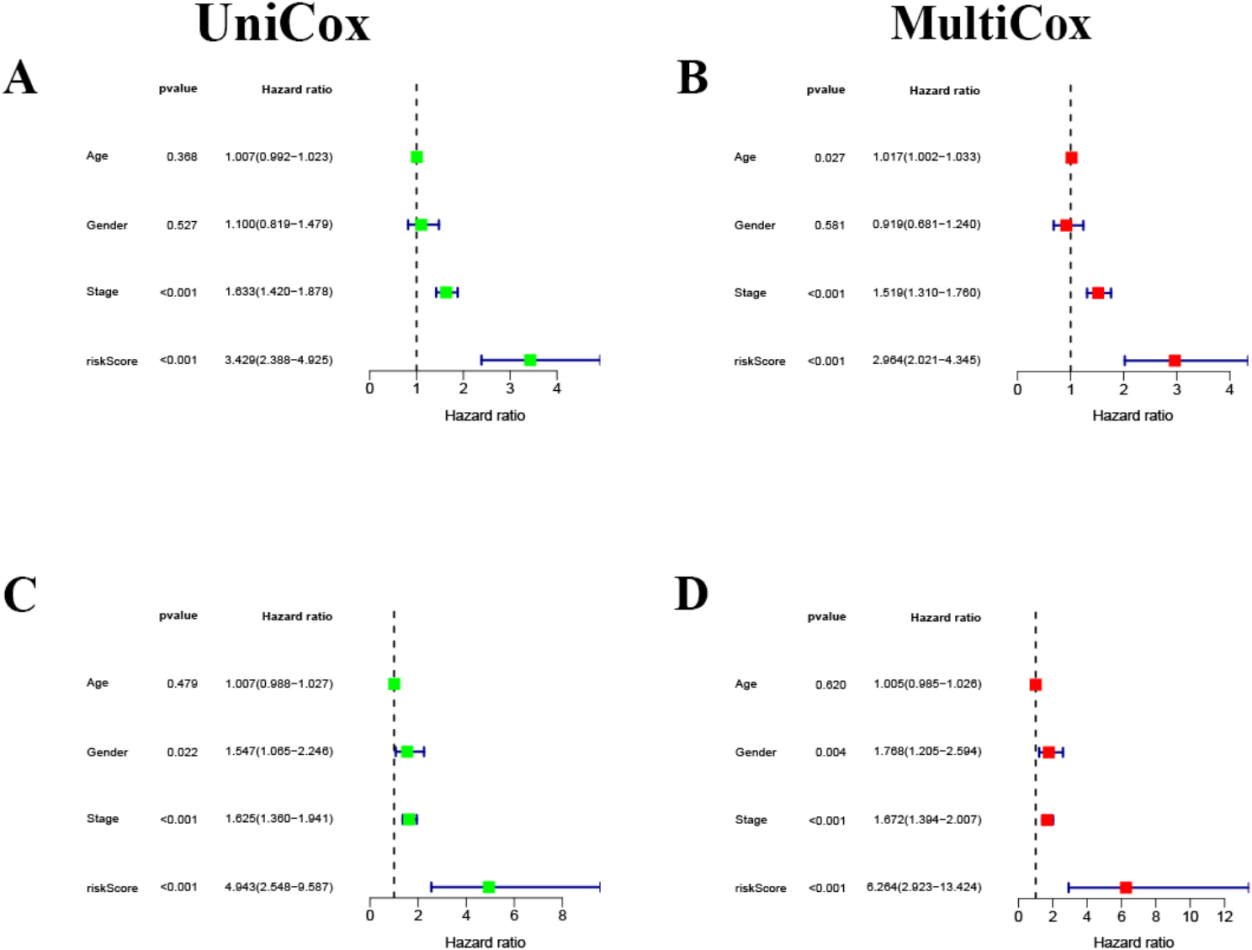
Independent prognostic analysis. (**A**) TCGA cohort univariate Cox regression. (**B**) TCGA cohort multivariate Cox regression. (**C**) Univariate Cox regression of the GSE72094 cohort. (**D**) Multivariate Cox regression of the GSE72094 cohort

Univariate Cox regression analysis of the training cohort revealed that stage, age, sex, and risk score were clinically significant (Fig. 5A). However, multivariate analysis revealed that only stage and risk score were significant prognostic factors (Fig. 5B). Age, sex, tumor stage, and risk score were included in the GEO validation cohort GSE72094. Multivariate and univariate Cox regression analyses demonstrated that the stage and risk score were significantly different (Fig. 5C, D). Stage, age, and gender for survival analysis (Supplementary Fig 4) results could be viewed in the Supplementary Materials.

### Functional enrichment analysis

**Fig. 6:**
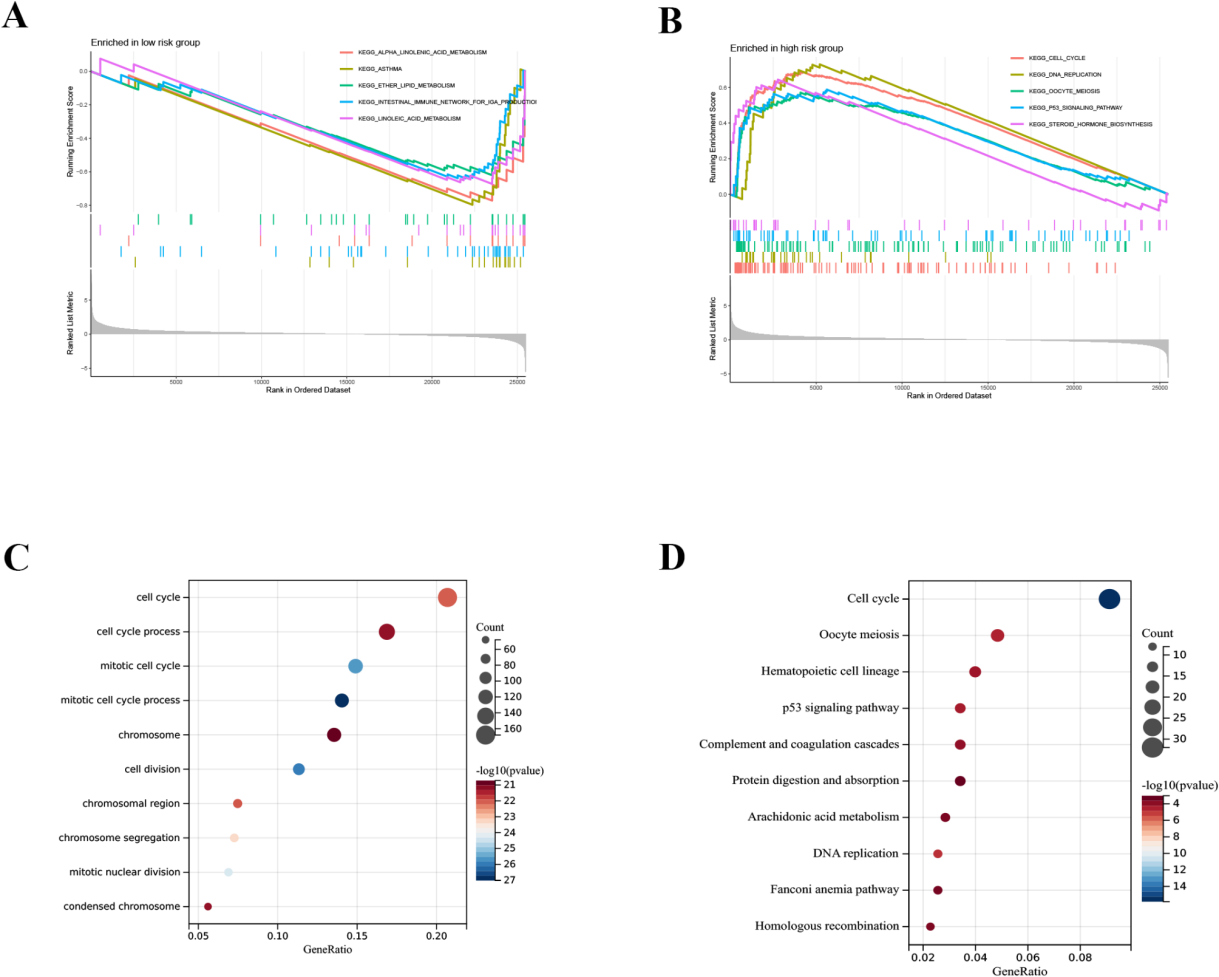
Functional enrichment analysis. Differential GSVA functional enrichment between low- (**A**) and high-risk (**B**) LUAD patients. The GO (**C**) and KEGG (**D**) pathways of the risk score-related genes

We utilized GSEA software to identify the KEGG enrichment pathways to investigate the relationships between the two risk groups. In the high-risk group, there were 29 pathways enriched with NESs greater than 1.5, NOM p values less than 0.05, and FDRs less than 0.25, while in the low-risk group, there were 89 pathways enriched with the same criteria. Five different pathways were identified in the high- and low-risk groups: the cell cycle, linoleic acid metabolism, asthma, the citrate cycle, the TCA cycle, ubiquitin-mediated proteolysis, and the p53 signaling pathway (Fig. 6A, B). In the training group, a total of 1187 genes that were differentially expressed and related to risk were identified (Supplementary Table 2). We conducted analyses on the DEGs associated with risk to investigate the potential functions or pathways linked to risk scores using GO and KEGG methods. The pathways involved in tumor cell proliferation included the cell cycle, DNA replication, and pyrimidine metabolism (Fig. 6C, D).

### Immune analysis

**Fig. 7:**
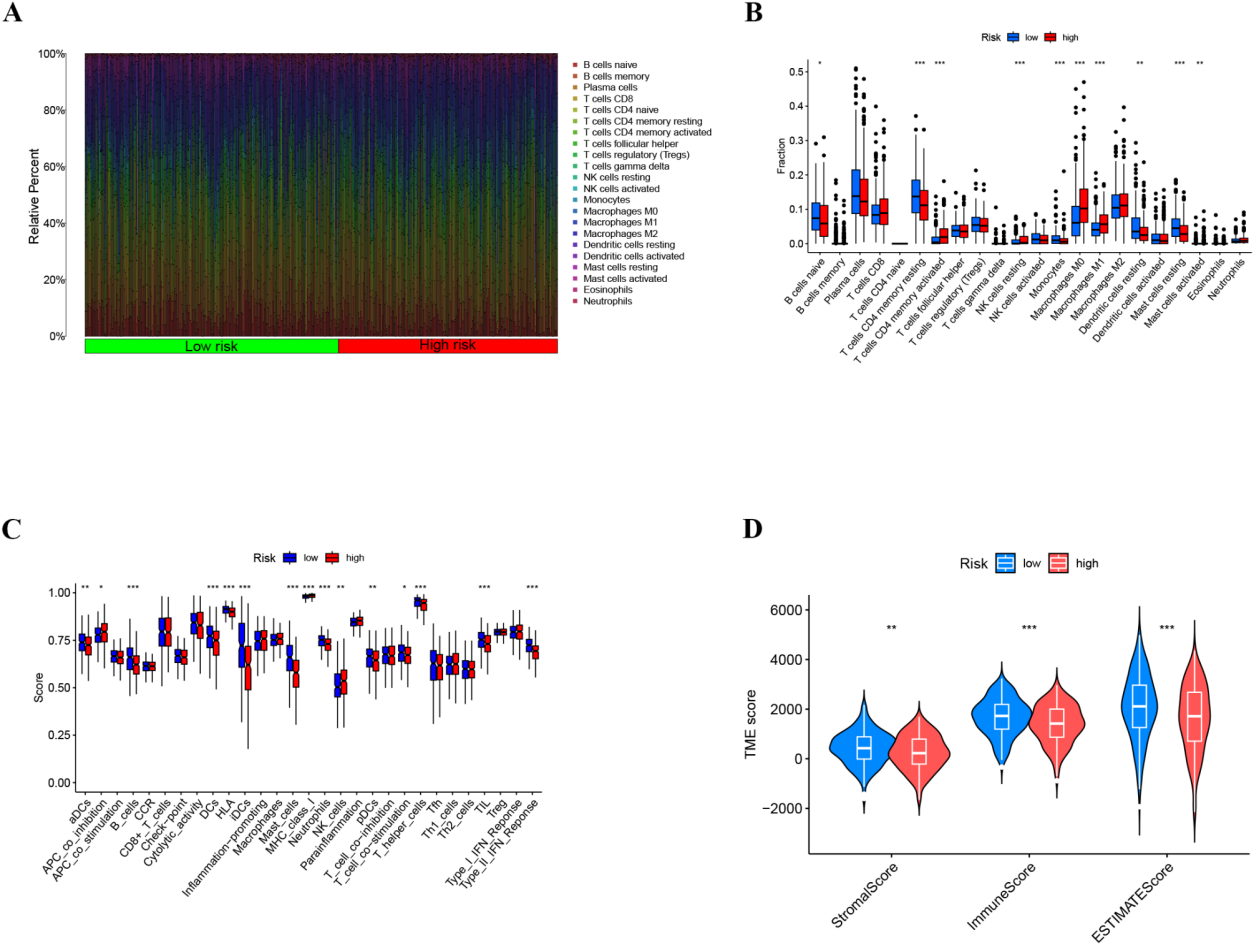
Different immune cell infiltration levels in high- and low-risk LUAD patients. (**A**) A stacking diagram was generated to illustrate the immune infiltration of immune cells in TCGA-LUAD patients using the CIBERSORT algorithm. (**B**) Immune function analysis based on ssGSEA. (**C**) Box plot showing that immune infiltration levels differed between the high- and low-risk groups. (**D**) Violin plots showing the differences in stromal and immune scores between the high-risk and low-risk groups

To further elucidate the underlying association between the risk score and immune response, immune cell infiltration was quantified by CIBERSORT analysis using gene expression data. There was an increase in T cells and a smaller proportion of B cells in morphologically diverse samples (Fig. 7A). Identification and assessment of differences in immune cell infiltration between high- and low-risk LUAD samples by the CIBERSORT algorithm revealed that CD4 + T-cell memory (resting and activated), resting NK cells, monocytes, M0/M1 macrophages, and mast cells were significantly differentially expressed between risk groups (Fig. 7B). According to these differential immune cell analyses, the numbers of resting CD4 T cells, mast cells and dendritic cells were reduced in the high-risk group, while the numbers of activated memory CD4 T cells and M0 macrophages were reduced in the low-risk group. In terms of immune function, B cells, DCs, iDCs, master cells, neutrophils, T helper cells, TILs, type II IFN responses and HLA levels were significantly different between the risk groups (Fig. 7C). Among these immune functions, only iDCs, B cells and mast cells were markedly suppressed in the high-risk group. In the low-risk group, the ESTIMATE, immune, and stromal scores were all greater than those in the high-risk group, according to violin plots (Fig. 7D). The impact of immune cell infiltration and immune function on survival (Supplementary Fig 2, 3) is shown in the Supplementary Material.

### TMB and MSI analysis

**Fig. 8:**
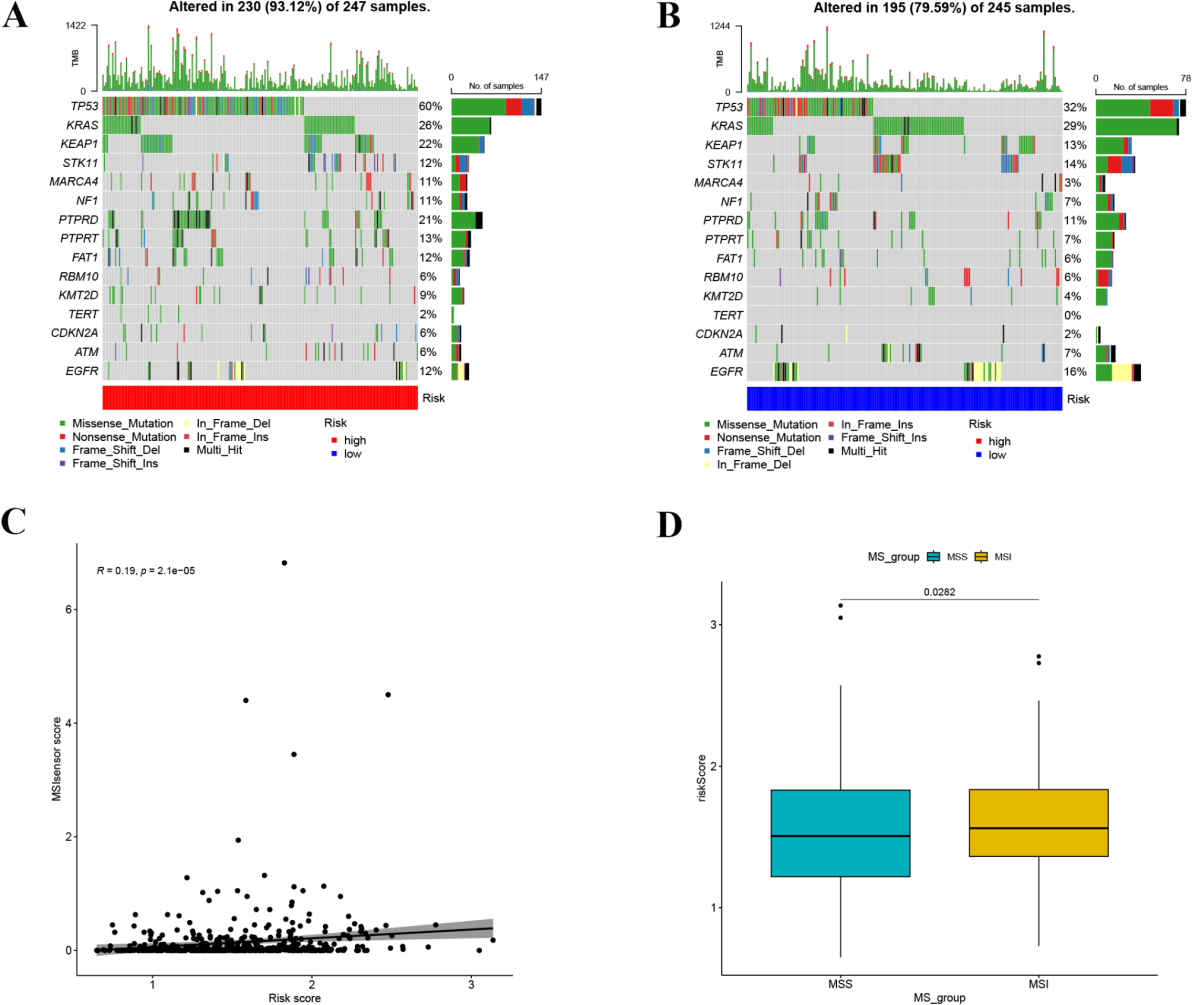
Genomic mutation and MSI analysis for LH signature. (**A**) Gene mutation frequency in high-risk group. (**B**) Gene mutation frequency in low-risk group. (**C**) Correlation of MSI with risk score. (**D**) Comparison of risk scores among MSS and MSI subpopulation

In the TCGA training cohort, we calculated the TMB of key driver genes for lung adenocarcinoma in each patient. Missense mutations were the most common type of somatic mutation. Overall, samples in the high-risk group had a higher probability of mutations compared to the low-risk group (Fig. 8A, B). Specifically, in high-risk group, the mutation probability as below: TP53 (60%), KRAS (26%), KEAP1 (22%), PTPRD (21%); in low-risk group, TP53 (32%), KRAS (29%), KEAP1 (13%), STK11 (14%), EGFR (16%). Moreover, the mutation frequency differences between the two groups were significant, with TP53, KEAP1, and PTPRD being the most notable (Fig. 8A, B). TP53 had a mutation probability of 60% in the high-risk group, compared to only 32% in the low-risk group, showing a substantial difference in mutation probability. MSI was positively correlated with the risk scores of this prognostic model (Fig. 8C, *p* = 2.1e-05). Micro Satellite stability (MSS) differed from MSI in both groups of samples (*p* = 0.0282, Fig. 8D), with the median risk score being higher in the MSI group. It proves that the risk score associated with prognostic signature is positively correlated with MSI in LUAD samples. There was no significant correlation between MSI and the five genes constituting the prognostic model (Supplementary Fig. 5). To further explore the relationships between each gene’s expression level with lung adenocarcinoma driver gene’s mutation frequence, we displayed the top 35 diver genes with the highest correlation in expression and mutation probability, showing differences in LUAD samples. Among these, mutation sites in ANLN, PFKP, CDKN3, and SLC2A1 are widely encompassed within the key driver genes of LUAD, while mutation sites associated with BCAN expression are not as numerous but still present among the major driver genes of LUAD (Supplementary Fig. 6).

### The signature predicts the response to chemotherapy and immunotherapy

**Fig. 9:**
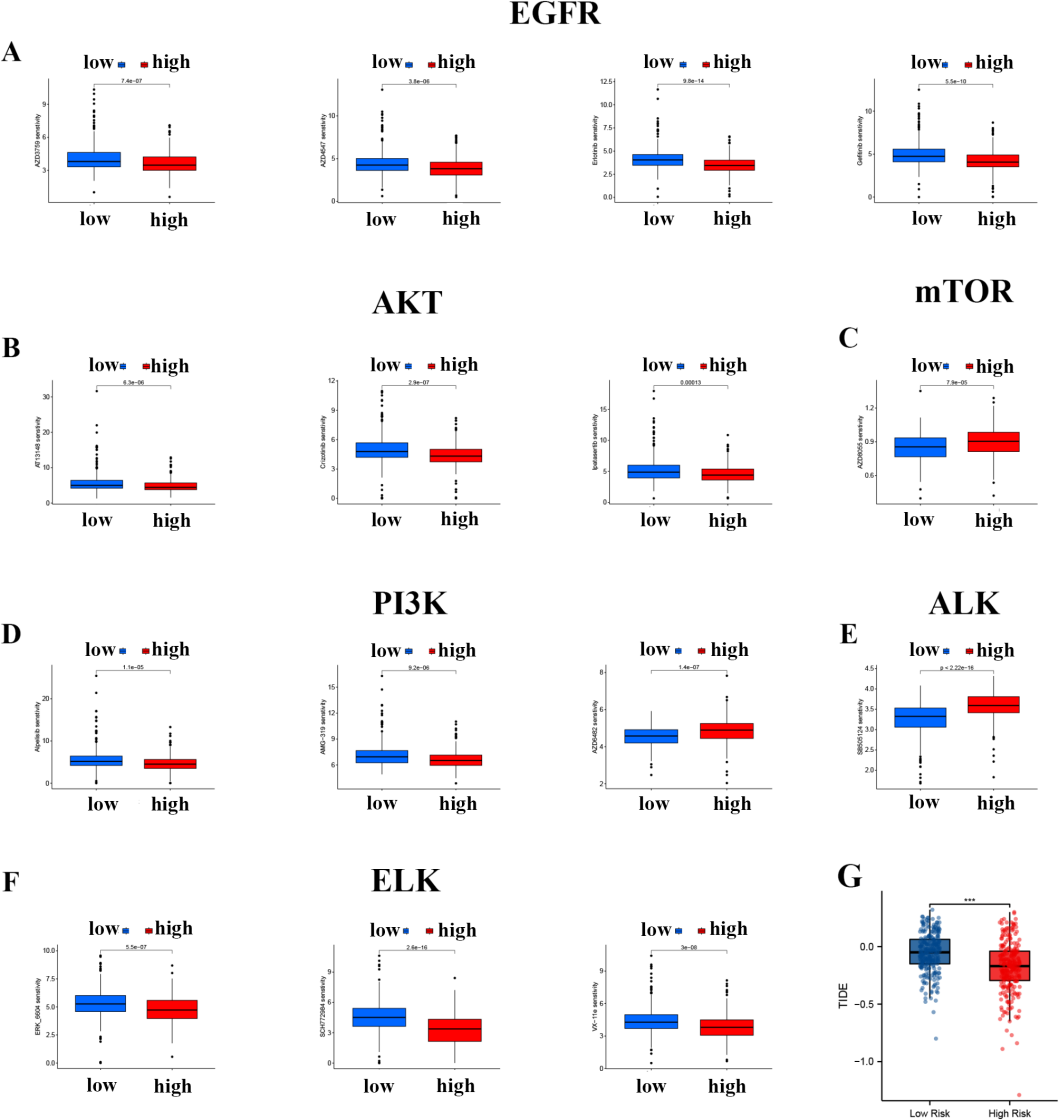
Prediction ability of the chemotherapy signature and immunotherapy. High-risk scores and lower IC50s for chemotherapeutics. (**A**) EGFR inhibitors, (**B**) AKT inhibitors, (**C**) mTOR inhibitors, (**D**) PI3K inhibitors, (**E**) ALK inhibitors, (**F**) ELK inhibitors. (**G**) Comparison of TIDE scores between the high- and low-risk groups

We analyzed the correlation between our model and drug therapy for LUAD by comparing the efficacy of drugs in high-risk and low-risk groups. We found a notable correlation between the low-risk category and increased IC50 values in patients exposed to various chemotherapy medications, including EGFR inhibitors, RAS/MAPK pathway inhibitors, mTOR inhibitors, and ELK pathway inhibitors. As shown in Fig. 9A, EGFR inhibitors showed increased effectiveness in the high-risk category when exposed to AZD3759, AZD4547, erlotinib, and gefitinib. With the exception of AZD6242, ATK and PI3K inhibitors, including AZD13148, crizotinib, ipatasertib, alpelisib, and AMG-319, showed increased sensitivity in the high-risk group (Fig. 9B, D). Similarly, the levels of the ELK-related drugs ERK_6604, SCH772984, and VX-11e were increased at increased risk (Fig. 9E). Conversely, drugs targeting mTOR and ALK were more effective in the low-risk group than in the other groups (Fig. 9C, E). Immunotherapy tolerance was evaluated in various risk categories using TIDE scoring. The results indicated a significant difference in TIDE scores between the high-risk and low-risk groups, with the high-risk group showing notably lower scores. However, the relationship between this prognostic model and anti-tumor drug sensitivity, related targets of action and signal pathways is also a concern of this study. Further study of the relationship between anti-tumor drug sensitivity and its associated pathways. We formed a correlation network (Supplementary Figs. 7–9 A) between the three pathways and the five genes of this prognostic signature, and a mutation analysis of the model and the genes related to the three pathways, respectively. In LUAD samples, the main mutation sites in the EGFR related RTK pathway are EGFR, ERBB4, PDGFRA, and KDR (Supplementary Fig. 7B, C). In the PI3K-AKT-mTOR pathway, mutations are found in 26.72% of high-risk group samples and 17.55% of low-risk group samples. PIK3CA and MTOR are the main mutated genes in two risk groups (Supplementary Fig. 8B, C). For the ELK related Ras-Raf-Mek-Erk pathway, mutations are present in 26.72% of high-risk group samples, compared to 17.55% in the low-risk group. KRAS and BRAF are the main mutation sites in both high and low-risk groups (Supplementary Fig. 9B, C). The results indicate that individuals classified as low risk have increased resilience to ICB and are more likely to evade the immune system; consequently, those in the high-risk category are expected to have a more positive reaction to ICB therapy.

## Discussion

Hypoxia and lactate metabolism both play a role in the tumor microenvironment. The research value and importance of LUAD, as the type of tumor with the highest mortality rate worldwide, is indisputable. In this study, we analyzed its potential effects on LUAD via the LASSO/Cox algorithm via bioinformatics and identified prognostically relevant biomarkers.

LASSO regression compresses some regression coefficients to zero, which means that the corresponding features are removed from the model. This feature makes LASSO suitable for high-dimensional data, providing an accurate, prognostic predictive model composed of genes by automating variable selection. According to the prognostic model constructed from LHRGs, the TCGA-LUAD samples and GEO validation cohorts were classified into high- and low-risk groups, which proved that the present model had good discriminatory ability. The predictive ability of this model for patient prognosis was confirmed by multiple GEO datasets, multiple testing algorithms for model efficacy, bioinformatics analysis and validation from multiple perspectives. It was confirmed that the model can independently and stably predict the prognosis of LUAD patients with good predictive efficacy and can also be considered an independent prognostic factor. Enrichment analyses around the model demonstrated that model-associated differential genes are widely enriched in the cell cycle. Our findings seem to coincide with the findings of studies showing that large accumulations of lactate remodel the late-promoting complex (APC/C) to regulate the cell cycle and proliferation through direct inhibition of the SUMO protease SENP1 [26].

As important features of the TME, both hypoxia and lactate modulate antitumor immune responses [27, 28]. Hypoxia attenuates antitumor immunity by increasing the protumorigenic M2 phenotype, intratumoral accumulation of immunosuppressive regulatory T cells and stimulation of adenosine receptors [29]. Lactate inhibits CD8 + and CD4 + effector T-cell function but increases helper T-cell 1 differentiation and interferon gamma (IFNγ) production. Hypoxia also upregulates PD-L1 by binding HIF-1 to a hypoxia-responsive element in the proximal promoter of PD-L1. Recent studies have reported that lactate specifically regulates programmed cell death protein 1 (PD-1) in effector regulatory T (eTreg) cells, leading to therapeutic failure of ICIs, and that inhibition of lactate metabolism in Treg cells enhances susceptibility to ICIs in drug-resistant tumors. sensitivity to ICIs [30]. Immune cell infiltration in TCGA-LUAD RNA-seq data was analyzed, and the CIBERSORT algorithm was used for samples from the high- and low-risk groups in this model. The percentages of memory CD4 + T cells, NK cells, resting dendritic cells and resting mast cells were significantly greater in the low-risk group than in the high-risk group, whereas the corresponding survival analyses revealed that greater infiltration resulted in poorer survival outcomes. Memory CD4 + T cells induce inflammatory death to control tumor immune escape [31], which echoes the results of the present study showing that activated memory CD4 + T-cell infiltration is greater in the high-risk group, possibly through tumor immune escape, to avoid tumor cell death and thus influence prognosis. The biomarkers screened in this model may suppress immune cells and immune function, which may have an impact on the survival of LUAD patients.

Based on the results of enrichment analysis in this study, risk genes in the high-risk group were enriched in the cell cycle, DNA replication, and p53 pathways. In the low-risk group, there was enrichment in lipid metabolism-related pathways. The cell cycle has been identified as a crucial player in tumor development. Our enrichment analysis revealed a significant association between cell cycle-related genes and lactate metabolism and hypoxia in lung adenocarcinoma. Recent research has demonstrated that lactate regulates the cell cycle by remodeling the anaphase-promoting complex [26]. Moreover, targeting the lactate-associated cell cycle could contribute to cancer therapy [32]. p53 functions as a transcription factor that activates or represses the transcription of multiple downstream target genes. The roles of these target genes mainly include induction of cell cycle arrest, DNA repair, regulation of cell metabolism, cellular senescence and apoptosis. In most cases, p53 inhibits glycolysis. First, p53 can inhibit glucose uptake by suppressing the expression or activity of the glucose transporter proteins GLUT1/3/4/12. Second, p53 inhibits several enzymes that regulate the glycolytic process, such as HK1/2, PFK1 and PGM. p53 also inhibits the transport of lactate out of the cell. In turn, accumulated lactate inhibits glycolysis [33]. Given the reliance of tumor cells on glycolysis and the Warburg effect for their growth and development, inhibiting glycolysis with p53 can hinder the proliferation of cancer cells.

Risk scores are also used as biomarkers for precision oncology to guide targeted therapy. Based on the Oncology Drug Sensitivity Database, the sensitivity correlations of several antineoplastic drugs with this risk model were determined. EGFR-targeted drugs target tyrosine kinase receptors that are frequently overactivated in lung adenocarcinoma cells due to genetic mutations, promoting tumor growth and survival. They can be divided into two broad categories: small molecule tyrosine kinase inhibitors (TKIs) and anti-EGFR antibodies. Erlotinib and gefitinib are first-generation small molecule TKI drugs. Both TKIs reversibly bind EGFR and are used for the treatment of locally advanced or metastatic non-small cell lung cancer. The PI3K-AKT-mTOR pathway is an intracellular signaling pathway that responds to extracellular signals to promote metabolism, proliferation, cell survival, growth and angiogenesis. The activated PI3K/AKT axis regulates GSK-3 β-kinase activity and FOXO1 transcription to increase GCK activity and decrease G6 Pase expression, thereby promoting hepatic glycogen synthesis and gluconeogenesis [34, 35]. Related drugs: Crizotinib [36], the only drug approved for targeting both ALK and ROS1 at home and abroad, has significantly better inhibitory effects against both mutations than traditional chemotherapy and is safer and more tolerable. Again, sensitivity was greater in the high-risk group, and such drugs may have better therapeutic effects against high-risk or even advanced NSCLC. Both pathway correlation network and mutation analyses demonstrated interactions between the present prognostic model and the three pathways mentioned above. Correlation network analyses suggest that interactions between the five genes associated with the model and the three pathways may affect the pathways through expression associations between genes leading to differences in drug sensitivity. Mutation analyses of the three pathway-associated genes indicated that differences in drug sensitivity between the high- and low-risk groups may result through differences in mutations in specific targets in the pathways. Both analytical perspectives demonstrate the interaction between the prognostic model and the three pathways described above, as well as side-by-side explanations for the differences in drug sensitivity associated with the present prognostic model.

MSI is an important phenomenon in tumor development. Different microsatellite loci have varying stabilities. Factors such as the length of the microsatellite repeat units, the base composition of the repeat units, the structure of the repeat sequences, and the number of repeats can all affect locus stability to some extent. MSI is significant in the selection of chemotherapy drugs and the screening of beneficiaries of immune checkpoint inhibitors [37]. TMB typically refers to the number of somatic non-synonymous mutations or all mutations per megabase in a tumor sample (tumor tissue or peripheral blood) detected through whole-exome sequencing or targeted sequencing. TMB can indirectly reflect the tumor’s ability and degree to produce new antigens and has been proven to predict the efficacy of immunotherapy in various tumors [38]. In our study, in both TMB and MSI analyses, LUAD patients with high LHRGs scores exhibited high inhibitory immune checkpoint expression and high TMB. Therefore, this prognosis model and its risk score may have significant value in accurately predicting which patients will respond to immunotherapy. Individual genes in the model, however, showed no statistically significant correlation with MSI in LUAD samples, which may suggest a possible synergistic effect between the five genes in immunotherapy. The mutation of driver genes in LUAD are widely different between high and low risk groups, suggesting that genes with high mutation probability can be focused on by combining risk scores during clinical diagnosis and treatment to enhance therapeutic efficacy and thus improve prognosis.

The five genes associated with this prognostic model were differentially expressed in the esophageal cancer、gastric carcinoma、clear cell carcinoma of the kidney、squamous lung cancer、hepatocellular carcinoma、adenocarcinoma of the rectum and cholangiocarcinoma, and nearly all of them were expressed in the tumor tissues higher than normal tissues. Among them, BCAN was less expressed in the above tumor types. There have been numerous reports on the significant impact of genes on the composition of prognosis models for lung cancer. Research and analysis have shown that PFKP is highly expressed in lung cancer tissues and cell lines and that its high expression is associated with poor prognosis. In lung cancer cell lines with decreased PFKP expression, the glucose uptake rate, lactate level, and adenosine triphosphate concentration are significantly reduced. These cell lines also exhibit a significantly reduced proliferation rate and colony formation ability and an increased percentage of cells in the G2/M phase of the cell cycle [39]. PFKP can regulate the expression of ABCC2 by activating NF-κB, thereby promoting chemotherapy resistance in NSCLC [40]. Additionally, PFKP regulates long-chain fatty acid oxidation through AMPK to alleviate metabolic stress induced by glucose starvation in non-small cell lung cancer cells. SLC2A1 is a glucose transporter protein expressed in many tissues of the human body. It controls the fundamental movement of hexose sugars in different cell types and plays a role in regulating the energy metabolism of tumors. Increased levels of SLC2A1 enhance the absorption of glucose in cancer cells, supplying ample resources for glycolysis. In addition to fuelling tumors, the fatty acids and nucleic acids generated in this process also support the growth and spread of tumors [41–43]. Patients with high ANLN expression had significantly more metastases than those with low ANLN expression. In vitro, interference with ANLN expression increases the expression of E-cadherin and vimentin in A549 and PC9 cells while decreasing the expression of N-cadherin [44]. The migration and invasion abilities of A549 and PC9 cells are reduced, and vice versa. There is a strong correlation between ANLN and epithelial–mesenchymal transition, which may affect invasion and migration through EMT. Fan reported that overexpression of CDKN3 leads to a worse overall survival (OS) prognosis in patients with LUAD. The overexpression of CDKN3 in lung adenocarcinoma is not attributed to selective splicing or mutation but may be due to increased mitotic activity, thus acting as a tumor suppressor [45]. Gao et al. confirmed through their study that depletion of CDKN3 significantly reduces the proliferation, invasion, migration, angiogenesis, EMT, and tumorigenicity of non-small cell lung cancer tumor cells while increasing apoptosis. This finding also confirms the tumor-suppressive effect of CDKN3 [46].

In this study, a systematic analysis of LUAD data from public database was performed to assess the predictive value of hypoxia and lactate metabolism for the prognosis of patients with LUAD. By applying the risk model provided by the model, clinicians can assess the current as well as future disease states of patients based on the gene expression levels associated with the model and the nomogram proposed in this study as a way to improve the accuracy of clinical treatment decisions. However, this study has several limitations. First, the prognostic model constructed using TCGA data had an AUC of less than 0.70 at 1 year in the training cohort, and second, there was a discrepancy in the number of tumor tissue samples versus normal tissue samples in the TCGA data included in this study; however, this discrepancy was due to the limitations of the original TCGA samples. To address this issue, multiple validation sets were included in this study to compensate for the uneven distribution of TCGA data. The predictive ability of this model for tumor metastasis and metastatic destination in lung adenocarcinoma patients is still insufficient. Targeted in-depth analyses of relevant patient samples are needed in the future. Finally, the synergistic effects of hypoxia and lactate metabolism on LUAD prognosis need to be further investigated, and in the case of the present model, additional external experiments are needed to confirm the identified model-related genes and their prognostic value.

## Conclusion

This is the first study to systematically analyze online open database information to summarize the predictive value of the synergy of genes related to hypoxia and lactate metabolism for the prognosis of LUAD patients. This study identified and constructed a prognostic model consisting of 5 genes (PKFP, SLC2A1, BCAN, CDKN3 and ANLN) and accompanying nomograms that could predict the survival and prognosis of patients with LUAD. It is also regarded as a marker of relevant immune checkpoint blockade (ICB), antitumor drug sensitivity and tumor immunological characteristics. This study has the potential to provide new perspectives for precision treatment of LUAD.

### Electronic supplementary material

Below is the link to the electronic supplementary material.

Supplementary Material 1

## Data Availability

The data in this study are freely available from the TCGA database (https://portal.gdc.cancer.gov/) and the GEO data portal (https://www.ncbi.nlm.nih.gov/geo/).
