# Supplementary material for: Identification of a combined hypoxia and lactate metabolism prognostic signature in lung adenocarcinoma
Source: BMC Pulm Med. 2024 Jul 4;24:323. doi: 10.1186/s12890-024-03132-4 (PMC11225160; doi:10.1186/s12890-024-03132-4)
Supplement: Supplementary file 1 — Supplementary Material 1 [file 12890_2024_3132_MOESM1_ESM.docx]

**Supplementary material**

**Supplementary figure 1：**

**
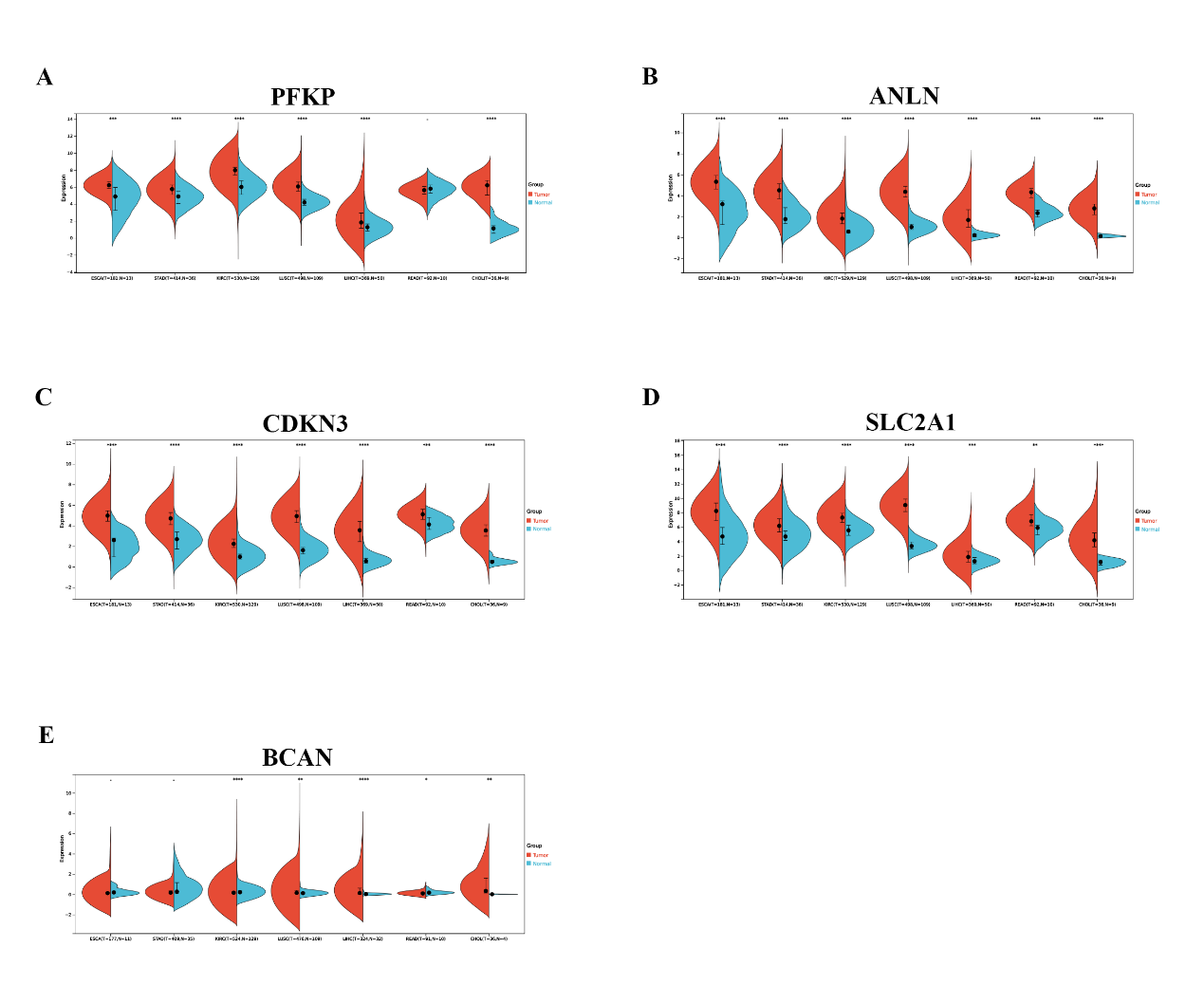
**

Differential expression of LH prognostic signature-related genes in ESCA, STAD, KIRC, LUSC, LIHC, READ and CHOL. (A) PFKP, (B) ANLN, (C) CDKN3, (D) SLC2A1, (E) BCAN.

**Supplementary figure 2：**


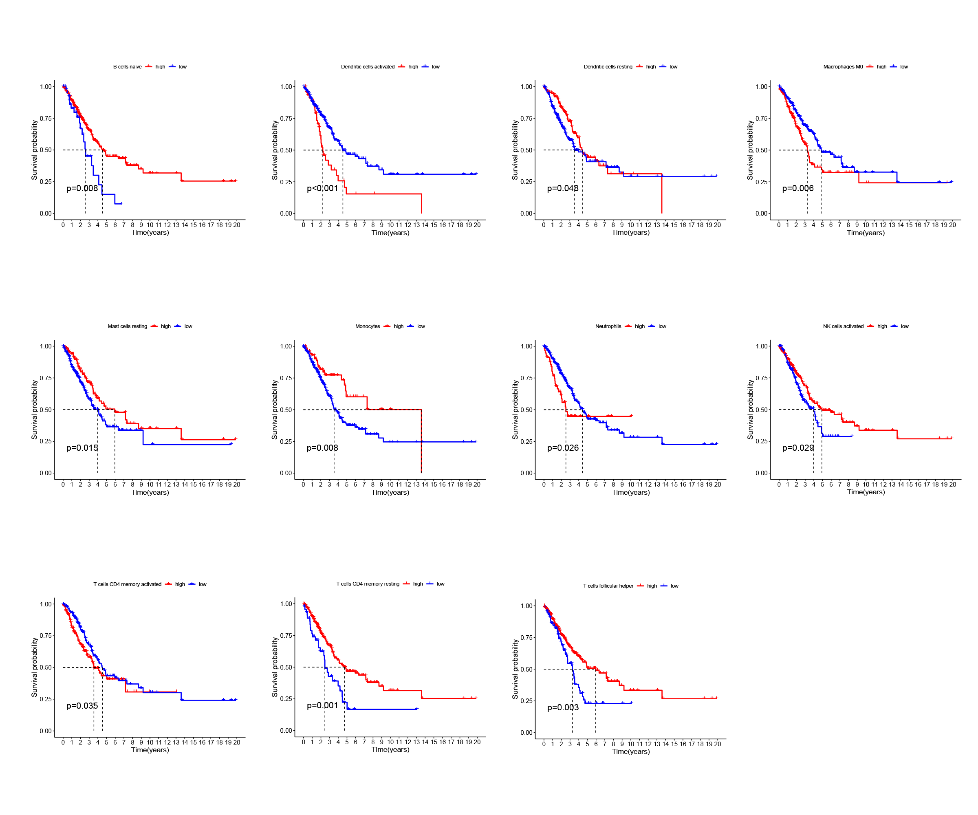


K-M Survival analysis based on CIBERSORT immune cell infiltration.

**Supplementary figure 3：**


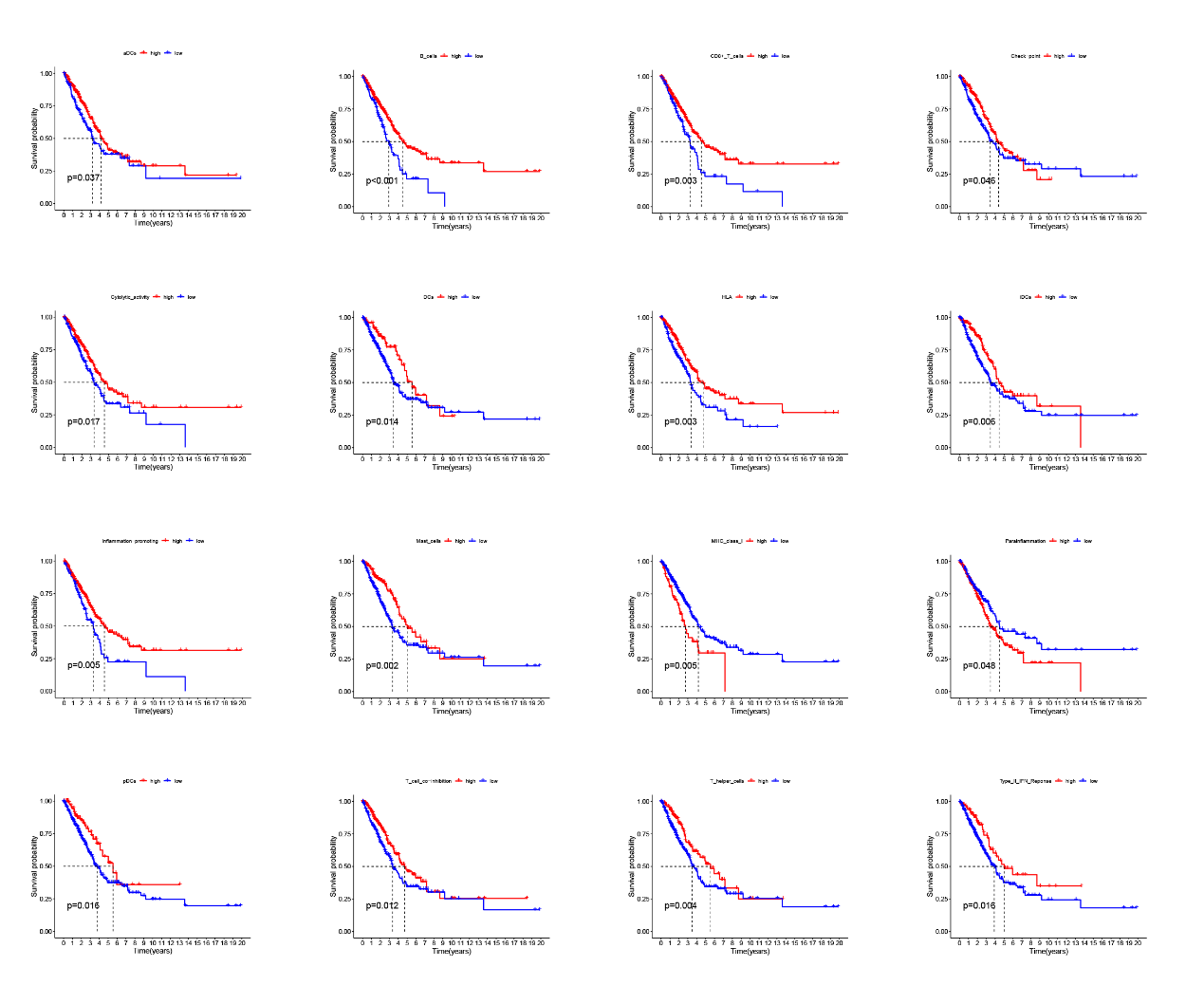


K-M Survival analysis based on ssGSEA immune function analysis.

**Supplementary figure 4：**


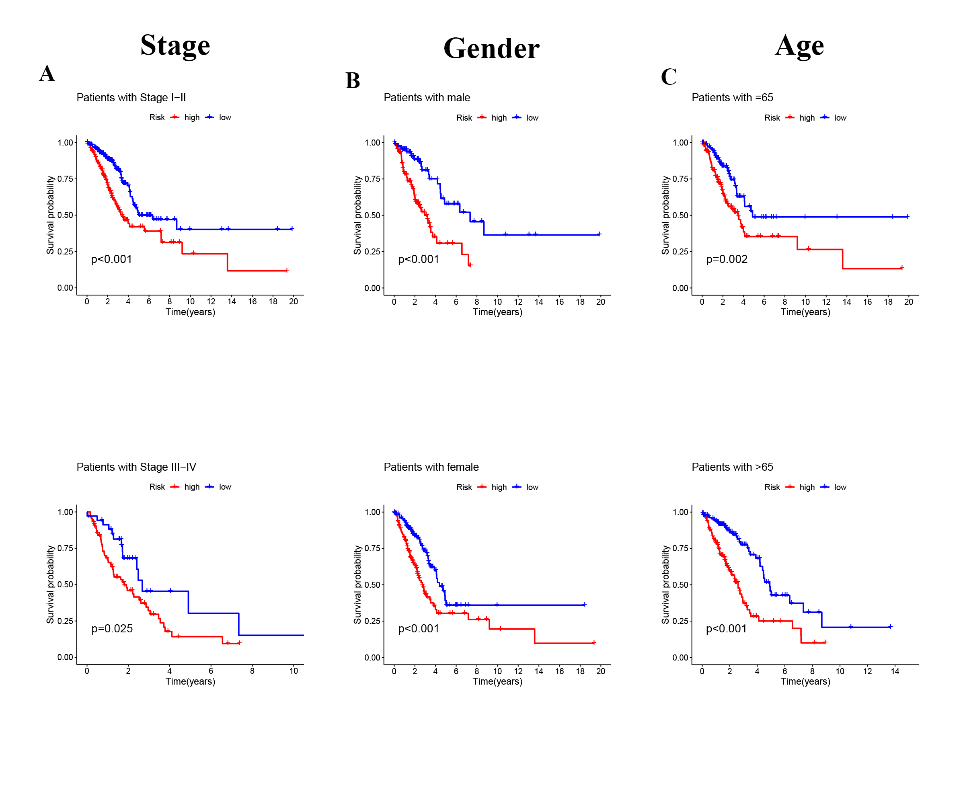


K-M curves of low/high risk groups between different clinical variables. (A) Stage I-II and stage III-IV. (B) Male and female. (C) Age <65 and age≥65 years old.

**Supplementary figure 5：**


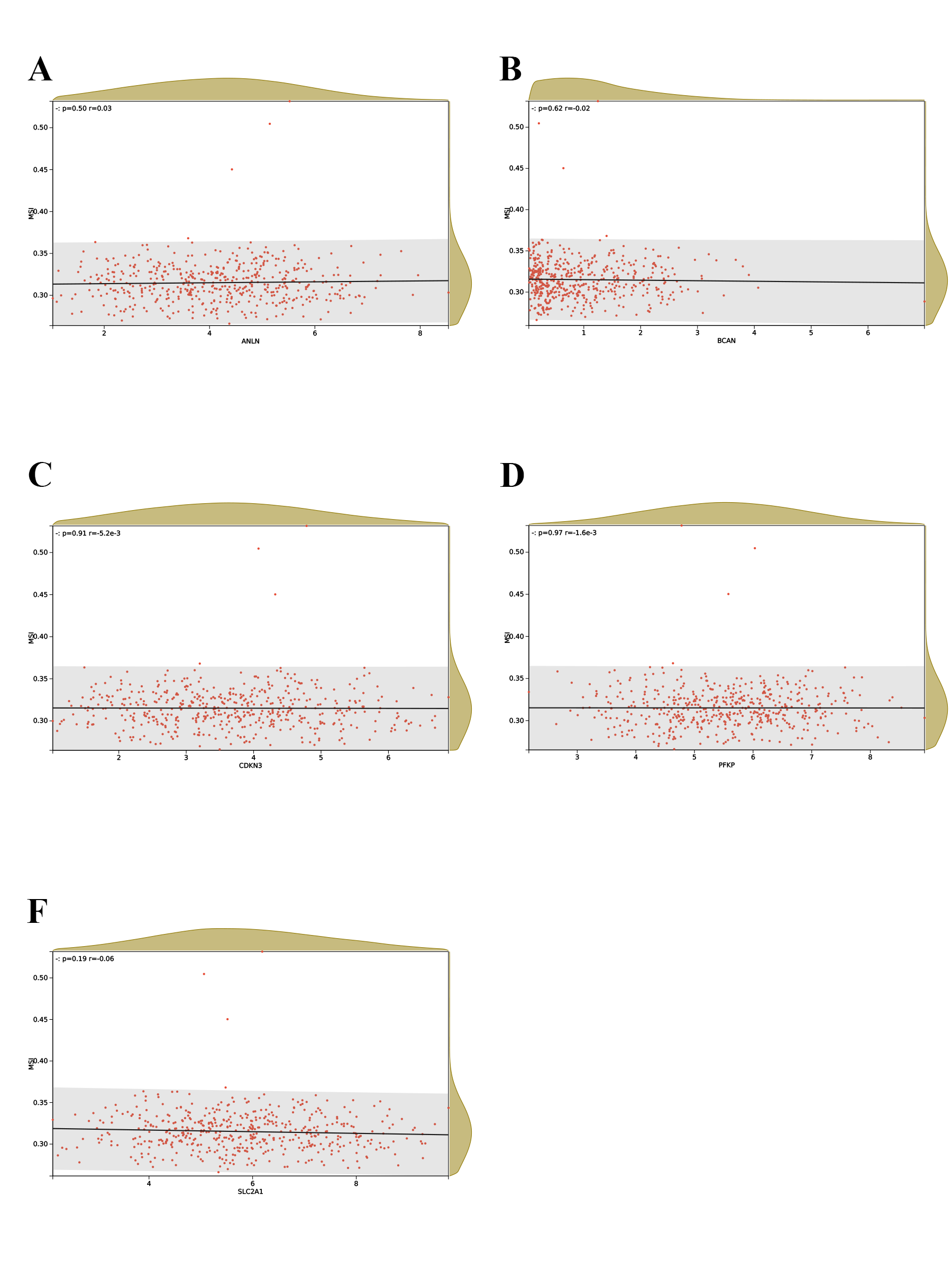


Microsatellite instability of each single genes in LUAD samples. (A) ANLN, (B) BCAN, (C) CDKN3, (D) PFKP, (E) SLC2A1.

**Supplementary figure 6：**


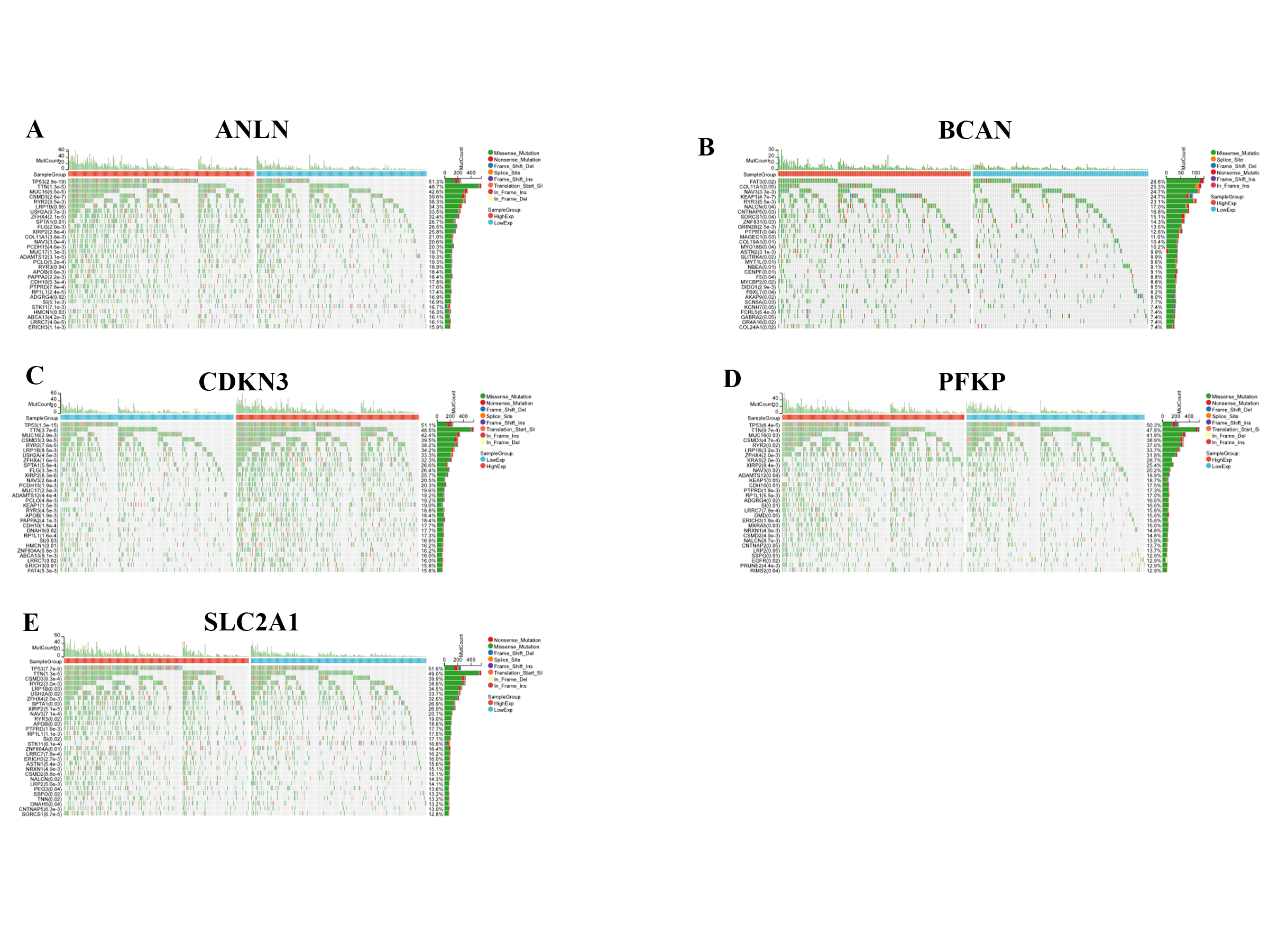


Somatic mutations of single genes in LUAD samples. (A) ANLN, (B) BCAN, (C) CDKN3, (D) PFKP, (E) SLC2A1.

**Supplementary figure 7：**


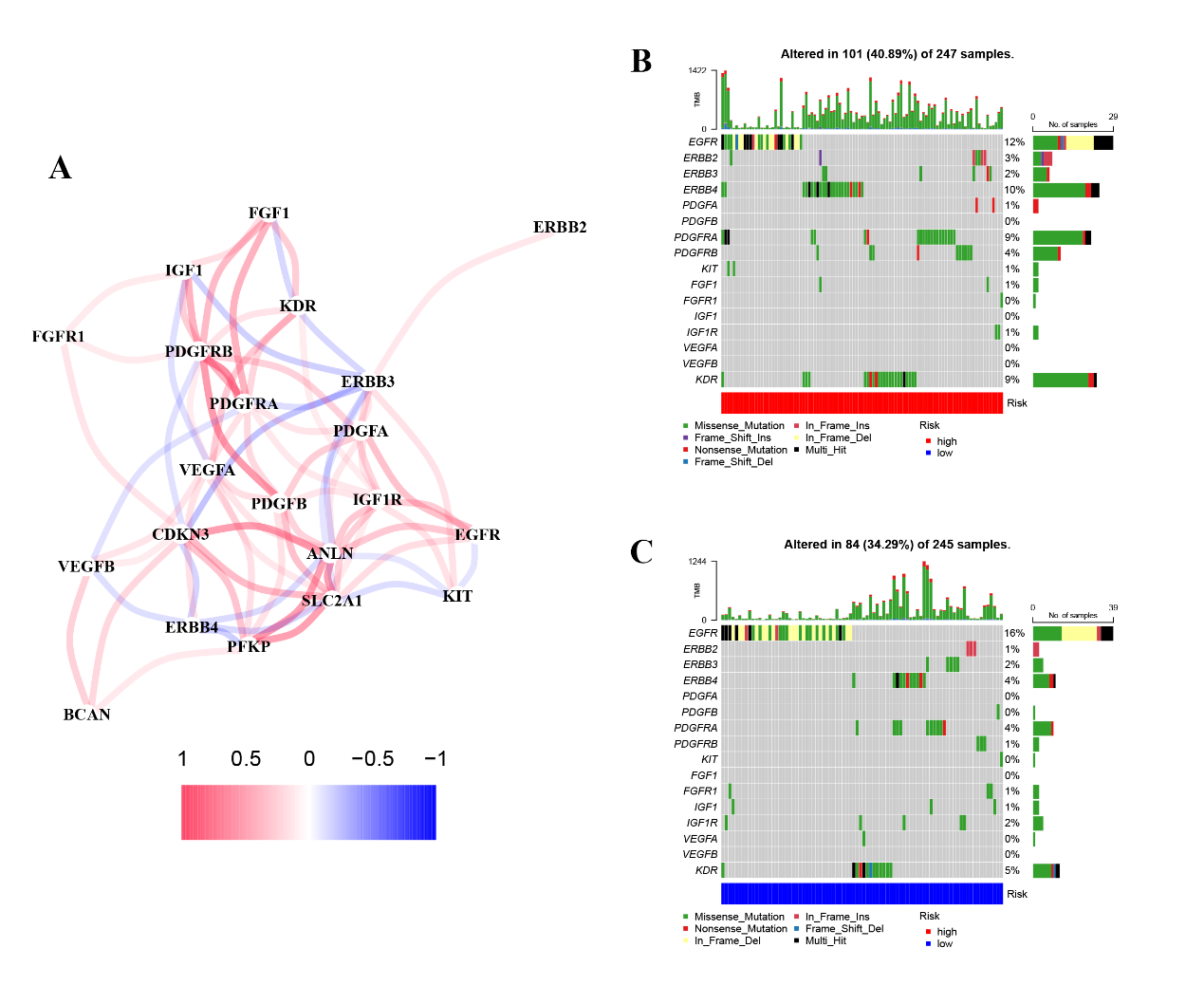


RTK pathway correlation networks and mutations in prognostic signature. (A) Correlation network. (B) High-risk mutation, (C) Low-risk mutation.

**Supplementary figure 8：**


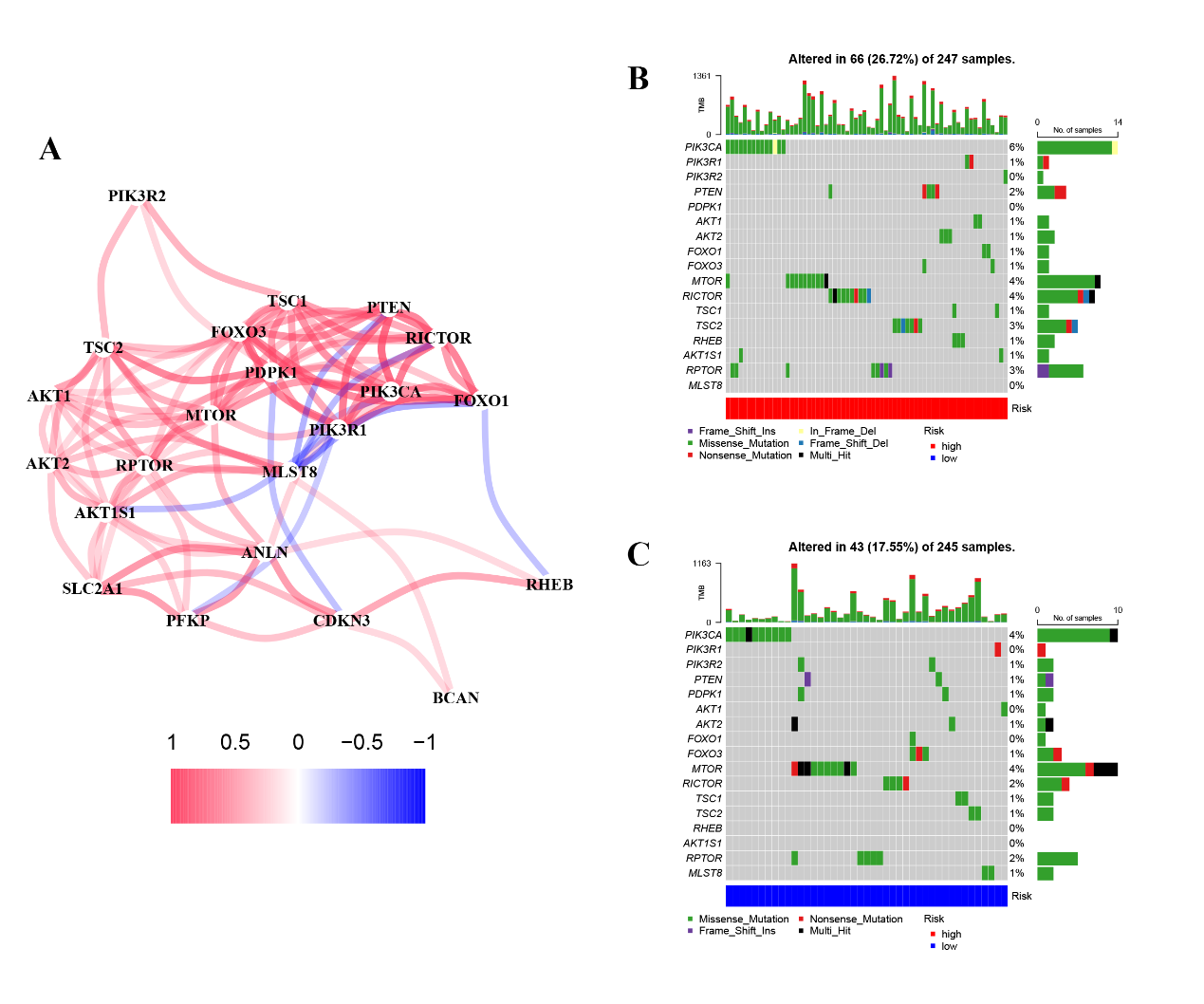


PI3K-AKT-mTOR pathway correlation networks and mutations in prognostic signature. (A) Correlation network. (B) High-risk mutation, (C) Low-risk mutation.

**Supplementary figure 9：**


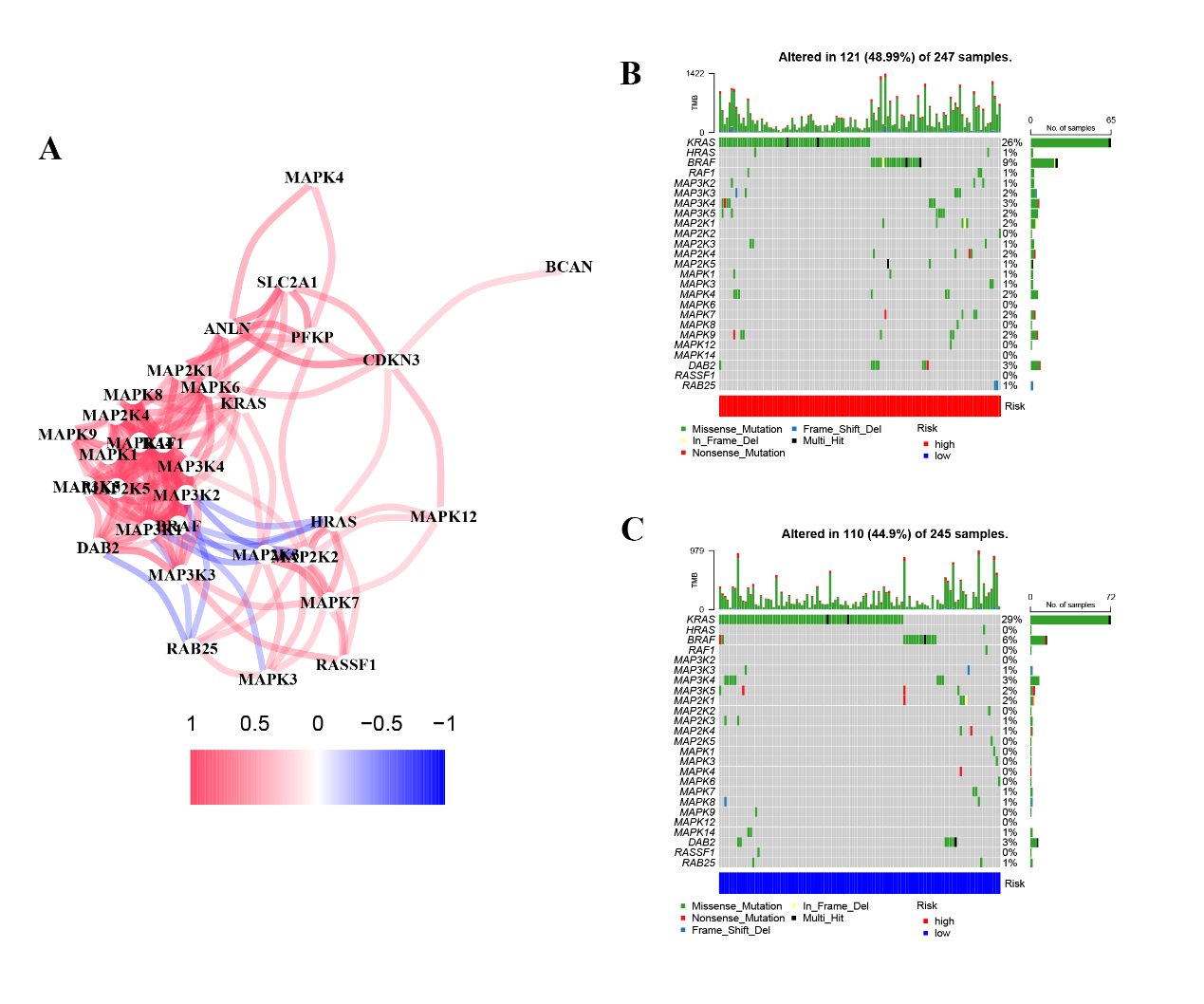


Ras-Raf-Mek-Erk pathway correlation networks and mutations in prognostic signature. (A) Correlation network. (B) High-risk mutation, (C) Low-risk mutation.

Table title:

Supplementary Table 1 Lung Adenocarcinoma Sample Baseline Information

| Type | **TCGA-train group** | | | **GEO-test group** | | |
| --- | --- | --- | --- | --- | --- | --- |
| Sex | Male | 230 | 45% | Male | 411 | 47.56% |
|  | Female | 280 | 54.90% | Female | 453 | 52.43% |
| T stage | T1 | 172 | 33.70% | stageI | 545 | 63.07% |
|  | T2 | 281 | 55.10% |  |  |  |
|  | T3 | 47 | 9.21% |  |  |  |
|  | T4 | 10 | 1.99% | stageII | 159 | 18.40% |
| N stage | N0 | 335 | 65.68% |  |  |  |
|  | N1 | 98 | 19.21% |  |  |  |
|  | N2 | 75 | 14.70% | stageIII | 92 | 10.64% |
|  | N3 | 2 | 0.39% |  |  |  |
|  | Nx | 13 | 2.54% |  |  |  |
| M stage | M0 | 353 | 69.21% | stageIV | 1 | 0.11% |
|  | M1 | 25 | 4.90% |  |  |  |
|  | Mx | 140 | 27.45% | unknow | 67 | 7.15% |

Supplementary Table 1 Legend: Baseline information from lung adenocarcinoma samples in TCGA and GEO database.

Supplementary table 2: Risk-related differential expressed genes based on TCGA-training group

| **gene** | **lowMean** | **highMean** | **logFC** | **pValue** | **fdr** |
| --- | --- | --- | --- | --- | --- |
| TCF21 | 4.694938228 | 1.844515538 | -1.347864234 | 1.95753E-13 | 2.01054E-12 |
| FAM166B | 8.971439286 | 3.006853519 | -1.577084867 | 1.15049E-05 | 3.47423E-05 |
| ELFN1-AS1 | 1.287446693 | 4.304661952 | 1.741387248 | 2.64735E-06 | 8.93692E-06 |
| MAP6 | 5.367147884 | 2.616451195 | -1.036544291 | 1.80471E-17 | 3.34236E-16 |
| GINS2 | 7.577646164 | 18.97917218 | 1.324595387 | 3.04604E-34 | 2.71941E-32 |
| LINC02253 | 0.617745899 | 1.620781408 | 1.391604096 | 0.006585496 | 0.011676026 |
| AC141273.2 | 3.285471164 | 0.879224303 | -1.90179711 | 1.36707E-13 | 1.43716E-12 |
| NEK5 | 2.021920767 | 0.957474635 | -1.078420291 | 1.75529E-06 | 6.11768E-06 |
| ADGRF4 | 3.183697619 | 9.804826428 | 1.622788773 | 6.14222E-25 | 2.78463E-23 |
| KIF11 | 8.877554365 | 26.90263958 | 1.599513536 | 1.68504E-54 | 1.22228E-51 |
| FAM107A | 9.899725397 | 4.626296547 | -1.097530761 | 7.08367E-15 | 9.10781E-14 |
| SOX15 | 2.980565344 | 8.880932736 | 1.575125204 | 1.12482E-05 | 3.40853E-05 |
| BIRC5 | 17.63172235 | 56.82155199 | 1.688264826 | 1.71145E-49 | 4.88437E-47 |
| ERO1A | 57.20321257 | 135.0724978 | 1.23956588 | 3.90869E-39 | 4.59773E-37 |
| PLAU | 95.59052817 | 235.1168258 | 1.29843821 | 7.91057E-13 | 7.42E-12 |
| MKI67 | 9.677800661 | 33.77377112 | 1.803152148 | 4.77221E-52 | 1.84621E-49 |
| LINC01269 | 1.759829762 | 3.884477756 | 1.142284774 | 1.82373E-08 | 8.79483E-08 |
| FOSB | 85.00734206 | 37.3928492 | -1.184825048 | 9.45298E-05 | 0.000242474 |
| PMAIP1 | 19.41747817 | 48.70598074 | 1.326743091 | 4.87999E-25 | 2.22981E-23 |
| KPNA2 | 28.86497817 | 75.27662397 | 1.382881793 | 3.63846E-54 | 2.26221E-51 |
| PTCHD4 | 1.818794841 | 0.845296149 | -1.105454035 | 1.41862E-10 | 9.43702E-10 |
| NXPH4 | 4.660149471 | 14.37861501 | 1.625476583 | 6.87401E-05 | 0.000180824 |
| EZH2 | 10.41779577 | 22.64785704 | 1.120324488 | 6.36184E-36 | 6.22209E-34 |
| ZDHHC11B | 8.878960714 | 3.891679748 | -1.189997825 | 1.75097E-19 | 4.17001E-18 |
| LINC00337 | 0.571514815 | 1.488510491 | 1.381006588 | 1.09064E-25 | 5.28884E-24 |
| SEC14L6 | 9.638653307 | 3.989342629 | -1.272680554 | 2.51164E-15 | 3.473E-14 |
| DLEC1 | 3.757001455 | 1.21534077 | -1.628220786 | 5.78998E-15 | 7.55039E-14 |
| CASC15 | 0.627325794 | 1.487732802 | 1.245828653 | 1.13867E-07 | 4.88496E-07 |
| GGT2 | 1.632355291 | 0.747868393 | -1.126098784 | 1.00051E-13 | 1.06989E-12 |
| CEBPA | 20.27323902 | 9.515758035 | -1.091186111 | 6.76166E-19 | 1.50337E-17 |
| SLC2A1 | 39.49571019 | 151.4333965 | 1.938915537 | 2.6824E-47 | 5.69486E-45 |
| SERPINB3 | 4.985056746 | 18.1581413 | 1.864934701 | 8.25263E-05 | 0.000213922 |
| SLC16A3 | 35.92443942 | 74.88900432 | 1.059788264 | 1.4486E-22 | 5.07418E-21 |
| MIR4284 | 1.137318254 | 2.338440305 | 1.039910583 | 4.45105E-05 | 0.000120679 |
| KCNJ15 | 23.83992884 | 11.38933028 | -1.065697014 | 2.75725E-11 | 2.04346E-10 |
| AL161431.1 | 0.982253704 | 4.748835193 | 2.273406081 | 0.000461903 | 0.001042697 |
| CCNA2 | 9.722806349 | 36.54356155 | 1.910172554 | 1.53317E-59 | 8.89701E-56 |
| ATP13A4-AS1 | 7.998790608 | 2.992332072 | -1.4185116 | 3.24988E-10 | 2.05362E-09 |
| SCNN1G | 19.79075847 | 9.637557238 | -1.038087475 | 1.12542E-12 | 1.03227E-11 |
| CAPN3 | 2.293175661 | 0.957416866 | -1.260127746 | 1.44464E-13 | 1.51504E-12 |
| CDK5R1 | 3.678255556 | 7.777622444 | 1.080307484 | 4.46104E-20 | 1.15914E-18 |
| CSAG1 | 4.794031746 | 20.76776713 | 2.115034744 | 5.5069E-09 | 2.88243E-08 |
| MORN5 | 8.258497222 | 2.222443426 | -1.893732587 | 2.1435E-06 | 7.34572E-06 |
| ANKRD45 | 1.508564815 | 0.705048938 | -1.097381379 | 4.73411E-10 | 2.92359E-09 |
| FBXO32 | 19.67768995 | 39.68632649 | 1.012081161 | 9.06788E-15 | 1.14145E-13 |
| RNA5SP18 | 1.832759392 | 4.151010292 | 1.179445111 | 1.61018E-11 | 1.24144E-10 |
| HSD17B6 | 56.71170635 | 14.89187709 | -1.929120951 | 8.00006E-18 | 1.56487E-16 |
| DCBLD2 | 23.16943704 | 58.11621295 | 1.326717705 | 6.22983E-09 | 3.23168E-08 |
| EPGN | 0.606860847 | 1.728455511 | 1.510045822 | 0.02330144 | 0.036235352 |
| ARNTL2 | 7.517883069 | 25.58720684 | 1.767024287 | 7.70859E-28 | 4.67592E-26 |
| HLA-DQB2 | 172.6518 | 73.7565587 | -1.227022125 | 1.22649E-09 | 7.15309E-09 |
| ADGRF5 | 181.0236177 | 78.44835936 | -1.206362754 | 1.7234E-21 | 5.31962E-20 |
| CIDEC | 0.82916746 | 1.918630876 | 1.210341774 | 0.003599461 | 0.006761953 |
| SCGB1A1 | 450.8725519 | 75.84132736 | -2.571663565 | 6.72772E-11 | 4.70751E-10 |
| LHFPL3-AS2 | 13.34802354 | 3.536439509 | -1.916256647 | 6.1292E-21 | 1.77839E-19 |
| STRIP2 | 4.250506217 | 9.756186587 | 1.19868268 | 3.61873E-24 | 1.47193E-22 |
| DES | 18.48234788 | 6.818403054 | -1.438642251 | 1.72992E-17 | 3.20727E-16 |
| HHIP-AS1 | 7.497596164 | 3.628070983 | -1.047225439 | 8.28214E-09 | 4.21962E-08 |
| BUB1B | 4.320068519 | 15.22549482 | 1.817363016 | 1.8649E-56 | 3.2466E-53 |
| COL7A1 | 5.143135847 | 12.54470797 | 1.286358721 | 2.08099E-12 | 1.83899E-11 |
| ROBO2 | 2.278451058 | 0.855718194 | -1.412845711 | 1.02148E-18 | 2.22565E-17 |
| INCENP | 12.09325132 | 26.65657683 | 1.140289353 | 1.89806E-42 | 2.70847E-40 |
| AL157871.3 | 1.390965873 | 3.159329482 | 1.183531377 | 0.020411034 | 0.032157076 |
| KIF4A | 6.533419577 | 24.2082245 | 1.889587074 | 1.45162E-57 | 4.69371E-54 |
| CCDC170 | 7.046678571 | 3.143960956 | -1.164360107 | 7.48365E-08 | 3.30081E-07 |
| POPDC3 | 1.027053175 | 5.330380412 | 2.375727619 | 2.34979E-12 | 2.06083E-11 |
| ASPH | 44.57444974 | 99.93187882 | 1.164727991 | 2.24109E-13 | 2.27893E-12 |
| A2ML1 | 0.313190873 | 5.194987317 | 4.052006152 | 0.001555278 | 0.003156428 |
| CDCA2 | 2.761399868 | 8.903977955 | 1.689050207 | 1.75859E-52 | 7.11984E-50 |
| PCSK2 | 79.19614815 | 5.076460027 | -3.963535547 | 9.74532E-11 | 6.65319E-10 |
| GAS2L2 | 4.149530159 | 1.557322908 | -1.413879877 | 7.02002E-12 | 5.75655E-11 |
| ESYT3 | 9.488457275 | 4.298652191 | -1.142289154 | 2.27219E-20 | 6.17106E-19 |
| GTSE1 | 4.77258254 | 14.29029675 | 1.582193821 | 1.41676E-48 | 3.52349E-46 |
| LINC00942 | 7.855644312 | 32.68330883 | 2.056752534 | 1.08079E-07 | 4.64807E-07 |
| AC022497.1 | 2.770677646 | 1.154052855 | -1.26352957 | 1.3535E-20 | 3.78828E-19 |
| DAPK2 | 6.442653571 | 3.183119721 | -1.017213602 | 1.32643E-20 | 3.7185E-19 |
| ENKUR | 4.255753307 | 1.926022311 | -1.143790109 | 0.009226939 | 0.015804002 |
| CENPU | 9.236784656 | 23.05555478 | 1.319651742 | 8.10292E-42 | 1.08511E-39 |
| LINC00460 | 1.189116931 | 4.211109363 | 1.824309754 | 0.010605808 | 0.017901091 |
| PTGES | 52.08844841 | 108.4081163 | 1.057437404 | 1.21779E-07 | 5.18984E-07 |
| FAM111B | 6.14267791 | 16.75911853 | 1.448006627 | 2.61953E-39 | 3.10227E-37 |
| KIF20B | 4.562740476 | 10.68996421 | 1.228284521 | 2.06567E-44 | 3.39257E-42 |
| WFDC5 | 19.55761217 | 0.803725299 | -4.604883935 | 9.34082E-05 | 0.000239773 |
| CNMD | 62.83989008 | 4.250532138 | -3.885965284 | 2.00479E-08 | 9.59621E-08 |
| H3C11 | 0.518375397 | 1.5546334 | 1.584505265 | 9.38271E-07 | 3.45116E-06 |
| AC012085.1 | 0.966705423 | 2.191997145 | 1.181097679 | 3.67304E-12 | 3.1185E-11 |
| CRYM | 40.96734577 | 12.57063533 | -1.704416859 | 2.92388E-20 | 7.79508E-19 |
| CLDN2 | 82.88049405 | 23.58974867 | -1.812872555 | 1.10967E-11 | 8.78103E-11 |
| LINC02561 | 0.82756746 | 2.016784728 | 1.285108273 | 2.64084E-06 | 8.91833E-06 |
| ARHGEF2-AS1 | 3.726593915 | 1.405473971 | -1.406800883 | 1.28776E-09 | 7.48786E-09 |
| TRIM16L | 8.162047354 | 18.04584402 | 1.144663635 | 8.49561E-09 | 4.32204E-08 |
| SKA1 | 3.195791931 | 11.37622683 | 1.831776749 | 1.07339E-52 | 4.67167E-50 |
| AC073149.1 | 2.210943519 | 1.059368061 | -1.061458252 | 5.32315E-10 | 3.26766E-09 |
| RTN4RL1 | 6.276774735 | 2.602471315 | -1.270141172 | 1.10211E-13 | 1.17349E-12 |
| RCOR2 | 3.661369444 | 8.639976029 | 1.238643956 | 1.61898E-14 | 1.97234E-13 |
| CFAP52 | 4.769121429 | 1.338476096 | -1.833132143 | 9.5393E-08 | 4.14242E-07 |
| PLIN5 | 3.709525926 | 1.704080279 | -1.122241521 | 2.90579E-16 | 4.55328E-15 |
| NLGN4X | 0.606801587 | 2.264180544 | 1.899692238 | 0.009554026 | 0.016316072 |
| AC008703.1 | 2.402972487 | 1.192653918 | -1.010644665 | 4.39818E-05 | 0.000119395 |
| RECQL4 | 11.08432778 | 23.42600279 | 1.079589527 | 2.01851E-28 | 1.2732E-26 |
| CASC9 | 2.830994444 | 6.430890505 | 1.183709608 | 1.42523E-07 | 5.99754E-07 |
| MAD2L1 | 5.844055291 | 19.25206521 | 1.719971482 | 2.43941E-55 | 2.4981E-52 |
| FBXO5 | 5.092599074 | 11.18022032 | 1.134474571 | 1.48789E-40 | 1.877E-38 |
| TEPP | 3.746364947 | 0.982574369 | -1.930852933 | 2.8472E-17 | 5.10472E-16 |
| ITIH5 | 5.973677646 | 2.800733732 | -1.092814557 | 2.13926E-20 | 5.81911E-19 |
| MCM7 | 63.50564061 | 133.8263373 | 1.075405426 | 2.4913E-36 | 2.49259E-34 |
| NAPSA | 1058.452163 | 424.9492949 | -1.316593454 | 5.75421E-24 | 2.29233E-22 |
| TYMS | 4.934877778 | 10.70143074 | 1.116717434 | 5.28462E-36 | 5.19773E-34 |
| SYBU | 20.99949312 | 8.406793692 | -1.320726931 | 3.67786E-06 | 1.2115E-05 |
| TOGARAM2 | 1.88097328 | 0.659532869 | -1.511962891 | 1.16381E-07 | 4.97931E-07 |
| PLA2G12B | 10.48041958 | 5.166612351 | -1.020405927 | 6.65026E-07 | 2.51137E-06 |
| CIP2A | 3.574264153 | 11.20445146 | 1.648353855 | 7.31114E-51 | 2.35703E-48 |
| VCX | 0.44182672 | 1.983537782 | 2.166523304 | 0.019153527 | 0.03035371 |
| AC007255.1 | 0.711999206 | 1.444653453 | 1.020775919 | 1.35818E-05 | 4.03491E-05 |
| NR4A1AS | 5.219749603 | 2.275602922 | -1.197731762 | 1.92753E-07 | 7.94423E-07 |
| TTC25 | 6.088970635 | 3.011232802 | -1.015844106 | 7.32535E-06 | 2.29737E-05 |
| BORA | 3.208291931 | 6.649466135 | 1.051433093 | 1.66493E-34 | 1.50962E-32 |
| GNG4 | 2.826720635 | 12.64848154 | 2.161762985 | 2.27672E-17 | 4.15906E-16 |
| AURKB | 11.30183386 | 32.67380837 | 1.531577734 | 1.49179E-43 | 2.31881E-41 |
| AC113349.1 | 1.79152791 | 0.409018526 | -2.130952425 | 2.84244E-08 | 1.33093E-07 |
| CCL7 | 2.576988889 | 6.169985525 | 1.259580788 | 3.78286E-15 | 5.1051E-14 |
| HAGLR | 43.70646839 | 19.71941554 | -1.148230016 | 7.89997E-15 | 1.00534E-13 |
| NECAB2 | 0.710518651 | 2.047058167 | 1.526607672 | 1.36175E-09 | 7.88385E-09 |
| HOPX | 225.7562878 | 87.60335657 | -1.365708117 | 4.96615E-15 | 6.55464E-14 |
| SFTPA2 | 6052.970161 | 2588.856634 | -1.225328165 | 6.57069E-11 | 4.60134E-10 |
| RAB3B | 0.802853175 | 4.375922776 | 2.446379201 | 4.98402E-20 | 1.28543E-18 |
| AC112777.1 | 0.796271429 | 2.362793161 | 1.569161144 | 1.11762E-38 | 1.28852E-36 |
| IGSF9B | 4.276057672 | 2.110007902 | -1.019032909 | 9.1451E-11 | 6.27544E-10 |
| COL11A1 | 17.25184524 | 38.43490551 | 1.155666443 | 1.81047E-13 | 1.87164E-12 |
| AC107021.2 | 0.71805 | 1.654471979 | 1.204214646 | 8.48191E-12 | 6.85523E-11 |
| PTPRH | 8.571619048 | 17.82844283 | 1.056541062 | 9.87589E-16 | 1.43994E-14 |
| LEFTY2 | 1.88064246 | 0.616674236 | -1.608645117 | 9.36925E-23 | 3.32734E-21 |
| FAM83A | 37.50243492 | 114.7919011 | 1.613964685 | 7.56432E-20 | 1.89478E-18 |
| INA | 2.21391918 | 6.200110226 | 1.485691307 | 0.000428767 | 0.000972815 |
| RSPH1 | 13.95678651 | 5.576072444 | -1.323645596 | 1.11658E-08 | 5.59063E-08 |
| E2F2 | 2.767215741 | 6.599922112 | 1.254013873 | 2.49436E-33 | 2.12864E-31 |
| CYP4B1 | 169.9956729 | 32.84288028 | -2.371845464 | 2.59428E-30 | 1.93836E-28 |
| GAPDHP65 | 1.468778571 | 3.395900133 | 1.209177117 | 1.20175E-19 | 2.93427E-18 |
| RNASE1 | 2564.756652 | 969.1941823 | -1.403964297 | 2.3753E-26 | 1.24179E-24 |
| LRRC46 | 11.41979841 | 4.435795086 | -1.364272558 | 0.000644488 | 0.00141255 |
| IGHV1-45 | 31.89424074 | 15.21001474 | -1.068274384 | 3.36982E-05 | 9.31194E-05 |
| UGT8 | 7.785467857 | 15.73241833 | 1.014884809 | 7.72875E-09 | 3.95618E-08 |
| KRT6A | 43.65837963 | 180.0976986 | 2.044449255 | 2.26781E-09 | 1.26865E-08 |
| RACGAP1 | 15.38269616 | 40.78512523 | 1.406734692 | 2.65108E-51 | 9.04953E-49 |
| IL23A | 5.211468519 | 11.44512961 | 1.134971935 | 5.15576E-09 | 2.71578E-08 |
| HOATZ | 2.541205952 | 0.69492178 | -1.870590801 | 1.02432E-06 | 3.73455E-06 |
| CCDC78 | 7.69253082 | 3.551253519 | -1.115129963 | 0.001407373 | 0.00288518 |
| MAST1 | 0.776352778 | 1.747978619 | 1.170903265 | 1.04303E-14 | 1.30353E-13 |
| IRX1 | 4.201158466 | 2.046187981 | -1.037848514 | 1.83047E-12 | 1.63587E-11 |
| PSRC1 | 5.987856746 | 12.89384861 | 1.106571337 | 1.17512E-34 | 1.07108E-32 |
| PLA2G1B | 40.59359709 | 7.551948207 | -2.426331411 | 6.51849E-24 | 2.59088E-22 |
| KCND2 | 1.129162037 | 2.362448805 | 1.065030535 | 9.02412E-15 | 1.13676E-13 |
| CENPH | 8.65681045 | 18.41439177 | 1.08892627 | 8.10165E-40 | 9.86305E-38 |
| SPAG8 | 3.520285053 | 1.433903984 | -1.295743832 | 5.58742E-11 | 3.95895E-10 |
| SP6 | 3.133878307 | 7.645020717 | 1.286571252 | 2.36616E-14 | 2.79271E-13 |
| MSLN | 898.7553954 | 386.1678521 | -1.218700459 | 2.4744E-05 | 7.01235E-05 |
| IL5RA | 1.577437698 | 0.55320166 | -1.511705636 | 6.33267E-11 | 4.44538E-10 |
| CENPF | 7.968091138 | 24.80743367 | 1.638466442 | 5.91711E-46 | 1.11969E-43 |
| CASQ2 | 1.735110053 | 0.760280013 | -1.190424401 | 1.59351E-17 | 2.97655E-16 |
| MTFR2 | 2.861729101 | 8.302362417 | 1.536634801 | 2.24292E-51 | 7.8094E-49 |
| MCM6 | 30.31079907 | 63.94095505 | 1.076908407 | 1.53048E-43 | 2.3372E-41 |
| UBE2S | 13.98910317 | 35.18543951 | 1.330675058 | 2.65828E-37 | 2.73835E-35 |
| GINS1 | 8.134958598 | 18.46243274 | 1.182385756 | 1.3271E-37 | 1.40021E-35 |
| AKAP14 | 3.174722751 | 0.945613413 | -1.747308202 | 2.34926E-06 | 8.00044E-06 |
| SPEF1 | 5.247793122 | 2.121799734 | -1.306422353 | 5.48863E-09 | 2.87462E-08 |
| AC026785.3 | 1.868778175 | 4.92794243 | 1.398890072 | 2.73816E-08 | 1.2866E-07 |
| ESCO2 | 1.302861508 | 3.465587649 | 1.411416271 | 2.5738E-43 | 3.82969E-41 |
| S100A7 | 3.852237963 | 45.47013001 | 3.561150399 | 2.36006E-09 | 1.31645E-08 |
| UBE2SP1 | 1.10383254 | 3.190896481 | 1.531440485 | 3.4972E-26 | 1.80661E-24 |
| INMT | 38.10564286 | 11.90787344 | -1.678088861 | 2.07548E-27 | 1.2044E-25 |
| LPAL2 | 1.745963228 | 0.811221315 | -1.105855709 | 5.39528E-18 | 1.08586E-16 |
| ADHFE1 | 1.946396429 | 0.91811421 | -1.084060043 | 3.98346E-17 | 6.97667E-16 |
| PCSK1 | 7.818709656 | 59.57985969 | 2.929822286 | 1.67299E-09 | 9.55546E-09 |
| KCP | 0.874086111 | 2.145280744 | 1.29531914 | 6.56265E-18 | 1.30423E-16 |
| ACTL8 | 0.495432804 | 2.654980611 | 2.421940023 | 7.56901E-09 | 3.87898E-08 |
| NPR1 | 12.65157579 | 6.012018592 | -1.073395712 | 1.57818E-10 | 1.04031E-09 |
| MAGEA3 | 6.571109921 | 34.33674714 | 2.385544393 | 6.32965E-09 | 3.27857E-08 |
| ITGA5 | 32.59039669 | 66.04324947 | 1.018964193 | 1.83776E-14 | 2.21562E-13 |
| ZNF385B | 15.32827394 | 4.688846746 | -1.708890219 | 1.57417E-11 | 1.21583E-10 |
| PKMYT1 | 3.860332143 | 10.6368166 | 1.462269556 | 1.70131E-44 | 2.82078E-42 |
| LINC00519 | 0.720484921 | 1.511453652 | 1.068896599 | 2.91827E-05 | 8.16524E-05 |
| CENPW | 16.4450828 | 42.10739309 | 1.356417287 | 2.04749E-42 | 2.89794E-40 |
| SLC26A9 | 53.12589034 | 17.33051049 | -1.616100963 | 1.37946E-13 | 1.44931E-12 |
| CA4 | 4.827526984 | 1.642216401 | -1.555640074 | 4.40253E-07 | 1.71232E-06 |
| TSPAN19 | 2.337719312 | 0.614260425 | -1.928179374 | 0.001817098 | 0.003630822 |
| FADS6 | 2.05533664 | 0.979905246 | -1.068660554 | 2.88317E-06 | 9.67112E-06 |
| SCGB2A1 | 31.52914722 | 9.866234993 | -1.676114595 | 5.49445E-09 | 2.87678E-08 |
| RNFT2 | 2.015085714 | 4.184406507 | 1.054181807 | 1.27231E-10 | 8.53881E-10 |
| GNMT | 3.415991931 | 1.500425033 | -1.186933329 | 2.21192E-16 | 3.50704E-15 |
| CDKN2A | 11.00716878 | 25.23090744 | 1.196748661 | 1.32966E-05 | 3.95759E-05 |
| Z99496.1 | 3.539383466 | 0.581571116 | -2.605470553 | 0.002221394 | 0.004361368 |
| ZYG11A | 1.598001852 | 3.252384993 | 1.025228963 | 2.19965E-11 | 1.65273E-10 |
| CCL8 | 11.34330146 | 25.54692789 | 1.171309216 | 8.72063E-08 | 3.80304E-07 |
| KRT16 | 9.314457143 | 34.28387337 | 1.879986519 | 1.23E-07 | 5.23591E-07 |
| CLIC5 | 15.95228955 | 7.534802058 | -1.082121984 | 1.20633E-13 | 1.27977E-12 |
| PDE10A | 1.229297619 | 2.934621713 | 1.255340303 | 7.50658E-06 | 2.34955E-05 |
| LINC00525 | 0.618338492 | 1.488625764 | 1.267512388 | 2.78597E-12 | 2.41299E-11 |
| ADAMTS8 | 6.303816799 | 1.985625963 | -1.666631723 | 5.22234E-24 | 2.09483E-22 |
| PCDH7 | 4.780791667 | 10.15066514 | 1.086252822 | 6.04975E-09 | 3.14576E-08 |
| AL445493.3 | 2.926116667 | 1.04929927 | -1.479561086 | 7.63653E-15 | 9.74666E-14 |
| TINAG | 1.071865212 | 2.741201527 | 1.354684899 | 0.00564851 | 0.010176438 |
| PLA2G2A | 48.41304471 | 10.59650332 | -2.191807552 | 1.2064E-06 | 4.3447E-06 |
| CYP4F11 | 7.407981085 | 16.13384449 | 1.122937935 | 0.025665529 | 0.039495376 |
| AL139041.1 | 3.352062963 | 1.497915405 | -1.162093098 | 3.4519E-14 | 3.95356E-13 |
| BZW1P2 | 2.531460317 | 5.291384927 | 1.063675503 | 1.70558E-11 | 1.30688E-10 |
| EID3 | 3.303669974 | 6.853548274 | 1.052781532 | 8.39797E-05 | 0.000217398 |
| C5orf34 | 2.29157209 | 5.07844004 | 1.148047736 | 6.82645E-35 | 6.35517E-33 |
| LINC02709 | 0.645035979 | 1.385549602 | 1.103006821 | 3.59601E-24 | 1.46611E-22 |
| TMPRSS11E | 10.70655847 | 34.92866932 | 1.705916869 | 7.06807E-12 | 5.79195E-11 |
| CAPS | 65.52128175 | 28.61359044 | -1.195263041 | 1.0817E-11 | 8.58312E-11 |
| CPS1 | 38.97100939 | 112.5191835 | 1.529697785 | 0.000179508 | 0.0004364 |
| DDIAS | 2.150390476 | 6.505763679 | 1.597119764 | 7.58001E-48 | 1.73632E-45 |
| AP001207.3 | 4.971748148 | 2.332741434 | -1.091726811 | 5.81812E-13 | 5.55915E-12 |
| SPRED3 | 1.356344312 | 2.891018991 | 1.091854628 | 2.29055E-19 | 5.39596E-18 |
| FCN3 | 21.2096418 | 10.14100817 | -1.064519171 | 1.10749E-05 | 3.36186E-05 |
| SBSN | 0.587724206 | 6.309836454 | 3.424391388 | 2.57096E-19 | 5.99169E-18 |
| ATP13A4 | 35.13823003 | 15.67293068 | -1.164766548 | 2.36378E-16 | 3.741E-15 |
| CFAP53 | 7.344992857 | 3.589807304 | -1.032854684 | 0.002506361 | 0.004864353 |
| CDKL2 | 16.43939762 | 7.415754117 | -1.148492121 | 1.26152E-14 | 1.55868E-13 |
| LDHC | 0.801449206 | 1.69392324 | 1.079685507 | 0.000416125 | 0.000946698 |
| SHOX2 | 0.694498545 | 3.147127756 | 2.179992168 | 1.3753E-19 | 3.31156E-18 |
| DTL | 6.683974206 | 16.71095498 | 1.32201611 | 1.37695E-44 | 2.30493E-42 |
| FENDRR | 2.927044048 | 1.27565996 | -1.198200641 | 2.91082E-09 | 1.59705E-08 |
| FAIM2 | 4.654023016 | 1.903722709 | -1.28965499 | 9.20642E-08 | 4.00687E-07 |
| ATP1A2 | 1.807814153 | 0.656836255 | -1.460640708 | 6.7061E-24 | 2.65333E-22 |
| SLAMF9 | 2.209764815 | 4.686981474 | 1.084766261 | 5.57249E-13 | 5.33916E-12 |
| LOXL2 | 17.98038862 | 44.61478672 | 1.31109774 | 1.78491E-20 | 4.91669E-19 |
| AKR1B15 | 1.478866667 | 6.390514807 | 2.11144017 | 2.67724E-07 | 1.07839E-06 |
| SNTN | 6.638845899 | 1.862489509 | -1.833700166 | 2.52037E-15 | 3.48232E-14 |
| MIR3189 | 27.92460053 | 10.85945418 | -1.36258505 | 3.66428E-12 | 3.1133E-11 |
| NIPAL4 | 0.382788492 | 1.688033201 | 2.140723916 | 1.42309E-08 | 6.98859E-08 |
| AL133466.1 | 3.39176918 | 1.131795219 | -1.583425045 | 2.54317E-20 | 6.82533E-19 |
| SLC10A2 | 4.526606085 | 0.103457105 | -5.451325135 | 1.28789E-05 | 3.85105E-05 |
| ERN2 | 23.42377262 | 10.54426952 | -1.151514302 | 1.00014E-07 | 4.33122E-07 |
| KIF2C | 9.216652513 | 32.71954708 | 1.827838014 | 9.84984E-55 | 7.45547E-52 |
| FAM171A2 | 3.862781085 | 8.35580093 | 1.113138204 | 1.39909E-06 | 4.97281E-06 |
| CDC25A | 2.446974074 | 6.870344622 | 1.489383651 | 6.0504E-45 | 1.06395E-42 |
| APOD | 137.2405464 | 65.09423201 | -1.076105158 | 1.14483E-09 | 6.72188E-09 |
| COL12A1 | 27.64547738 | 56.3881257 | 1.028347905 | 1.033E-10 | 7.02482E-10 |
| RAD51AP1 | 6.886106085 | 20.5390093 | 1.576606283 | 1.2042E-48 | 3.08293E-46 |
| LANCL1-AS1 | 1.447354894 | 0.719064011 | -1.009226608 | 7.43135E-06 | 2.32768E-05 |
| SPINK14 | 1.622645238 | 0.761983134 | -1.090516645 | 4.60858E-06 | 1.49183E-05 |
| ARTN | 1.935259127 | 4.870207902 | 1.331456607 | 2.81969E-09 | 1.55244E-08 |
| SEMA3A | 5.393578571 | 13.50564562 | 1.324247902 | 2.51844E-13 | 2.53724E-12 |
| CKS1BP3 | 0.660494048 | 1.429532138 | 1.113925589 | 2.28601E-07 | 9.32236E-07 |
| DMBT1 | 117.0660278 | 33.07412377 | -1.82354763 | 9.60047E-12 | 7.69144E-11 |
| PACRG | 2.621292196 | 0.992377092 | -1.401317843 | 5.22001E-08 | 2.35245E-07 |
| SERPINA6 | 1.838760847 | 0.551449203 | -1.737433949 | 0.000408799 | 0.00093115 |
| SORCS2 | 17.47654815 | 8.260337185 | -1.081147683 | 5.211E-14 | 5.81901E-13 |
| H19 | 52.52165066 | 192.8549504 | 1.876532016 | 0.015360594 | 0.024884848 |
| TMEM213 | 6.428222354 | 2.228799734 | -1.528152842 | 1.23429E-10 | 8.30977E-10 |
| CAPSL | 10.78124881 | 3.011560093 | -1.839941345 | 6.66631E-06 | 2.10395E-05 |
| MIR3677 | 7.573327249 | 3.667021912 | -1.046318383 | 3.13931E-09 | 1.71971E-08 |
| CD1A | 27.52873558 | 10.76360505 | -1.354776988 | 5.04514E-08 | 2.27601E-07 |
| DUOXA1 | 14.95113175 | 6.210148606 | -1.267554998 | 1.56609E-13 | 1.6316E-12 |
| S100A12 | 2.679022884 | 6.900791833 | 1.365055009 | 0.012597805 | 0.020859785 |
| AC015722.2 | 2.30555291 | 1.012357902 | -1.187393354 | 0.002220323 | 0.004359756 |
| CCL26 | 1.488800926 | 3.907483599 | 1.39208896 | 2.69154E-17 | 4.8406E-16 |
| LINC01214 | 0.567352646 | 3.257733201 | 2.521550811 | 1.33963E-12 | 1.21784E-11 |
| TNFRSF13B | 1.996353704 | 0.852431541 | -1.227711473 | 2.34293E-12 | 2.05585E-11 |
| CENPO | 3.851830423 | 7.725366534 | 1.004059194 | 1.07728E-39 | 1.29341E-37 |
| TMPO-AS1 | 1.64058836 | 3.298702855 | 1.007685529 | 1.46038E-25 | 7.0038E-24 |
| WDR38 | 11.66797222 | 3.173214542 | -1.878536886 | 3.60596E-05 | 9.91882E-05 |
| C12orf56 | 0.973944444 | 2.020242364 | 1.052616994 | 1.54863E-06 | 5.46635E-06 |
| HBQ1 | 0.500224206 | 1.910132603 | 1.933026017 | 3.77501E-06 | 1.24115E-05 |
| TNNT1 | 18.87916587 | 56.40784728 | 1.579100855 | 2.09054E-14 | 2.49446E-13 |
| IRX6 | 8.12683955 | 3.123951726 | -1.379322251 | 1.64755E-08 | 8.0051E-08 |
| NEFH | 4.307757672 | 11.33683254 | 1.396008614 | 0.004796182 | 0.008766061 |
| AC106738.2 | 1.558946561 | 0.518536919 | -1.588052859 | 5.77248E-15 | 7.53322E-14 |
| KIAA0319 | 1.91453254 | 5.95947178 | 1.638192282 | 6.05377E-06 | 1.92388E-05 |
| AC005077.4 | 2.237515344 | 6.381560624 | 1.512011705 | 3.28585E-18 | 6.76962E-17 |
| ERVMER34-1 | 2.220900132 | 7.21750571 | 1.700355824 | 3.25728E-11 | 2.39165E-10 |
| MET | 88.82103545 | 193.4147214 | 1.122724312 | 3.1265E-05 | 8.69059E-05 |
| IGHD | 250.8507173 | 114.1779363 | -1.135545172 | 2.84089E-09 | 1.56263E-08 |
| CCDC173 | 2.923930026 | 1.427307437 | -1.034612666 | 3.58431E-05 | 9.86393E-05 |
| AGTR2 | 13.48353056 | 4.320902457 | -1.641793736 | 5.69767E-05 | 0.00015211 |
| HAPLN1 | 0.942481085 | 2.152718792 | 1.191624304 | 0.001838043 | 0.003667568 |
| HOXD10 | 0.600188757 | 1.74625405 | 1.540775264 | 9.81509E-05 | 0.000250875 |
| ADGRG3 | 1.235696296 | 2.680703718 | 1.117287568 | 2.76559E-13 | 2.76862E-12 |
| SERPIND1 | 37.35739669 | 9.936196414 | -1.910628325 | 6.65095E-06 | 2.09986E-05 |
| AL033397.2 | 0.644957011 | 2.538475033 | 1.976687164 | 9.88479E-11 | 6.74047E-10 |
| CA3 | 4.562183995 | 2.179189376 | -1.065933057 | 1.16512E-18 | 2.51656E-17 |
| PLK1 | 7.416709788 | 23.47015438 | 1.661976108 | 7.20894E-55 | 5.70457E-52 |
| AF127577.3 | 0.920937963 | 2.041646614 | 1.148557293 | 0.003107848 | 0.005915003 |
| DNAAF6 | 1.819770238 | 0.49153247 | -1.888397681 | 1.3693E-05 | 4.06448E-05 |
| LINC01597 | 4.461239683 | 1.999011222 | -1.158158089 | 0.003878197 | 0.007229419 |
| AC002563.1 | 2.985899868 | 0.851687716 | -1.809769339 | 2.35172E-09 | 1.31222E-08 |
| FOSL1 | 9.129471958 | 39.67945983 | 2.119789063 | 2.3163E-19 | 5.4419E-18 |
| GFRA1 | 2.755555952 | 1.261293758 | -1.1274391 | 1.22866E-26 | 6.58144E-25 |
| DLGAP5 | 6.83517619 | 26.51661255 | 1.955846054 | 1.25462E-59 | 8.89701E-56 |
| FAM83B | 0.821718386 | 2.65302178 | 1.690920566 | 1.45004E-07 | 6.09166E-07 |
| SFTA1P | 34.75874497 | 9.180658898 | -1.920706387 | 3.07537E-25 | 1.43153E-23 |
| PBK | 6.956266667 | 24.88586375 | 1.838941317 | 4.31352E-46 | 8.34379E-44 |
| CDK6 | 6.227817196 | 13.23224382 | 1.087259221 | 2.55321E-13 | 2.57078E-12 |
| LINC01605 | 0.685305688 | 1.556953386 | 1.183906186 | 0.000554022 | 0.001232111 |
| PI16 | 2.447229894 | 0.909147809 | -1.428562864 | 2.54639E-11 | 1.89642E-10 |
| C1orf194 | 11.55155741 | 3.354375432 | -1.783971298 | 1.30977E-08 | 6.46675E-08 |
| HOXD13 | 0.316822487 | 2.635716799 | 3.056448724 | 6.8033E-06 | 2.14252E-05 |
| MAGEC1 | 0.288492857 | 4.199035657 | 3.863450535 | 1.25995E-09 | 7.33594E-09 |
| AC003092.1 | 0.326068519 | 3.307543825 | 3.34251321 | 2.74896E-07 | 1.10549E-06 |
| PNCK | 0.870403175 | 1.816348473 | 1.061285292 | 2.83996E-07 | 1.13857E-06 |
| DLC1 | 29.60575251 | 13.44384575 | -1.138931629 | 1.01482E-19 | 2.49887E-18 |
| CYP2A6 | 14.71176362 | 1.845057039 | -2.995232883 | 1.76989E-13 | 1.83296E-12 |
| CENPE | 2.058379365 | 7.026135724 | 1.771222546 | 2.36904E-53 | 1.17836E-50 |
| GGTLC1 | 72.36283849 | 20.03948386 | -1.852403649 | 1.89707E-28 | 1.20533E-26 |
| FLNC | 5.331125794 | 16.71451355 | 1.648589238 | 1.18072E-08 | 5.883E-08 |
| KIF1A | 3.341293122 | 12.56439469 | 1.910862712 | 0.031134681 | 0.046851384 |
| HPDL | 2.254632143 | 5.816502855 | 1.367259932 | 8.17229E-17 | 1.38666E-15 |
| H3C2 | 1.547437037 | 5.728332603 | 1.888234552 | 3.07203E-19 | 7.09296E-18 |
| UGT2B15 | 8.600972487 | 3.597852988 | -1.257363553 | 0.00367357 | 0.006885571 |
| VWA3B | 2.914106085 | 1.000598473 | -1.542190243 | 1.53738E-05 | 4.52098E-05 |
| ALB | 184.2781122 | 4.902717397 | -5.232159309 | 0.017066264 | 0.027370482 |
| IL12RB2 | 1.094959524 | 2.87180591 | 1.391080708 | 2.42887E-14 | 2.86285E-13 |
| SPINK5 | 31.00269921 | 12.9829923 | -1.255770896 | 9.17929E-10 | 5.45959E-09 |
| SLC7A10 | 5.023858466 | 2.370611487 | -1.083536577 | 9.39204E-08 | 4.08192E-07 |
| SCUBE2 | 11.27102407 | 5.340874701 | -1.07747066 | 7.11551E-14 | 7.79081E-13 |
| ORM1 | 63.73883862 | 25.15612238 | -1.341263173 | 1.33419E-08 | 6.57427E-08 |
| SYT1 | 2.722399471 | 8.414951328 | 1.628076151 | 2.9614E-13 | 2.95275E-12 |
| HASPIN | 1.699489153 | 4.46006162 | 1.391962488 | 3.15715E-42 | 4.39702E-40 |
| C8orf34-AS1 | 7.429200265 | 2.90710996 | -1.353621275 | 1.92555E-18 | 4.06819E-17 |
| C20orf85 | 55.97755622 | 13.0229326 | -2.103794143 | 1.49245E-09 | 8.58345E-09 |
| RRM2 | 9.351632011 | 33.25105153 | 1.830109899 | 1.93124E-57 | 4.80299E-54 |
| SYNDIG1L | 1.708490873 | 0.641193692 | -1.413890403 | 9.93719E-11 | 6.77091E-10 |
| HHATL | 2.244982011 | 0.497871248 | -2.172859276 | 1.49576E-05 | 4.40679E-05 |
| AL513548.4 | 3.321778307 | 1.069322311 | -1.635259022 | 3.55469E-05 | 9.7917E-05 |
| DBF4 | 6.614433333 | 14.49344157 | 1.131710744 | 9.96015E-49 | 2.61152E-46 |
| AL049767.1 | 4.425472354 | 1.111604183 | -1.993188281 | 1.10681E-05 | 3.36096E-05 |
| IGLV1-36 | 147.0445852 | 54.25342311 | -1.438467587 | 0.000690946 | 0.001506599 |
| PTPRT | 1.427124206 | 0.650947344 | -1.13249815 | 5.00556E-09 | 2.64066E-08 |
| AL663070.1 | 7.158285185 | 3.077802258 | -1.217713477 | 2.36808E-10 | 1.52689E-09 |
| HSD17B13 | 4.886804497 | 2.386193227 | -1.034180516 | 1.41441E-17 | 2.68229E-16 |
| CDC6 | 7.236902778 | 22.52945133 | 1.638367884 | 1.23239E-49 | 3.63638E-47 |
| CD1C | 11.45786534 | 4.960318194 | -1.207833714 | 1.43899E-15 | 2.04502E-14 |
| HJURP | 6.275256614 | 23.22238984 | 1.887770088 | 3.14061E-56 | 4.55624E-53 |
| KYNU | 3.948438228 | 12.23773074 | 1.631982037 | 5.84378E-07 | 2.22128E-06 |
| CD1E | 9.198642196 | 3.659609296 | -1.329731289 | 8.97059E-17 | 1.50307E-15 |
| AVPR1A | 1.502833598 | 3.343538048 | 1.15369026 | 0.003042935 | 0.005803514 |
| DUOX1 | 24.34150701 | 8.73650405 | -1.47829049 | 6.05494E-14 | 6.69698E-13 |
| ORC6 | 3.701900132 | 8.821576826 | 1.25277058 | 7.50896E-39 | 8.77339E-37 |
| SYT13 | 5.654930688 | 16.6216176 | 1.555479544 | 0.001685791 | 0.003391648 |
| AC007686.1 | 0.722298413 | 1.819580412 | 1.332938904 | 0.000136198 | 0.000339239 |
| AADAC | 13.47318201 | 6.010042231 | -1.164643584 | 4.7439E-06 | 1.53279E-05 |
| MS4A8 | 16.83312196 | 6.404835392 | -1.394069373 | 7.77663E-10 | 4.66839E-09 |
| AC092071.1 | 11.68421812 | 4.033633931 | -1.534409131 | 9.77353E-20 | 2.41344E-18 |
| BRIP1 | 1.620967857 | 3.95644004 | 1.28734741 | 2.68579E-38 | 3.01658E-36 |
| CFAP221 | 8.388512302 | 3.2593583 | -1.363827016 | 4.83613E-26 | 2.44745E-24 |
| POLR3G | 2.453297619 | 5.060352722 | 1.044515685 | 1.09925E-20 | 3.09659E-19 |
| MMP12 | 36.23455013 | 99.80410166 | 1.461733125 | 2.50465E-19 | 5.86068E-18 |
| TROAP | 5.598887302 | 16.80882311 | 1.58600667 | 6.82655E-42 | 9.21267E-40 |
| CA2 | 63.30653995 | 30.92094037 | -1.033770351 | 0.029747056 | 0.044973209 |
| FEN1 | 32.39728439 | 69.07438599 | 1.092277944 | 3.97072E-43 | 5.80893E-41 |
| LYPD6 | 1.003924074 | 2.414827291 | 1.266269847 | 1.66098E-07 | 6.90161E-07 |
| RN7SL3 | 30.46276733 | 98.26180538 | 1.68958374 | 1.85972E-08 | 8.95603E-08 |
| KCNK17 | 5.944271561 | 2.119309495 | -1.487905738 | 2.04469E-12 | 1.81427E-11 |
| KIF15 | 2.813483862 | 8.402012351 | 1.578377218 | 1.40401E-48 | 3.52349E-46 |
| H2BC11 | 4.913615873 | 10.23608187 | 1.058806609 | 6.87526E-12 | 5.64849E-11 |
| AC008268.1 | 15.57688519 | 4.478706175 | -1.798252849 | 1.17202E-16 | 1.92306E-15 |
| AL033397.1 | 3.182805159 | 6.728436587 | 1.079972478 | 7.46017E-09 | 3.82771E-08 |
| MYBL2 | 30.1646287 | 103.9826707 | 1.785413385 | 6.92084E-45 | 1.20485E-42 |
| MAMDC2 | 11.41192831 | 4.932926826 | -1.210026797 | 1.64551E-15 | 2.32145E-14 |
| RN7SKP51 | 4.524789683 | 2.216123307 | -1.029812579 | 5.64301E-15 | 7.3864E-14 |
| TCTE1 | 1.693423545 | 0.475722576 | -1.831750455 | 1.87356E-06 | 6.49998E-06 |
| IGHA2 | 1002.224404 | 472.1574776 | -1.08586555 | 7.69563E-07 | 2.87435E-06 |
| OGDHL | 1.172767725 | 3.529088911 | 1.589378472 | 0.001121283 | 0.002343778 |
| RHOF | 0.703083201 | 1.571123639 | 1.160029388 | 1.75508E-15 | 2.47003E-14 |
| AC009244.1 | 5.435515079 | 2.310238114 | -1.234375198 | 1.62334E-17 | 3.02253E-16 |
| PCDHB8 | 1.636641402 | 3.83753506 | 1.229441678 | 3.83748E-10 | 2.39537E-09 |
| FCRL1 | 2.090460582 | 0.974639575 | -1.100880131 | 4.5958E-08 | 2.09121E-07 |
| BCL2L10 | 1.136330026 | 3.074304183 | 1.435878017 | 1.78952E-05 | 5.19922E-05 |
| AC007240.1 | 0.70979127 | 1.696808699 | 1.257357187 | 4.03981E-11 | 2.9255E-10 |
| CCNE1 | 6.727652249 | 19.98330916 | 1.570620468 | 2.96672E-34 | 2.66225E-32 |
| LRRK2 | 37.1706504 | 15.43244475 | -1.268197305 | 7.40369E-14 | 8.08093E-13 |
| ARHGAP11A | 3.496596164 | 11.12814768 | 1.670190382 | 2.83826E-54 | 1.90043E-51 |
| VEGFD | 11.52585238 | 3.589284794 | -1.683105143 | 1.32237E-19 | 3.19301E-18 |
| LYPD5 | 1.844818783 | 4.272575631 | 1.211626923 | 4.06978E-09 | 2.1854E-08 |
| ORC1 | 4.086013492 | 12.76492211 | 1.643418862 | 1.99801E-50 | 6.32426E-48 |
| RSPO2 | 1.823081481 | 0.624254316 | -1.546173247 | 8.07436E-21 | 2.31195E-19 |
| GREB1 | 4.229784392 | 1.985493559 | -1.091086445 | 0.000931895 | 0.001981842 |
| RPL26P30 | 8.490800661 | 2.287397742 | -1.892193353 | 1.19176E-16 | 1.94995E-15 |
| SCTR-AS1 | 3.205689418 | 1.458566135 | -1.136083854 | 5.02014E-15 | 6.61587E-14 |
| TXNRD1 | 98.63376574 | 331.4315503 | 1.748557424 | 4.98715E-21 | 1.46658E-19 |
| GPC6 | 5.705526323 | 13.83639057 | 1.27803576 | 9.76988E-17 | 1.63228E-15 |
| C1orf189 | 5.106933995 | 1.590606574 | -1.682880374 | 1.93154E-06 | 6.67718E-06 |
| MBL1P | 3.954145106 | 1.753139309 | -1.173425174 | 3.09405E-14 | 3.58379E-13 |
| EPYC | 1.614937434 | 3.288806441 | 1.02608583 | 7.15634E-06 | 2.24761E-05 |
| G6PD | 98.11796336 | 222.343147 | 1.180198746 | 8.76772E-13 | 8.17116E-12 |
| PLOD2 | 29.76568796 | 80.14078699 | 1.428886438 | 3.61776E-27 | 2.03166E-25 |
| HILPDA | 22.8378336 | 52.98115518 | 1.214053497 | 1.33508E-24 | 5.75309E-23 |
| SKA3 | 3.985163492 | 12.78674416 | 1.681938145 | 5.22828E-54 | 3.03397E-51 |
| NME9 | 1.52607209 | 0.710049602 | -1.1038314 | 8.72058E-08 | 3.80304E-07 |
| WDHD1 | 5.30958955 | 13.04527709 | 1.296855343 | 2.96438E-42 | 4.16185E-40 |
| ARHGEF39 | 2.079777116 | 4.381817795 | 1.075100568 | 6.20689E-35 | 5.80945E-33 |
| L1CAM | 0.895547619 | 4.890492231 | 2.449137629 | 0.00112012 | 0.002341801 |
| C4BPA | 504.2743083 | 183.874801 | -1.455484945 | 7.54302E-18 | 1.48213E-16 |
| KIF23 | 4.543603439 | 15.2511332 | 1.747007614 | 1.61768E-57 | 4.69371E-54 |
| LMNB1 | 28.25283585 | 63.22805724 | 1.162169208 | 8.16243E-35 | 7.5585E-33 |
| CEACAM4 | 5.633229365 | 2.810726228 | -1.003019276 | 7.28439E-05 | 0.000190612 |
| ECRG4 | 7.592090608 | 1.889471448 | -2.006514493 | 2.02807E-20 | 5.53397E-19 |
| MAGEA10 | 2.499008069 | 7.081567663 | 1.502713209 | 5.87915E-05 | 0.000156546 |
| RGS22 | 1.612597222 | 0.6233751 | -1.371213709 | 1.95939E-10 | 1.27375E-09 |
| NFE4 | 1.204377513 | 11.45867869 | 3.250081113 | 5.60903E-13 | 5.37115E-12 |
| GPR19 | 0.889494577 | 2.234038313 | 1.328596214 | 1.40123E-29 | 9.79676E-28 |
| ERICH3 | 2.873231481 | 0.855851793 | -1.747241335 | 2.57273E-05 | 7.26969E-05 |
| CFAP77 | 3.837482275 | 1.071440637 | -1.840608166 | 1.71909E-06 | 6.00232E-06 |
| MUCL3 | 50.96376931 | 16.16010345 | -1.65703555 | 2.07288E-13 | 2.12025E-12 |
| AC102953.2 | 2.443969444 | 5.424468393 | 1.15025551 | 5.28423E-16 | 8.02033E-15 |
| AP003555.1 | 0.623469577 | 1.908852855 | 1.614314828 | 0.017636383 | 0.028190093 |
| HMGA2 | 1.118614021 | 7.588943758 | 2.762186784 | 3.35411E-09 | 1.82703E-08 |
| EME1 | 1.919499206 | 4.682581408 | 1.286574113 | 4.29949E-38 | 4.79807E-36 |
| CDK1 | 14.80823955 | 46.2016755 | 1.641545033 | 2.04036E-55 | 2.22004E-52 |
| B4GALNT1 | 1.283523016 | 3.111529615 | 1.277514811 | 1.84327E-08 | 8.88658E-08 |
| ASPM | 3.216406349 | 11.06771042 | 1.782835215 | 7.83054E-52 | 2.96352E-49 |
| SV2A | 3.743520767 | 10.05623114 | 1.425622053 | 6.98986E-10 | 4.22083E-09 |
| TNS4 | 14.57135556 | 37.72016202 | 1.372200777 | 0.000129759 | 0.000324473 |
| FKBP9P1 | 1.798614418 | 3.840031873 | 1.094232347 | 0.004823769 | 0.008809084 |
| SLIT3 | 8.614842593 | 4.209168592 | -1.03328914 | 1.89894E-11 | 1.43984E-10 |
| SPATA18 | 12.51371402 | 4.595810093 | -1.44511895 | 1.53967E-17 | 2.89462E-16 |
| GDA | 0.878191799 | 2.110167995 | 1.264749892 | 0.013932543 | 0.02278336 |
| ITGA9 | 18.06143201 | 8.963434595 | -1.010788729 | 3.86033E-17 | 6.77464E-16 |
| TMEM163 | 25.78344524 | 11.81692185 | -1.125590771 | 1.75098E-19 | 4.17001E-18 |
| HHIPL2 | 5.888664021 | 13.26501023 | 1.17161352 | 6.42345E-11 | 4.50547E-10 |
| TEKT2 | 9.838145106 | 3.939563015 | -1.320350722 | 4.11647E-07 | 1.60789E-06 |
| GJB2 | 30.68288624 | 74.1307998 | 1.272638878 | 1.0133E-14 | 1.26727E-13 |
| AC113346.1 | 0.732495238 | 1.621759097 | 1.146668246 | 8.75815E-12 | 7.05897E-11 |
| CDIPTOSP | 1.712362169 | 3.560413413 | 1.0560569 | 9.19067E-05 | 0.000236249 |
| LINC00973 | 1.290713889 | 10.08712543 | 2.966273962 | 7.65843E-08 | 3.37192E-07 |
| EFHB | 2.187458069 | 0.927081873 | -1.238486705 | 3.29729E-08 | 1.5336E-07 |
| SRP14P4 | 5.404952381 | 2.525858167 | -1.097508277 | 1.60157E-19 | 3.8299E-18 |
| CGA | 8.904237302 | 39.02191846 | 2.131720761 | 0.006668233 | 0.011813094 |
| AC002480.1 | 0.857795238 | 1.754014542 | 1.031955496 | 0.000565273 | 0.001254573 |
| RPSAP52 | 0.482521032 | 1.543770916 | 1.677794952 | 1.01506E-06 | 3.70619E-06 |
| AL133304.2 | 2.832746164 | 0.892828088 | -1.665747012 | 9.33112E-09 | 4.71951E-08 |
| AC068587.2 | 5.099384788 | 1.889403187 | -1.432392608 | 1.41971E-10 | 9.44069E-10 |
| CD109 | 6.544778704 | 21.49402098 | 1.715519083 | 6.98296E-23 | 2.5117E-21 |
| ECT2L | 2.258046561 | 0.722811952 | -1.643382967 | 8.43016E-05 | 0.000218103 |
| CKAP2L | 2.701767196 | 9.762514475 | 1.853349416 | 7.21493E-59 | 3.14012E-55 |
| STC1 | 15.21222116 | 43.70244157 | 1.522483063 | 1.53133E-21 | 4.76905E-20 |
| MYOSLID | 1.188109259 | 3.245517065 | 1.449780829 | 1.65556E-07 | 6.8869E-07 |
| H2BC17 | 1.319278704 | 4.181815538 | 1.664380053 | 9.91251E-18 | 1.92382E-16 |
| AP003327.2 | 0.637248677 | 1.732693891 | 1.443088422 | 6.11837E-06 | 1.94193E-05 |
| LINC01116 | 1.573633598 | 5.544163944 | 1.816870254 | 7.05859E-11 | 4.91925E-10 |
| SLC13A2 | 9.271003175 | 3.090015737 | -1.585111269 | 4.03694E-10 | 2.51266E-09 |
| PRSS3 | 3.170201984 | 8.954823904 | 1.4980903 | 4.6477E-07 | 1.80124E-06 |
| ROPN1L | 6.818669048 | 3.028933931 | -1.170680054 | 0.001501352 | 0.003058036 |
| RUNDC3A | 0.88466455 | 2.504489641 | 1.501314226 | 4.82972E-06 | 1.55965E-05 |
| SLC47A1 | 11.22546627 | 5.113757304 | -1.134319773 | 5.83244E-15 | 7.59439E-14 |
| FANCB | 0.906203968 | 2.092924502 | 1.207612557 | 1.94999E-36 | 1.96228E-34 |
| CDCA5 | 9.671860847 | 32.59016149 | 1.752571108 | 3.18313E-54 | 2.05241E-51 |
| TTLL10 | 2.001327249 | 0.596991567 | -1.745174632 | 3.09071E-06 | 1.03117E-05 |
| SFTPC | 1772.760403 | 361.9409327 | -2.292171383 | 2.15547E-12 | 1.89998E-11 |
| PIF1 | 1.935215873 | 4.304203519 | 1.153251788 | 4.14827E-27 | 2.29991E-25 |
| HOXA1 | 1.20713664 | 4.278672908 | 1.825574404 | 1.60706E-08 | 7.82587E-08 |
| RPL10P19 | 3.908931878 | 1.841065803 | -1.08623325 | 1.56433E-17 | 2.92833E-16 |
| NCAPG | 4.01963545 | 15.76440784 | 1.971534407 | 2.46949E-57 | 5.37391E-54 |
| AC079384.1 | 5.948809127 | 2.783000266 | -1.095959842 | 0.000485982 | 0.001093083 |
| TMEM145 | 0.863037698 | 1.905947145 | 1.143012627 | 1.99943E-07 | 8.2134E-07 |
| DEPDC1 | 2.334353571 | 9.273677291 | 1.99011843 | 4.96003E-56 | 6.64224E-53 |
| FLACC1 | 1.436737037 | 0.601766733 | -1.255519774 | 7.95651E-08 | 3.48995E-07 |
| TICRR | 1.118528042 | 3.394476361 | 1.601587612 | 2.32968E-46 | 4.55702E-44 |
| AC005291.2 | 0.466869312 | 1.819796016 | 1.962686078 | 0.011494379 | 0.019222445 |
| AUNIP | 2.060749471 | 6.065977424 | 1.557571004 | 3.13651E-44 | 5.05587E-42 |
| SCNN1B | 81.84864299 | 36.99333293 | -1.145693211 | 3.09247E-24 | 1.27274E-22 |
| MYBL1 | 2.304083598 | 5.399779814 | 1.228707518 | 1.50597E-20 | 4.16149E-19 |
| C18orf54 | 1.70295754 | 3.407874303 | 1.000829659 | 1.45804E-28 | 9.47125E-27 |
| FGF12 | 0.838361508 | 2.873784728 | 1.777307611 | 5.06972E-05 | 0.000136475 |
| DNAI3 | 2.281737037 | 0.931838114 | -1.291981289 | 2.82973E-05 | 7.94053E-05 |
| TPX2 | 23.75100489 | 90.30293811 | 1.926784374 | 2.77887E-55 | 2.68763E-52 |
| AC006238.1 | 1.990578571 | 0.991415936 | -1.005625464 | 8.71446E-13 | 8.12587E-12 |
| IGF2BP2 | 11.09809167 | 26.47660817 | 1.254406691 | 1.1525E-16 | 1.89281E-15 |
| MCM10 | 2.604045503 | 9.610457968 | 1.883850523 | 3.33103E-53 | 1.56729E-50 |
| PLEKHH1 | 1.704511508 | 3.579973904 | 1.070590731 | 1.2174E-08 | 6.05362E-08 |
| SLPI | 3399.343378 | 1038.771769 | -1.710377389 | 1.83161E-12 | 1.63604E-11 |
| STEAP1 | 31.38635119 | 72.47888612 | 1.207423465 | 8.46951E-21 | 2.41714E-19 |
| GAL | 2.362342857 | 14.83193114 | 2.650416182 | 8.5971E-17 | 1.44746E-15 |
| KIR2DL4 | 0.92639127 | 1.953516467 | 1.076379854 | 3.67535E-09 | 1.99204E-08 |
| PIGR | 1135.382718 | 312.5419745 | -1.861056825 | 4.12564E-23 | 1.51526E-21 |
| UCHL1 | 58.88516019 | 166.0613042 | 1.495739926 | 4.77141E-10 | 2.94558E-09 |
| LINC02323 | 0.611358995 | 2.153614276 | 1.816668183 | 9.0002E-14 | 9.71385E-13 |
| CST5 | 3.02845119 | 1.030024701 | -1.555901224 | 5.76465E-21 | 1.67976E-19 |
| LUCAT1 | 1.694645106 | 5.158941567 | 1.606091931 | 2.79756E-22 | 9.42025E-21 |
| AK4 | 7.86804127 | 16.86193632 | 1.099693786 | 2.04438E-18 | 4.3088E-17 |
| SLC22A31 | 206.797929 | 95.67443645 | -1.112016334 | 6.85013E-18 | 1.35362E-16 |
| FRMD5 | 2.556526323 | 5.448303386 | 1.091622161 | 3.43103E-17 | 6.09498E-16 |
| AL357093.2 | 2.177645767 | 0.845788911 | -1.364399742 | 1.53328E-07 | 6.41191E-07 |
| STMND1 | 3.142741005 | 1.30474429 | -1.268256296 | 2.42407E-09 | 1.34869E-08 |
| KRT81 | 21.18601005 | 66.25825498 | 1.644988297 | 0.000314595 | 0.00073229 |
| FDCSP | 42.84769524 | 15.89656301 | -1.430502729 | 3.86728E-09 | 2.08826E-08 |
| AC131206.1 | 1.772909259 | 4.135025299 | 1.221777458 | 0.006553015 | 0.011620804 |
| ECT2 | 18.57134921 | 49.15143546 | 1.40415492 | 5.24844E-48 | 1.23473E-45 |
| POLQ | 1.207209921 | 3.67235405 | 1.605028588 | 1.17954E-43 | 1.84997E-41 |
| SP8 | 1.05066455 | 3.566082537 | 1.763037966 | 3.1796E-08 | 1.48123E-07 |
| CDH3 | 45.61661085 | 91.89918254 | 1.010492765 | 5.4282E-10 | 3.32979E-09 |
| SERPINA5 | 2.288249074 | 4.631101793 | 1.017111371 | 0.023146942 | 0.036021464 |
| DUSP13 | 1.52002328 | 5.614144754 | 1.884972843 | 1.81711E-11 | 1.38443E-10 |
| AKR1B10 | 80.30148333 | 238.6270479 | 1.571259037 | 3.9042E-06 | 1.28097E-05 |
| H4C4 | 2.129997751 | 5.939958367 | 1.479600912 | 0.001095069 | 0.002293558 |
| AC011294.1 | 1.165762169 | 2.460598606 | 1.077735842 | 0.000460835 | 0.001040554 |
| IL1A | 1.306060185 | 5.899784529 | 2.175440886 | 5.84373E-07 | 2.22128E-06 |
| IGF2BP1 | 1.152893122 | 6.383369256 | 2.46905933 | 8.49246E-10 | 5.07884E-09 |
| AC002401.4 | 2.927560185 | 6.17579741 | 1.076926597 | 0.011179004 | 0.018756291 |
| AGER | 185.8788087 | 55.79359369 | -1.73619092 | 8.19894E-13 | 7.67395E-12 |
| TTC29 | 2.008585582 | 0.614823772 | -1.707935082 | 3.90123E-06 | 1.28048E-05 |
| CDC25C | 2.444663095 | 7.051684263 | 1.528332222 | 7.05148E-46 | 1.31999E-43 |
| NGEF | 5.367728571 | 11.05805923 | 1.042714578 | 2.37265E-07 | 9.64629E-07 |
| SCN4B | 5.159773016 | 2.218474104 | -1.217739889 | 2.21842E-22 | 7.57263E-21 |
| FCGBP | 31.20158267 | 13.5767668 | -1.200479256 | 1.80541E-11 | 1.37732E-10 |
| IQGAP3 | 8.990957011 | 24.16475199 | 1.426357597 | 2.53674E-45 | 4.50633E-43 |
| APOA1 | 4.46264828 | 1.711421381 | -1.382705087 | 0.000123123 | 0.000309241 |
| C1orf116 | 207.5955534 | 92.56757344 | -1.165196734 | 2.9607E-19 | 6.84499E-18 |
| BUB1 | 6.412939418 | 21.24212789 | 1.727870609 | 1.1872E-55 | 1.47628E-52 |
| ALOX15B | 103.0022705 | 48.95748386 | -1.073074821 | 4.46642E-12 | 3.75814E-11 |
| PFKFB4 | 5.213175132 | 11.43748685 | 1.133535853 | 3.7231E-21 | 1.1023E-19 |
| LRRC36 | 5.389903175 | 1.160917862 | -2.214993455 | 8.19936E-19 | 1.80003E-17 |
| ABCC2 | 1.3786 | 19.06730365 | 3.789825018 | 1.36692E-07 | 5.7731E-07 |
| CCDC190 | 1.843272884 | 0.6320583 | -1.544140126 | 1.62373E-05 | 4.75643E-05 |
| PYGL | 28.40301177 | 62.07014177 | 1.127855524 | 8.17268E-26 | 4.01916E-24 |
| ARHGEF4 | 1.945643651 | 4.551808699 | 1.226192425 | 6.41766E-10 | 3.90511E-09 |
| AC073210.3 | 1.630965344 | 3.807529017 | 1.223128905 | 0.001813144 | 0.00362483 |
| HSF2BP | 1.142320106 | 2.310083333 | 1.01597791 | 5.24444E-19 | 1.18112E-17 |
| UHRF1 | 5.605507804 | 16.28223997 | 1.538382209 | 1.04307E-46 | 2.11149E-44 |
| KIF20A | 9.787887963 | 26.27372477 | 1.42455125 | 2.39708E-45 | 4.30215E-43 |
| PLA2G10 | 38.5397422 | 10.86468871 | -1.826700081 | 6.1562E-19 | 1.37578E-17 |
| MCM4 | 27.51856786 | 64.56066262 | 1.230249996 | 8.16793E-40 | 9.87469E-38 |
| CDC7 | 6.785275132 | 14.19223798 | 1.064622886 | 4.04374E-32 | 3.22924E-30 |
| APOBEC3B | 6.154666931 | 14.53602537 | 1.239880152 | 2.48218E-09 | 1.3775E-08 |
| PTCSC3 | 12.69951918 | 4.554023705 | -1.479560169 | 5.79599E-22 | 1.88602E-20 |
| ALDOB | 4.336133598 | 1.231029482 | -1.816543894 | 1.15913E-10 | 7.83661E-10 |
| PLK4 | 3.767464153 | 10.51038227 | 1.480149452 | 1.14777E-51 | 4.16281E-49 |
| RSPO1 | 1.66982963 | 0.410256507 | -2.025102792 | 3.81152E-12 | 3.23051E-11 |
| SFTPB | 11019.05547 | 3455.973426 | -1.672836536 | 3.79089E-27 | 2.10848E-25 |
| ASPG | 3.335328175 | 1.059592829 | -1.654318735 | 0.000482294 | 0.001085207 |
| NFIX | 58.57059101 | 27.40399841 | -1.095790046 | 9.19758E-22 | 2.93799E-20 |
| H2AC12 | 0.896683995 | 3.183633068 | 1.828002515 | 7.39634E-11 | 5.13819E-10 |
| MTHFD2 | 12.36213439 | 26.23666189 | 1.085656322 | 3.11167E-37 | 3.18653E-35 |
| S100A8 | 121.8590706 | 323.2017924 | 1.407221556 | 0.000194315 | 0.000469316 |
| H2AC4 | 0.506596032 | 1.62284927 | 1.679621327 | 1.24995E-05 | 3.75026E-05 |
| AC089983.1 | 0.695126323 | 4.609677158 | 2.729318632 | 1.34359E-17 | 2.56476E-16 |
| IGFBP1 | 4.31493955 | 11.57320651 | 1.423376384 | 1.75732E-07 | 7.2743E-07 |
| RHOV | 31.69545225 | 99.62432304 | 1.652222163 | 2.20545E-13 | 2.24399E-12 |
| SPC25 | 4.896595899 | 13.90537019 | 1.505791107 | 2.18425E-48 | 5.35572E-46 |
| RPL13AP17 | 2.666258069 | 0.619515007 | -2.105605292 | 3.85725E-16 | 5.9531E-15 |
| SGO2 | 3.270287698 | 8.545652722 | 1.38577313 | 1.00507E-48 | 2.61152E-46 |
| MFAP5 | 4.989076323 | 10.95723493 | 1.135039133 | 7.54985E-06 | 2.36055E-05 |
| CCNE2 | 2.024247222 | 4.437716268 | 1.132431932 | 7.73309E-26 | 3.84644E-24 |
| AC018629.1 | 1.895170106 | 4.779232603 | 1.334451638 | 0.001881934 | 0.003748152 |
| SLC16A11 | 5.76901045 | 2.226102988 | -1.373803538 | 6.93567E-16 | 1.03111E-14 |
| INSYN1 | 1.649241667 | 0.638383732 | -1.369307023 | 1.48867E-20 | 4.12023E-19 |
| TEKT1 | 6.925351323 | 1.762425299 | -1.974325151 | 6.17219E-08 | 2.75164E-07 |
| PRECSIT | 2.444860582 | 4.898864077 | 1.002695065 | 1.62115E-13 | 1.68293E-12 |
| CLDN18 | 107.1019347 | 51.61555458 | -1.053106742 | 1.69301E-10 | 1.11179E-09 |
| RNU6-247P | 5.071721958 | 1.675635458 | -1.597767338 | 1.75987E-06 | 6.13244E-06 |
| MEGF11 | 2.157689947 | 0.689775896 | -1.645287948 | 8.53356E-11 | 5.88592E-10 |
| VSIG2 | 85.16452196 | 32.91276129 | -1.371605485 | 7.71719E-24 | 3.03956E-22 |
| NUDT11 | 0.909344444 | 2.554877822 | 1.490355529 | 0.000430807 | 0.00097706 |
| CDA | 38.82141257 | 99.97946401 | 1.364779179 | 2.18258E-05 | 6.24634E-05 |
| ZIC2 | 1.239302249 | 3.000498871 | 1.275674303 | 1.16476E-07 | 4.98215E-07 |
| RAP1GAP | 78.23166071 | 38.86526926 | -1.00927108 | 1.1826E-16 | 1.93676E-15 |
| DNMT3B | 3.25176746 | 7.96544409 | 1.292530706 | 1.8423E-20 | 5.05079E-19 |
| TCAM1P | 0.841551852 | 2.112027623 | 1.327504633 | 0.000864875 | 0.001851298 |
| ZMYND10 | 13.04387685 | 4.857061421 | -1.42521709 | 3.68675E-08 | 1.69975E-07 |
| DNASE2B | 1.901439153 | 0.944401926 | -1.009618885 | 2.01588E-08 | 9.64664E-08 |
| AC090001.1 | 1.547500794 | 0.690884728 | -1.163423223 | 3.09619E-05 | 8.61324E-05 |
| C8orf34 | 1.448525794 | 0.608088712 | -1.252231661 | 4.04915E-20 | 1.06097E-18 |
| RDM1 | 0.892083598 | 2.264918725 | 1.344208463 | 1.31239E-25 | 6.32893E-24 |
| VWDE | 1.132982407 | 3.426263612 | 1.596510695 | 1.00505E-16 | 1.67275E-15 |
| KNL1 | 1.808514286 | 5.58009502 | 1.625484695 | 1.56068E-51 | 5.54487E-49 |
| DNAH12 | 1.500183995 | 0.512088645 | -1.55067398 | 5.39656E-07 | 2.06435E-06 |
| LRRC71 | 2.823258333 | 0.746945684 | -1.918285901 | 7.15969E-08 | 3.16353E-07 |
| ZNF367 | 3.666225661 | 8.657235392 | 1.239610799 | 4.71169E-38 | 5.22457E-36 |
| SEPTIN3 | 2.641674868 | 5.411299934 | 1.034522295 | 0.000259216 | 0.000612306 |
| ACKR1 | 28.49545952 | 12.01242344 | -1.246204822 | 7.88954E-19 | 1.7386E-17 |
| CLEC4F | 1.467371429 | 0.582686122 | -1.332443244 | 4.05277E-20 | 1.06097E-18 |
| HMGA1 | 224.1666966 | 585.9260484 | 1.38614663 | 4.43782E-34 | 3.92172E-32 |
| PIMREG | 3.805200397 | 13.00201693 | 1.772691099 | 2.59188E-48 | 6.26695E-46 |
| SLC25A47P1 | 1.711021296 | 0.648293028 | -1.400139754 | 0.006424697 | 0.011436355 |
| DNAAF1 | 4.246947884 | 1.210479814 | -1.810847384 | 1.33913E-09 | 7.76061E-09 |
| LIN28A | 0.050699206 | 2.285920186 | 5.494668058 | 0.000387723 | 0.000886042 |
| AK4P1 | 0.745227116 | 1.939306773 | 1.379788962 | 7.9704E-28 | 4.81794E-26 |
| SAPCD2 | 5.007687831 | 13.80162105 | 1.462621191 | 9.10828E-42 | 1.21043E-39 |
| FGFBP1 | 15.78097275 | 39.59282829 | 1.327052992 | 9.43576E-06 | 2.89611E-05 |
| CCDC60 | 1.88829418 | 0.582208566 | -1.697475571 | 5.53521E-14 | 6.15735E-13 |
| APOBEC4 | 1.964837566 | 0.573419788 | -1.776746454 | 2.02908E-06 | 6.98244E-06 |
| AC112721.2 | 0.603072487 | 1.851996813 | 1.618678292 | 6.12773E-12 | 5.08716E-11 |
| TGFBI | 91.88059722 | 196.0199459 | 1.093168323 | 2.37273E-09 | 1.32182E-08 |
| MEIOB | 0.652122487 | 1.874076096 | 1.522964661 | 8.19637E-06 | 2.5426E-05 |
| CBX2 | 7.328969577 | 15.6224579 | 1.091939172 | 5.16136E-13 | 4.96706E-12 |
| CFAP43 | 4.931949339 | 1.811828287 | -1.444711748 | 0.000278111 | 0.00065348 |
| CYS1 | 11.25885185 | 5.454132337 | -1.045638102 | 9.79614E-07 | 3.58581E-06 |
| CDCA3 | 3.839250661 | 11.54404602 | 1.588252295 | 1.68052E-49 | 4.87604E-47 |
| POC1A | 9.574999339 | 19.94517244 | 1.058695304 | 1.51101E-43 | 2.32788E-41 |
| MMP3 | 2.204959127 | 5.937154714 | 1.429019795 | 6.50763E-06 | 2.05858E-05 |
| MAGEA6 | 5.141036243 | 25.85670797 | 2.330407517 | 1.75113E-08 | 8.46582E-08 |
| LINC01564 | 1.46273082 | 3.290917198 | 1.169825426 | 3.80389E-14 | 4.3339E-13 |
| NR0B1 | 4.164299735 | 14.91053692 | 1.84018639 | 1.32994E-06 | 4.74736E-06 |
| SHCBP1 | 3.535320238 | 9.645702058 | 1.448045344 | 1.34375E-47 | 3.03809E-45 |
| TESMIN | 1.652858862 | 4.971882537 | 1.588828674 | 5.67371E-27 | 3.10609E-25 |
| HELLS | 3.439484392 | 7.190696016 | 1.063939113 | 4.56794E-31 | 3.48786E-29 |
| LINC01843 | 2.232588889 | 5.026934861 | 1.170961378 | 2.52018E-06 | 8.53575E-06 |
| AC023421.1 | 17.6069127 | 8.060932736 | -1.127123272 | 5.73696E-08 | 2.57078E-07 |
| LINC02313 | 0.957258598 | 2.391355113 | 1.320847766 | 0.000113039 | 0.000285807 |
| AC141557.2 | 0.929419444 | 2.138885591 | 1.202457582 | 2.21499E-11 | 1.66282E-10 |
| SLC15A1 | 1.877550794 | 5.086918991 | 1.437940182 | 0.003121928 | 0.005937905 |
| C10orf88B | 1.214541667 | 2.991906906 | 1.300653301 | 4.23737E-30 | 3.12578E-28 |
| RGS20 | 0.914884392 | 3.315651394 | 1.857630975 | 5.61709E-18 | 1.12659E-16 |
| CHIA | 15.08457381 | 2.621318792 | -2.524709213 | 1.39511E-21 | 4.38402E-20 |
| CRTAC1 | 36.8627168 | 14.3091822 | -1.36522118 | 3.49469E-12 | 2.97356E-11 |
| MGAT5B | 0.431225529 | 1.970886454 | 2.192330168 | 4.61516E-06 | 1.49342E-05 |
| TRIM9 | 0.87260172 | 1.911192032 | 1.131077522 | 2.81442E-09 | 1.55051E-08 |
| KCNF1 | 0.875768651 | 3.298419389 | 1.913153134 | 0.002491137 | 0.004837009 |
| PFKP | 33.57655992 | 90.81667211 | 1.435502745 | 2.53042E-43 | 3.79759E-41 |
| OIP5 | 1.337737566 | 3.497339243 | 1.386462625 | 1.17855E-37 | 1.25106E-35 |
| C6orf118 | 3.373834788 | 0.918112284 | -1.877646819 | 1.17503E-07 | 5.02235E-07 |
| NTSR1 | 0.291922884 | 4.085662151 | 3.806910699 | 1.17445E-05 | 3.54229E-05 |
| LINC02555 | 5.836704762 | 1.372562218 | -2.088282547 | 2.08729E-07 | 8.56011E-07 |
| AC110741.1 | 6.103350397 | 0.894147012 | -2.771017461 | 1.90604E-08 | 9.15118E-08 |
| RPS29P11 | 39.72536799 | 17.43363141 | -1.188187471 | 5.78082E-18 | 1.15809E-16 |
| ALPL | 191.2802075 | 81.39186534 | -1.232731083 | 9.98735E-15 | 1.25176E-13 |
| AL713899.1 | 1.235297487 | 3.04344668 | 1.300847574 | 1.505E-11 | 1.16514E-10 |
| AC245041.2 | 1.651396429 | 3.398331408 | 1.041140062 | 0.006384632 | 0.011374341 |
| EFCAB12 | 1.395799471 | 0.635782935 | -1.134485491 | 3.30486E-09 | 1.80132E-08 |
| CHEK1 | 5.527455556 | 16.71023154 | 1.596044299 | 1.14037E-49 | 3.42287E-47 |
| BEND6 | 1.001057804 | 2.335498871 | 1.222205466 | 1.33475E-16 | 2.16357E-15 |
| MAGEC2 | 2.281118519 | 11.12616687 | 2.286143337 | 8.13037E-12 | 6.58946E-11 |
| FIBCD1 | 1.695849206 | 3.736866401 | 1.139821094 | 0.000109584 | 0.000277854 |
| AL135999.3 | 22.99738571 | 11.24262629 | -1.032490778 | 2.49646E-18 | 5.21114E-17 |
| FCER2 | 2.411666138 | 0.844499867 | -1.513861098 | 1.36254E-10 | 9.09175E-10 |
| PAX9 | 5.355638624 | 12.7923664 | 1.256152644 | 2.07177E-06 | 7.11669E-06 |
| GPIHBP1 | 8.561102646 | 3.711551394 | -1.205774277 | 4.19509E-11 | 3.02662E-10 |
| CYP2B7P | 208.8703902 | 66.63891534 | -1.648171165 | 8.04199E-27 | 4.36147E-25 |
| IL36RN | 1.123821429 | 5.674649734 | 2.336118531 | 1.1607E-11 | 9.16399E-11 |
| CENPA | 4.972027778 | 18.36850876 | 1.885328245 | 2.11015E-54 | 1.46942E-51 |
| C16orf89 | 644.37513 | 132.8195453 | -2.278433349 | 2.19607E-35 | 2.11223E-33 |
| SGO1 | 1.439014418 | 4.640197942 | 1.689105302 | 6.51419E-53 | 2.98435E-50 |
| MIR6071 | 5.667796296 | 1.927317596 | -1.55619358 | 3.1339E-12 | 2.68759E-11 |
| COLCA1 | 9.033742593 | 4.373537517 | -1.046523137 | 1.31761E-12 | 1.19908E-11 |
| AURKA | 16.19407553 | 41.06279449 | 1.342365701 | 8.66132E-47 | 1.79506E-44 |
| SPAG6 | 8.936356481 | 3.713474568 | -1.26691704 | 7.39841E-06 | 2.31778E-05 |
| AC091057.1 | 1.341678571 | 2.720417795 | 1.019789151 | 3.24439E-26 | 1.68602E-24 |
| RIC3 | 2.087577513 | 0.818454847 | -1.350855034 | 9.51662E-15 | 1.19535E-13 |
| AC009093.3 | 2.43159127 | 0.842569256 | -1.529033563 | 1.30334E-11 | 1.02114E-10 |
| OMG | 4.718452116 | 2.241260027 | -1.074003625 | 3.748E-10 | 2.34287E-09 |
| KIF18A | 3.223615608 | 8.234804449 | 1.353054667 | 1.98738E-45 | 3.64192E-43 |
| DLL3 | 2.700684921 | 8.774238048 | 1.699948512 | 0.00091341 | 0.00194705 |
| MLXP1 | 0.674491534 | 4.817635724 | 2.83645307 | 5.55413E-09 | 2.90628E-08 |
| SLC22A3 | 49.32949616 | 20.64997118 | -1.256310784 | 4.88644E-20 | 1.26589E-18 |
| LASTR | 0.574565873 | 1.662342629 | 1.53267356 | 6.43619E-20 | 1.63097E-18 |
| ANXA8 | 1.114999074 | 2.977967131 | 1.417285319 | 0.001250616 | 0.002589129 |
| PGM5P4 | 1.788830423 | 0.746235657 | -1.261313427 | 3.952E-11 | 2.86429E-10 |
| ABCA3 | 153.3933943 | 70.81213393 | -1.115167858 | 5.30298E-13 | 5.09209E-12 |
| WIF1 | 101.4657269 | 22.55094044 | -2.169732991 | 5.88898E-15 | 7.65656E-14 |
| ERCC6L | 1.942388228 | 5.704193758 | 1.554191405 | 2.31572E-47 | 5.10309E-45 |
| AL365181.2 | 2.532109788 | 8.484660558 | 1.744516984 | 2.99332E-12 | 2.57237E-11 |
| AL136452.1 | 3.450398545 | 1.299053453 | -1.409302217 | 8.4186E-14 | 9.11439E-13 |
| CD207 | 36.3252422 | 12.74024874 | -1.511578972 | 9.6104E-14 | 1.03086E-12 |
| AC079467.1 | 6.379160847 | 2.349918991 | -1.440755632 | 2.76508E-09 | 1.52574E-08 |
| CHRDL1 | 23.94068571 | 11.2516571 | -1.089326983 | 8.3219E-17 | 1.4093E-15 |
| COCH | 1.975977249 | 6.235040305 | 1.65783255 | 1.1737E-14 | 1.45637E-13 |
| LRP4 | 3.972110053 | 8.82684429 | 1.151992153 | 0.014182799 | 0.023155618 |
| GLDC | 1.380803968 | 3.694561819 | 1.419894753 | 2.92292E-05 | 8.17433E-05 |
| ANLN | 9.853178704 | 45.60465784 | 2.210520054 | 1.31673E-66 | 2.29229E-62 |
| FAM133A | 1.148000397 | 9.082163944 | 2.983912939 | 1.80506E-05 | 5.24175E-05 |
| PLAC9 | 14.27434921 | 7.123965007 | -1.002672637 | 2.79623E-24 | 1.1618E-22 |
| ATP8B3 | 1.410636508 | 3.684505976 | 1.385124911 | 1.3984E-11 | 1.08779E-10 |
| SLC16A12 | 3.843547619 | 1.61375073 | -1.252020794 | 0.017740009 | 0.028336161 |
| AC010998.3 | 3.046355952 | 0.489738645 | -2.637000575 | 1.27894E-17 | 2.44939E-16 |
| TDRD10 | 10.58848347 | 4.243697278 | -1.319102323 | 2.61839E-21 | 7.87281E-20 |
| SLCO4A1 | 5.398345635 | 11.90062211 | 1.140447738 | 6.09586E-08 | 2.71901E-07 |
| SCN7A | 8.307162831 | 3.425231806 | -1.278154206 | 1.48014E-20 | 4.10314E-19 |
| LINC01781 | 2.213011376 | 1.023074502 | -1.113099659 | 2.0023E-06 | 6.90122E-06 |
| HSPA6 | 12.99447857 | 33.6678595 | 1.373473258 | 6.68952E-06 | 2.11089E-05 |
| H1-3 | 2.799008333 | 7.169732337 | 1.357003479 | 5.18283E-07 | 1.99003E-06 |
| CCDC17 | 9.336567857 | 3.392087981 | -1.46071872 | 0.000209247 | 0.000502245 |
| MS4A2 | 4.801163228 | 2.266987251 | -1.082607707 | 2.08288E-11 | 1.57041E-10 |
| BCAR4 | 1.403724471 | 3.415760425 | 1.282947006 | 0.028199064 | 0.042889875 |
| ANKFN1 | 1.544041402 | 0.670317463 | -1.203795012 | 2.79689E-12 | 2.42003E-11 |
| AC106045.1 | 1.865112566 | 6.685832337 | 1.841844472 | 3.92444E-10 | 2.44614E-09 |
| PKP2 | 4.918900794 | 14.87963094 | 1.59693088 | 1.80713E-16 | 2.89957E-15 |
| AL121956.7 | 3.476170238 | 1.322638579 | -1.394079849 | 0.010375434 | 0.017554723 |
| MGP | 214.1509108 | 100.4763803 | -1.091771416 | 4.81793E-25 | 2.20724E-23 |
| TFAP2A | 3.802969577 | 7.785514143 | 1.033665918 | 2.88809E-13 | 2.88295E-12 |
| ZBBX | 2.410385185 | 0.924621381 | -1.382329082 | 0.000183806 | 0.000445603 |
| H1-5 | 1.976819577 | 7.726054914 | 1.966550729 | 1.09043E-13 | 1.16177E-12 |
| NWD1 | 3.52997672 | 1.042847875 | -1.759129948 | 1.29019E-11 | 1.01175E-10 |
| SRGAP3-AS2 | 8.770751587 | 2.039644356 | -2.104382859 | 1.20517E-07 | 5.1436E-07 |
| SPAG5 | 9.500325132 | 26.67621375 | 1.489505121 | 3.3279E-50 | 1.03456E-47 |
| F11 | 1.850829365 | 0.445144422 | -2.05582651 | 2.67079E-14 | 3.12892E-13 |
| NXF3 | 1.85968254 | 0.51800923 | -1.844006656 | 0.001927915 | 0.003832443 |
| CKAP2 | 13.34348876 | 27.8719749 | 1.06267931 | 2.74232E-35 | 2.62313E-33 |
| SPOCD1 | 2.523935714 | 5.781597344 | 1.195792972 | 9.87034E-12 | 7.88585E-11 |
| MAGEB2 | 0.432032407 | 8.169745286 | 4.241079659 | 6.23746E-08 | 2.7779E-07 |
| MLLT11 | 5.452758333 | 18.47359608 | 1.760406608 | 2.26478E-17 | 4.15026E-16 |
| VEGFC | 7.240442593 | 18.51860976 | 1.354826002 | 3.57372E-09 | 1.943E-08 |
| HMMR | 5.730675265 | 18.39940226 | 1.682881846 | 1.7017E-52 | 7.05354E-50 |
| H2AC21 | 0.672771164 | 2.436235458 | 1.856465798 | 5.65436E-06 | 1.80651E-05 |
| HOXD1 | 5.044728042 | 2.320470518 | -1.120359129 | 6.69394E-08 | 2.96828E-07 |
| IGFALS | 5.426744048 | 2.307165604 | -1.233965304 | 5.53497E-13 | 5.309E-12 |
| MS4A15 | 57.85655106 | 10.09519323 | -2.518811799 | 3.24327E-14 | 3.74036E-13 |
| SGCA | 7.780691799 | 3.629725166 | -1.100038119 | 1.11516E-18 | 2.42067E-17 |
| FRG1FP | 0.540625265 | 3.950488845 | 2.869330349 | 1.99799E-07 | 8.21129E-07 |
| BCAN | 0.757575132 | 2.371632669 | 1.646419696 | 2.50121E-13 | 2.52427E-12 |
| MAT1A | 1.89203955 | 3.88027158 | 1.036215384 | 0.008548239 | 0.014762056 |
| LRRK2-DT | 17.20130013 | 5.899251527 | -1.543913785 | 3.86658E-19 | 8.81064E-18 |
| DSP | 84.34658347 | 184.7083477 | 1.130847532 | 1.13594E-19 | 2.78139E-18 |
| KLF15 | 13.96828413 | 5.402449602 | -1.370469196 | 3.21682E-25 | 1.49338E-23 |
| GRASLND | 0.628575529 | 1.479739708 | 1.235185409 | 3.4966E-18 | 7.18681E-17 |
| PFN2 | 51.93494683 | 113.3430305 | 1.125918127 | 1.67417E-19 | 3.99804E-18 |
| ABBA01000935.2 | 3.590523016 | 0.655291235 | -2.45398587 | 0.003594021 | 0.00675392 |
| MYBPHL | 11.8054709 | 4.684408035 | -1.333516937 | 1.31071E-12 | 1.19404E-11 |
| AC008870.5 | 2.731008862 | 1.260465405 | -1.115477474 | 1.83919E-06 | 6.38835E-06 |
| PRG4 | 30.58249643 | 2.50267158 | -3.611165291 | 1.74488E-12 | 1.56259E-11 |
| AC074135.1 | 1.181716138 | 3.028344821 | 1.357645962 | 4.58289E-05 | 0.000124061 |
| ANKRD44-AS1 | 6.936088757 | 1.814284529 | -1.934721633 | 4.6975E-14 | 5.28287E-13 |
| AC084375.1 | 9.815637831 | 3.295527556 | -1.574572585 | 5.70188E-17 | 9.87702E-16 |
| CIBAR2 | 10.02496164 | 2.868712948 | -1.805121196 | 2.8311E-07 | 1.13591E-06 |
| CHRNA9 | 2.810274206 | 6.749786122 | 1.264130884 | 7.10557E-06 | 2.23327E-05 |
| RAD54L | 1.996050926 | 5.918249402 | 1.568021966 | 1.15339E-44 | 1.94946E-42 |
| PRAME | 15.72808902 | 32.78762722 | 1.059808108 | 1.64741E-05 | 4.81923E-05 |
| DLX3 | 7.348721164 | 3.558084462 | -1.046392454 | 3.24387E-14 | 3.74036E-13 |
| C7 | 68.90365833 | 23.25168194 | -1.567245504 | 5.1127E-23 | 1.85818E-21 |
| SLC25A21 | 0.816794312 | 2.504852656 | 1.616681017 | 2.81731E-07 | 1.13115E-06 |
| AC010168.1 | 0.644725794 | 1.403825033 | 1.122605528 | 0.015078725 | 0.024471475 |
| AP000695.2 | 0.900569974 | 2.053607171 | 1.189249957 | 3.29691E-20 | 8.73607E-19 |
| NCAPG2 | 8.561368122 | 22.60103825 | 1.400475783 | 1.52024E-52 | 6.45507E-50 |
| RAD51 | 4.176239153 | 11.42416142 | 1.451812034 | 8.16926E-50 | 2.49506E-47 |
| MTBP | 1.69588836 | 3.50993838 | 1.049404502 | 2.08671E-33 | 1.79839E-31 |
| PTGDS | 3.275196693 | 1.596902789 | -1.036305059 | 3.23869E-17 | 5.7828E-16 |
| CXCL5 | 12.73106124 | 32.38487404 | 1.346967447 | 9.27474E-05 | 0.000238253 |
| HAS3 | 43.4744332 | 19.69012331 | -1.142695073 | 5.97167E-08 | 2.6684E-07 |
| CFAP100 | 2.290001852 | 0.647436587 | -1.822537965 | 1.59083E-09 | 9.11612E-09 |
| AC037198.1 | 2.96665291 | 5.974448008 | 1.00996928 | 0.033230223 | 0.049621572 |
| H3C7 | 0.804034788 | 2.752811687 | 1.775576093 | 1.28329E-05 | 3.83928E-05 |
| DSC3 | 1.833974603 | 3.787013612 | 1.046086946 | 2.56922E-05 | 7.26215E-05 |
| STC2 | 5.58595 | 16.98715007 | 1.604569267 | 6.16822E-20 | 1.57222E-18 |
| DLX5 | 1.904955026 | 4.384170584 | 1.202546995 | 0.019569723 | 0.030940988 |
| B3GNT5 | 7.829031746 | 17.88256494 | 1.191647882 | 1.84682E-31 | 1.44176E-29 |
| CFAP73 | 5.230038095 | 1.534083533 | -1.769444414 | 4.00498E-07 | 1.56762E-06 |
| NUF2 | 6.490873677 | 20.1874587 | 1.636974724 | 2.88749E-48 | 6.88607E-46 |
| SLC16A1 | 9.162625926 | 27.86547895 | 1.604645925 | 2.98908E-18 | 6.18016E-17 |
| SERPINB4 | 2.015929365 | 6.347755976 | 1.654801578 | 5.13419E-07 | 1.97396E-06 |
| MIR663AHG | 2.17914537 | 0.90519409 | -1.267463371 | 7.05728E-16 | 1.0483E-14 |
| DIAPH3 | 1.849760847 | 5.426564675 | 1.55270042 | 7.59245E-49 | 2.03349E-46 |
| SERPINE2 | 9.937302646 | 20.21392543 | 1.024423303 | 0.001522114 | 0.003095618 |
| CENPK | 3.207502116 | 8.10029004 | 1.336523349 | 4.11262E-42 | 5.68228E-40 |
| AC009549.1 | 0.950706217 | 2.17024409 | 1.190785814 | 0.000100833 | 0.000257205 |
| CCT8P1 | 19.7769713 | 9.300525631 | -1.088437345 | 1.56555E-10 | 1.03355E-09 |
| ACOXL | 4.497462037 | 2.072792961 | -1.117535082 | 2.38487E-22 | 8.10904E-21 |
| LINC02100 | 1.58366045 | 3.34707324 | 1.079637078 | 1.96165E-10 | 1.27475E-09 |
| GTF2IP7 | 0.582359656 | 1.606135458 | 1.463611253 | 1.55284E-22 | 5.3959E-21 |
| FAM3C2P | 19.08379696 | 40.08168792 | 1.070595022 | 3.32478E-17 | 5.93043E-16 |
| CSAG3 | 0.826148677 | 3.17683247 | 1.943115668 | 4.83918E-08 | 2.19104E-07 |
| FOLR1 | 498.9875405 | 164.2637025 | -1.60299007 | 1.80176E-25 | 8.57018E-24 |
| AIM2 | 12.36908426 | 25.77550538 | 1.05926202 | 0.00307647 | 0.005860409 |
| C1QTNF7 | 3.355894709 | 1.330198938 | -1.335055428 | 7.79333E-23 | 2.79165E-21 |
| AP003119.1 | 1.187113889 | 2.463537052 | 1.05327282 | 3.2073E-07 | 1.27508E-06 |
| SLCO4A1-AS1 | 0.886171429 | 2.55532324 | 1.527848081 | 0.000135854 | 0.000338545 |
| SCGB3A2 | 1973.862424 | 342.6854258 | -2.526064698 | 3.01571E-23 | 1.12421E-21 |
| CHAD | 8.657662037 | 3.54944741 | -1.286383046 | 9.66313E-09 | 4.87751E-08 |
| TCTEX1D1 | 2.042884524 | 0.926942895 | -1.140055288 | 7.72528E-06 | 2.40891E-05 |
| CYP4Z2P | 3.109164153 | 0.618931275 | -2.328675659 | 5.65282E-28 | 3.45298E-26 |
| NCAPH | 3.969301587 | 12.61821726 | 1.668551009 | 8.05294E-52 | 2.98284E-49 |
| VEPH1 | 9.580680026 | 4.372363811 | -1.131714612 | 1.37364E-10 | 9.15181E-10 |
| KIFC1 | 15.25275026 | 45.83621826 | 1.587418616 | 5.51594E-46 | 1.05524E-43 |
| TENM3 | 1.345957011 | 2.970694754 | 1.142168041 | 5.02445E-09 | 2.64982E-08 |
| CKS1B | 27.50003016 | 58.13770717 | 1.080040975 | 9.50186E-35 | 8.7062E-33 |
| CFAP58 | 1.678509524 | 0.675220252 | -1.313750641 | 5.67879E-07 | 2.16377E-06 |
| H2BC13 | 1.116223148 | 2.694433068 | 1.271356279 | 9.43068E-10 | 5.59573E-09 |
| PI3 | 15.85648783 | 163.8726106 | 3.369429587 | 4.53343E-05 | 0.000122798 |
| PARPBP | 1.836975661 | 4.807102523 | 1.387835061 | 5.20048E-42 | 7.12876E-40 |
| PPP1R14BP2 | 0.819849339 | 1.821001859 | 1.151301676 | 9.74549E-07 | 3.56952E-06 |
| SCGB3A1 | 3157.904878 | 298.3916574 | -3.403688609 | 1.26159E-23 | 4.86985E-22 |
| AP003119.3 | 1.49659881 | 3.339188114 | 1.157809838 | 2.21809E-18 | 4.65239E-17 |
| AHNAK2 | 11.75021098 | 27.45188652 | 1.224218635 | 5.51298E-14 | 6.13654E-13 |
| WFDC12 | 18.04302183 | 0.531007437 | -5.086565103 | 1.68615E-06 | 5.90035E-06 |
| MIR31HG | 0.725367328 | 2.057263479 | 1.503942906 | 1.30978E-10 | 8.77336E-10 |
| AC079949.2 | 0.681442989 | 2.903602191 | 2.091178941 | 1.17375E-07 | 5.01814E-07 |
| IL20RB | 6.225365476 | 17.89191839 | 1.523077641 | 1.75529E-06 | 6.11768E-06 |
| AC207130.1 | 0.331839153 | 3.148326428 | 3.246029108 | 0.000615186 | 0.001353953 |
| BLK | 2.832296429 | 1.284184197 | -1.141120116 | 1.04634E-08 | 5.25861E-08 |
| AL590004.3 | 0.48248955 | 1.611787384 | 1.740091844 | 2.09058E-06 | 7.17423E-06 |
| MACROD2 | 10.41202791 | 3.338893426 | -1.640809134 | 3.23176E-09 | 1.76535E-08 |
| AL645924.1 | 4.103362566 | 1.692623373 | -1.27754564 | 4.84844E-05 | 0.000130863 |
| ATP1B2 | 3.027764815 | 1.436765737 | -1.075428295 | 9.35071E-20 | 2.3222E-18 |
| PRR11 | 5.15663664 | 17.29713566 | 1.746030857 | 2.04035E-55 | 2.22004E-52 |
| FSD1 | 0.551699735 | 1.548731806 | 1.48913214 | 3.49283E-08 | 1.61763E-07 |
| GDF10 | 6.004122222 | 2.495381208 | -1.266693119 | 5.43483E-15 | 7.12999E-14 |
| PSAT1 | 27.68798651 | 61.85472397 | 1.159623637 | 2.30088E-17 | 4.19435E-16 |
| CACNA2D2 | 43.16229696 | 10.94856036 | -1.979030462 | 4.81971E-27 | 2.64689E-25 |
| RSPH4A | 8.134112566 | 2.829140837 | -1.523620959 | 6.30446E-06 | 1.99698E-05 |
| ELANE | 2.947100794 | 0.711898938 | -2.04955205 | 5.66413E-16 | 8.55962E-15 |
| PTHLH | 2.972835714 | 7.216512218 | 1.279462004 | 3.51438E-09 | 1.91133E-08 |
| TMEM171 | 2.432237566 | 6.444409296 | 1.405763976 | 1.20804E-11 | 9.5162E-11 |
| CFAP91 | 1.408185847 | 0.607007835 | -1.214050703 | 2.57935E-10 | 1.65392E-09 |
| LAMA1 | 0.637709788 | 2.803126295 | 2.136064816 | 3.85067E-08 | 1.77065E-07 |
| ANXA13 | 0.822607407 | 2.696482537 | 1.712802722 | 0.00094368 | 0.00200348 |
| DEPDC1B | 3.218210317 | 10.77977696 | 1.74399681 | 3.89893E-49 | 1.06057E-46 |
| F12 | 2.793103968 | 5.681363546 | 1.024367946 | 1.06166E-19 | 2.60682E-18 |
| GGH | 5.308450529 | 13.76031182 | 1.37415044 | 6.64371E-24 | 2.63463E-22 |
| KIAA1671-AS1 | 1.532219974 | 0.76548672 | -1.001174179 | 5.81805E-16 | 8.76939E-15 |
| UGT1A6 | 0.908285185 | 3.718042497 | 2.033326006 | 0.025425125 | 0.039153117 |
| PEBP4 | 96.82929788 | 18.04233851 | -2.424057291 | 6.0101E-21 | 1.74674E-19 |
| CYP2F1 | 2.194955688 | 0.638489907 | -1.781456095 | 1.34063E-06 | 4.78258E-06 |
| CR2 | 12.12528294 | 5.215353254 | -1.217181532 | 4.03118E-07 | 1.57741E-06 |
| GREB1L | 0.726337169 | 2.657899203 | 1.871575079 | 1.31422E-18 | 2.81417E-17 |
| SERPINB5 | 5.612764815 | 24.57750438 | 2.130554917 | 1.94975E-12 | 1.73534E-11 |
| CDHR4 | 5.861586905 | 1.582475697 | -1.889107954 | 4.34408E-05 | 0.000118018 |
| UCK2 | 6.277166799 | 13.41255352 | 1.095398477 | 2.42569E-21 | 7.34416E-20 |
| CTSV | 2.745573413 | 12.09957523 | 2.139777009 | 4.28601E-40 | 5.32965E-38 |
| AC105052.5 | 1.63675463 | 0.796358367 | -1.039348355 | 0.003256993 | 0.0061712 |
| FAM83A-AS1 | 3.233309788 | 8.913451262 | 1.462972406 | 2.1357E-14 | 2.54486E-13 |
| RHCG | 0.750163889 | 6.749742696 | 3.169554785 | 0.00174863 | 0.003508344 |
| FAM216B | 10.32723201 | 2.744183333 | -1.91200485 | 1.56783E-10 | 1.03466E-09 |
| HROB | 4.615842593 | 11.22593884 | 1.282170173 | 1.45161E-40 | 1.84461E-38 |
| CD274 | 8.099072487 | 19.54742636 | 1.271150069 | 1.17759E-07 | 5.03083E-07 |
| C11orf97 | 3.315332672 | 0.798871248 | -2.05311873 | 3.76719E-08 | 1.73454E-07 |
| FAM83D | 11.52504431 | 30.6994824 | 1.413442034 | 7.95634E-43 | 1.15427E-40 |
| RETN | 18.19836759 | 8.682736388 | -1.067587357 | 0.002565367 | 0.00496513 |
| RPL22P2 | 1.048283598 | 2.334908367 | 1.155336863 | 1.75408E-11 | 1.3411E-10 |
| AC010275.1 | 0.49664418 | 1.865622244 | 1.909372386 | 1.1244E-08 | 5.6249E-08 |
| SFTPD | 229.1184087 | 58.93949728 | -1.958786718 | 4.91432E-20 | 1.26934E-18 |
| HHIP | 6.324292989 | 2.259176029 | -1.48510752 | 6.22699E-07 | 2.36075E-06 |
| SCG3 | 1.618485053 | 7.371282736 | 2.187271654 | 0.01649678 | 0.026545192 |
| AZU1 | 2.801095635 | 0.685196481 | -2.031401594 | 1.25425E-10 | 8.4288E-10 |
| RANBP20P | 1.850948545 | 0.201164675 | -3.201815901 | 3.23743E-05 | 8.97364E-05 |
| AC005336.1 | 1.720967857 | 5.818608367 | 1.757453994 | 0.008547865 | 0.014762056 |
| H2AC14 | 0.728468386 | 2.211628021 | 1.602170487 | 6.35476E-11 | 4.45909E-10 |
| UBE2T | 22.85078214 | 56.49285106 | 1.305824765 | 8.06169E-44 | 1.28758E-41 |
| LINC01765 | 3.20466336 | 0.851997942 | -1.911250964 | 1.70341E-09 | 9.72285E-09 |
| AC027288.2 | 5.188706746 | 2.532587583 | -1.034762839 | 4.24834E-08 | 1.9417E-07 |
| RXRG | 2.691648545 | 0.874600133 | -1.621794573 | 6.35695E-15 | 8.24037E-14 |
| GKN2 | 55.93034484 | 8.250536653 | -2.761071358 | 6.58386E-12 | 5.43473E-11 |
| PPP2R2C | 2.011462037 | 7.210840969 | 1.841923015 | 2.88979E-06 | 9.68784E-06 |
| EFCAB1 | 4.108799603 | 1.243584462 | -1.724212472 | 8.40348E-07 | 3.11565E-06 |
| FAM83F | 1.17406164 | 3.461071846 | 1.559710735 | 1.32593E-05 | 3.94922E-05 |
| LINC01842 | 1.551133995 | 3.135712749 | 1.015470087 | 3.31063E-05 | 9.16436E-05 |
| AC012213.4 | 0.69032209 | 2.995548008 | 2.117478399 | 1.16144E-13 | 1.23365E-12 |
| AL008733.1 | 2.74659828 | 1.309104714 | -1.069065414 | 1.72289E-16 | 2.76695E-15 |
| AQP4 | 69.55143558 | 28.91461454 | -1.266281423 | 2.85449E-14 | 3.324E-13 |
| TAF7L | 0.484701852 | 1.647068327 | 1.764730905 | 9.73698E-05 | 0.000249061 |
| KRT6B | 2.617499339 | 11.97131906 | 2.193321046 | 3.20924E-06 | 1.06907E-05 |
| LINC02086 | 0.672962963 | 1.391784329 | 1.048336656 | 3.70643E-06 | 1.22045E-05 |
| MS4A1 | 13.59762817 | 6.296712284 | -1.110684372 | 5.36228E-07 | 2.05259E-06 |
| AL162511.1 | 6.717075794 | 2.405796879 | -1.481318467 | 4.94717E-22 | 1.6189E-20 |
| CCDC187 | 1.526005952 | 0.758408765 | -1.008713047 | 0.000555718 | 0.001234784 |
| APCDD1L | 0.957582143 | 2.965205644 | 1.630664009 | 8.39501E-17 | 1.41892E-15 |
| DYNLRB2 | 4.853256614 | 1.399478619 | -1.794063698 | 2.62374E-13 | 2.63418E-12 |
| TUBB2B | 12.92891614 | 34.6136081 | 1.420737999 | 0.005824058 | 0.01046239 |
| AC245884.12 | 1.642468386 | 3.303566003 | 1.008158567 | 4.30639E-09 | 2.29969E-08 |
| ALKAL2 | 1.955503307 | 0.820490438 | -1.25298155 | 4.17775E-11 | 3.01536E-10 |
| CTSG | 4.874235714 | 1.876608964 | -1.377047959 | 1.02501E-13 | 1.09475E-12 |
| CPAMD8 | 14.92438175 | 5.843634861 | -1.352733229 | 1.51592E-22 | 5.29933E-21 |
| AC097493.3 | 4.418721429 | 1.745593161 | -1.339911627 | 8.26588E-13 | 7.73244E-12 |
| GLB1L3 | 13.56434722 | 4.405543758 | -1.622427619 | 1.28259E-07 | 5.44068E-07 |
| LYPD3 | 10.20533201 | 43.39880784 | 2.088332294 | 1.28018E-15 | 1.83279E-14 |
| TUBA4B | 6.346861376 | 1.685452855 | -1.912907059 | 7.76652E-07 | 2.89958E-06 |
| GPC2 | 1.548199074 | 4.059184794 | 1.390599029 | 3.29257E-09 | 1.79576E-08 |
| HTR3A | 6.804905688 | 15.60054143 | 1.196949028 | 0.017917469 | 0.028590806 |
| TTK | 3.571933466 | 12.98763572 | 1.862361713 | 1.22529E-53 | 6.46397E-51 |
| FSCN1 | 64.46589087 | 156.865725 | 1.282922226 | 5.18898E-15 | 6.82803E-14 |
| PCLAF | 3.591773148 | 8.29157251 | 1.206949503 | 4.3049E-35 | 4.05103E-33 |
| CFAP94 | 3.448358862 | 1.625146547 | -1.085340101 | 1.5941E-10 | 1.05001E-09 |
| MST1L | 9.327384259 | 3.250072444 | -1.521000677 | 7.64933E-16 | 1.13237E-14 |
| SPDYC | 1.422122884 | 3.773895286 | 1.408008261 | 0.028722121 | 0.043586419 |
| MYO3B | 0.906255291 | 1.926323307 | 1.087860442 | 0.001906113 | 0.00379197 |
| AQP5 | 205.9786804 | 63.15185657 | -1.705597968 | 1.94847E-17 | 3.59332E-16 |
| FGB | 127.7421421 | 257.8097477 | 1.013072265 | 0.000216644 | 0.000518854 |
| TACC3 | 13.69098889 | 31.26136607 | 1.191154168 | 2.7787E-43 | 4.09953E-41 |
| AC115522.1 | 1.365869048 | 2.83979502 | 1.055967626 | 5.59747E-07 | 2.13698E-06 |
| AC116407.1 | 5.674526455 | 2.312955246 | -1.294762652 | 1.02253E-20 | 2.8945E-19 |
| AL591178.1 | 1.694712434 | 0.763610292 | -1.150132038 | 2.48065E-11 | 1.84949E-10 |
| AL445205.1 | 1.922641138 | 0.79568413 | -1.272821778 | 3.24625E-06 | 1.07964E-05 |
| ORM2 | 36.4626963 | 13.13432424 | -1.473079272 | 6.25261E-09 | 3.24253E-08 |
| FHL1 | 26.98056164 | 12.94805578 | -1.059184895 | 2.87825E-12 | 2.48426E-11 |
| H3C13 | 0.763210582 | 1.640418592 | 1.10391092 | 0.000550558 | 0.001225034 |
| AQP3 | 853.9847049 | 406.4126329 | -1.071264983 | 1.75945E-15 | 2.47418E-14 |
| ACADL | 3.296959392 | 1.508525299 | -1.127997226 | 4.30928E-11 | 3.09617E-10 |
| ADAMTS7P3 | 1.84095 | 0.395512218 | -2.218656277 | 2.36557E-14 | 2.79271E-13 |
| GCLM | 23.16346071 | 47.00929874 | 1.021095345 | 1.50737E-17 | 2.84618E-16 |
| TMEM100 | 13.61557421 | 5.307720053 | -1.359093641 | 1.40138E-11 | 1.08962E-10 |
| BLM | 2.268384259 | 5.523099004 | 1.283812938 | 4.32118E-40 | 5.33528E-38 |
| NAMPTP1 | 7.336876058 | 17.98508506 | 1.293563164 | 1.34175E-13 | 1.41395E-12 |
| WDR62 | 3.052016534 | 6.700369522 | 1.134477884 | 4.69616E-29 | 3.15658E-27 |
| ALOX15 | 12.50590569 | 4.546916932 | -1.45964899 | 6.65548E-07 | 2.51227E-06 |
| LINC00648 | 0.541117989 | 2.579219456 | 2.252919422 | 8.51719E-06 | 2.63742E-05 |
| DNAH9 | 2.237168254 | 0.528701129 | -2.081149451 | 1.15594E-07 | 4.95205E-07 |
| DRC3 | 3.636275926 | 1.661025232 | -1.130387688 | 1.92306E-14 | 2.30728E-13 |
| CYCSP6 | 0.983360317 | 6.136710757 | 2.641673543 | 2.09182E-17 | 3.84664E-16 |
| NAMPT | 59.97021693 | 140.1473813 | 1.224626691 | 8.17575E-30 | 5.88147E-28 |
| IL11 | 1.230671825 | 3.426113413 | 1.477126809 | 1.36559E-14 | 1.67774E-13 |
| ELOVL6 | 5.154128042 | 12.5013668 | 1.278285553 | 4.75591E-27 | 2.62011E-25 |
| HTR1D | 1.959857407 | 6.363387649 | 1.699046319 | 2.66645E-17 | 4.8054E-16 |
| ACE2 | 1.414540212 | 0.62795239 | -1.171606102 | 1.77502E-07 | 7.34174E-07 |
| TM4SF20 | 0.104935714 | 7.719834993 | 6.200992331 | 0.02932328 | 0.044390346 |
| CRHR2 | 1.627864418 | 0.483410425 | -1.751660054 | 0.000181934 | 0.000441619 |
| NEK2 | 8.197278571 | 27.1464075 | 1.727544356 | 4.55417E-52 | 1.8019E-49 |
| H2AC11 | 1.886054762 | 3.848972244 | 1.029101702 | 7.70062E-09 | 3.9441E-08 |
| ESPL1 | 2.849884656 | 9.491483267 | 1.73573003 | 2.07233E-49 | 5.8189E-47 |
| NCAPD2 | 30.14859365 | 66.14605983 | 1.133564515 | 3.76022E-40 | 4.70947E-38 |
| ATOH8 | 18.01120913 | 7.499428088 | -1.264042552 | 2.29246E-16 | 3.63143E-15 |
| IL33 | 35.72061257 | 16.91137623 | -1.078762752 | 6.12231E-19 | 1.37173E-17 |
| GCLC | 18.11437566 | 61.64937351 | 1.766951152 | 1.1315E-17 | 2.18385E-16 |
| MYEOV | 9.254241402 | 29.41017131 | 1.66812855 | 1.14054E-12 | 1.04558E-11 |
| AC239859.5 | 0.485575661 | 2.153590106 | 2.148975671 | 1.29217E-06 | 4.62487E-06 |
| PITX1 | 8.996924339 | 22.25730246 | 1.306774956 | 7.99959E-13 | 7.49542E-12 |
| AL079303.1 | 0.931138228 | 1.959977092 | 1.073769535 | 9.90295E-07 | 3.62186E-06 |
| CENPI | 2.206675 | 6.628719854 | 1.586856118 | 5.91026E-51 | 1.94135E-48 |
| PLA2G4F | 12.59142513 | 4.323046481 | -1.542321327 | 8.99398E-17 | 1.50554E-15 |
| CLU | 318.6944929 | 129.2912724 | -1.301549197 | 6.79905E-19 | 1.50975E-17 |
| SYNGR4 | 0.763452116 | 1.865051195 | 1.288605653 | 1.09496E-16 | 1.81027E-15 |
| NKAIN1 | 0.412774339 | 2.100603984 | 2.347379015 | 4.21751E-11 | 3.03902E-10 |
| S100A2 | 48.33848439 | 160.9431458 | 1.735306992 | 7.54985E-06 | 2.36055E-05 |
| AP000357.2 | 6.345189021 | 3.020410226 | -1.070918635 | 2.55698E-09 | 1.41631E-08 |
| PIFO | 21.2455672 | 8.27868413 | -1.359688481 | 2.83162E-19 | 6.56401E-18 |
| HIGD1B | 9.363426852 | 4.615788645 | -1.020459463 | 4.49133E-19 | 1.02075E-17 |
| AL355916.1 | 1.020610847 | 4.97000498 | 2.283814418 | 8.1055E-07 | 3.01643E-06 |
| AC120498.4 | 1.733086111 | 4.743649602 | 1.452654108 | 0.000199375 | 0.000480337 |
| DNAI1 | 4.105885847 | 1.235549336 | -1.732540898 | 8.94862E-06 | 2.75869E-05 |
| PPP1R3G | 1.397704497 | 3.052297543 | 1.126836227 | 8.45223E-17 | 1.42582E-15 |
| AP000251.1 | 1.000579365 | 2.098191899 | 1.068311027 | 1.22062E-06 | 4.39228E-06 |
| LINC02038 | 7.168816005 | 3.371402058 | -1.088386178 | 3.96258E-14 | 4.49411E-13 |
| FOXM1 | 8.894518915 | 34.6924085 | 1.963631522 | 4.23872E-54 | 2.54455E-51 |
| DNAJC6 | 2.367230423 | 4.84125332 | 1.032180443 | 1.04548E-11 | 8.32604E-11 |
| DSG3 | 0.248924074 | 4.237973705 | 4.089596968 | 0.008248164 | 0.014297835 |
| ELN | 71.99373624 | 34.03601056 | -1.080809446 | 2.54838E-20 | 6.82533E-19 |
| HOXA10 | 1.258542196 | 3.047268061 | 1.275762829 | 4.10958E-05 | 0.000112085 |
| PRDM16 | 7.898032011 | 3.437240106 | -1.200242581 | 6.79527E-18 | 1.34431E-16 |
| KRT83 | 0.774786376 | 1.915759296 | 1.306045816 | 0.020297906 | 0.031999117 |
| SPHK1 | 7.490395238 | 17.252516 | 1.20369302 | 1.39122E-25 | 6.69052E-24 |
| LY6G6C | 0.334038624 | 2.212126428 | 2.727347007 | 0.001489381 | 0.003035784 |
| FANCI | 4.382928836 | 10.92318074 | 1.317425859 | 2.07158E-47 | 4.6236E-45 |
| TRIP13 | 10.95869405 | 35.04737311 | 1.677230433 | 9.63222E-47 | 1.97279E-44 |
| MCM2 | 11.86785397 | 31.29579695 | 1.398909835 | 1.68844E-36 | 1.70896E-34 |
| SPC24 | 5.983340476 | 15.12333898 | 1.337753632 | 2.42496E-43 | 3.67097E-41 |
| CDK5R2 | 0.729112434 | 3.753938645 | 2.364191861 | 1.44169E-09 | 8.31626E-09 |
| CRYBG2 | 2.35598955 | 5.963001195 | 1.339705486 | 1.04971E-13 | 1.12044E-12 |
| LINC02057 | 0.689759392 | 1.501108699 | 1.121863349 | 1.74099E-06 | 6.07759E-06 |
| AL031058.1 | 3.848555556 | 8.765897078 | 1.187584667 | 1.90698E-16 | 3.05416E-15 |
| LINC02122 | 2.52588955 | 1.146366733 | -1.139722907 | 8.13323E-07 | 3.02546E-06 |
| MAGEA1 | 2.413073016 | 11.61051799 | 2.266488863 | 1.07379E-06 | 3.90425E-06 |
| MFAP4 | 189.848737 | 82.84946255 | -1.196286158 | 8.61531E-20 | 2.14263E-18 |
| DLX6 | 0.544051323 | 1.620073904 | 1.574244969 | 0.031867909 | 0.047818345 |
| COL22A1 | 1.651531349 | 3.830588513 | 1.213761703 | 0.004151322 | 0.007686702 |
| GPR37 | 2.995404497 | 7.490896082 | 1.322387472 | 1.14307E-12 | 1.04735E-11 |
| AL513318.1 | 0.720275661 | 2.31247012 | 1.682813663 | 0.012672279 | 0.020964716 |
| FAM183A | 18.53655423 | 7.03670571 | -1.397401003 | 5.99168E-08 | 2.67665E-07 |
| CPM | 150.3437179 | 74.71565226 | -1.008782174 | 4.64382E-08 | 2.11082E-07 |
| CDT1 | 12.19238545 | 30.86601016 | 1.34003859 | 9.44118E-38 | 1.02726E-35 |
| FBXL13 | 1.004727513 | 2.119330212 | 1.076804102 | 1.12641E-05 | 3.40979E-05 |
| ZWINT | 26.63112817 | 67.84405584 | 1.349108873 | 2.58853E-47 | 5.56342E-45 |
| SLC7A5 | 55.37108704 | 131.3012499 | 1.245675899 | 2.22923E-17 | 4.08943E-16 |
| C22orf15 | 2.556428704 | 0.726854582 | -1.814391126 | 4.06354E-07 | 1.589E-06 |
| POLE2 | 4.559545635 | 10.49653499 | 1.202951189 | 1.0383E-37 | 1.12272E-35 |
| SLC35G2 | 3.773765741 | 7.597277888 | 1.009477723 | 6.7921E-13 | 6.43328E-12 |
| CHIT1 | 49.6956705 | 22.7077162 | -1.129937553 | 4.17099E-08 | 1.90735E-07 |
| SH3PXD2A-AS1 | 1.044458466 | 2.099497278 | 1.007288795 | 7.34678E-12 | 6.0047E-11 |
| GAPDH | 1120.429221 | 2405.168927 | 1.102086711 | 9.09121E-46 | 1.68371E-43 |
| GPR1 | 0.669094974 | 1.354075299 | 1.017025057 | 3.46499E-06 | 1.14746E-05 |
| ENPP1 | 1.603804365 | 3.394025697 | 1.081499318 | 5.09642E-12 | 4.26351E-11 |
| AL139023.1 | 0.62727037 | 2.227325963 | 1.828153385 | 1.82791E-09 | 1.03756E-08 |
| PICSAR | 0.839583333 | 3.23740259 | 1.947091351 | 0.006879847 | 0.012154582 |
| E2F8 | 3.737274206 | 9.61416089 | 1.363174528 | 7.46755E-40 | 9.15511E-38 |
| SCML2 | 0.978924206 | 2.512707902 | 1.359973902 | 3.65476E-08 | 1.68679E-07 |
| AC004990.1 | 0.638138095 | 1.46265332 | 1.196647294 | 3.72702E-06 | 1.22677E-05 |
| CYP24A1 | 53.69445675 | 117.139223 | 1.125379169 | 3.79747E-08 | 1.7471E-07 |
| PTPRN | 0.426491931 | 4.038021448 | 3.243058221 | 3.39776E-21 | 1.01114E-19 |
| SAXO2 | 4.66224246 | 1.671080212 | -1.480243049 | 2.13505E-10 | 1.38175E-09 |
| MIR9-1HG | 0.307917196 | 1.781095352 | 2.532150411 | 2.6804E-17 | 4.82555E-16 |
| LRRC55 | 1.866816138 | 0.763679548 | -1.28954055 | 1.76036E-06 | 6.1329E-06 |
| BTNL9 | 5.81829246 | 2.039660624 | -1.512266692 | 5.04528E-19 | 1.13922E-17 |
| UCA1 | 1.31251746 | 3.549279216 | 1.435189459 | 0.002468892 | 0.004799122 |
| PRIM1 | 3.639776984 | 7.662737118 | 1.074009756 | 1.83937E-29 | 1.27069E-27 |
| NHLRC4 | 3.371044709 | 1.495479482 | -1.172587645 | 6.6848E-10 | 4.05208E-09 |
| CNTN6 | 1.423165476 | 0.641062483 | -1.150566532 | 3.62319E-09 | 1.96622E-08 |
| MME | 3.884164683 | 7.968863546 | 1.036769623 | 0.001413365 | 0.002894398 |
| STIL | 3.60028254 | 9.167818659 | 1.348468378 | 5.92416E-48 | 1.37512E-45 |
| CTSH | 211.1213112 | 73.83882304 | -1.515620754 | 9.70008E-28 | 5.78318E-26 |
| OGN | 9.529651587 | 3.14232988 | -1.600588827 | 2.01348E-12 | 1.78931E-11 |
| CDCA4 | 14.14678386 | 30.48083566 | 1.107428349 | 5.68029E-38 | 6.21938E-36 |
| SLC46A2 | 10.59181759 | 3.112516733 | -1.766796684 | 1.9139E-14 | 2.29788E-13 |
| CABCOCO1 | 6.391119577 | 2.241919323 | -1.511334318 | 8.28052E-14 | 8.99303E-13 |
| AP003469.2 | 1.988292196 | 4.437954648 | 1.158365136 | 6.52518E-14 | 7.18596E-13 |
| GRAMD1B | 2.201149603 | 5.214323373 | 1.244222854 | 0.001174595 | 0.002447167 |
| BRCA1 | 3.581674868 | 9.396407039 | 1.39147483 | 1.03074E-44 | 1.77665E-42 |
| NPTX2 | 6.354131217 | 13.45140923 | 1.081990536 | 0.000739677 | 0.001603216 |
| GATA6-AS1 | 1.63288955 | 0.597307835 | -1.450880657 | 7.17455E-11 | 4.99807E-10 |
| NKAIN4 | 1.178505159 | 3.061437649 | 1.377251227 | 0.001188563 | 0.00247183 |
| JCHAIN | 1299.328341 | 647.6432804 | -1.004494741 | 1.7526E-12 | 1.56789E-11 |
| CDKN3 | 8.176378175 | 26.76608566 | 1.71087234 | 9.08259E-54 | 4.94121E-51 |
| CEP55 | 11.30972526 | 38.52665093 | 1.768292896 | 1.58766E-56 | 3.07107E-53 |
| CCT6A | 140.242845 | 280.6853278 | 1.001026484 | 2.36192E-37 | 2.44754E-35 |
| GALNT13 | 1.222811376 | 2.552925232 | 1.061949408 | 0.004298465 | 0.007936379 |
| TPI1P1 | 4.82507381 | 10.00391833 | 1.051942268 | 2.85513E-17 | 5.11369E-16 |
| CASC8 | 0.800268915 | 1.804054382 | 1.172686052 | 6.21891E-05 | 0.000165063 |
| LINC00540 | 0.68740172 | 1.4608751 | 1.087607472 | 0.000544817 | 0.001214121 |
| XRCC2 | 2.906119841 | 7.191413546 | 1.307181178 | 1.02324E-38 | 1.18757E-36 |
| STOML3 | 4.744867196 | 1.282536388 | -1.88736795 | 3.73562E-07 | 1.46868E-06 |
| ZBTB16 | 9.44293082 | 3.121681208 | -1.596911487 | 2.16146E-14 | 2.57029E-13 |
| DSCC1 | 5.893393386 | 15.52607928 | 1.397523085 | 1.05785E-44 | 1.8055E-42 |
| TPSB2 | 45.87899484 | 22.37118699 | -1.036191978 | 4.63247E-11 | 3.31743E-10 |
| UBE2C | 47.51070582 | 165.0796924 | 1.79683811 | 8.34252E-44 | 1.32032E-41 |
| HES2 | 1.569887963 | 4.124639841 | 1.393606547 | 0.000887942 | 0.001896945 |
| PAX7 | 8.72105582 | 3.548698938 | -1.297212621 | 3.80879E-09 | 2.05731E-08 |
| SFTPA1 | 5070.545323 | 1971.176939 | -1.36308363 | 4.54448E-12 | 3.81828E-11 |
| DNER | 3.861419048 | 9.551566135 | 1.30660618 | 0.008342427 | 0.014442474 |
| TIMP4 | 1.541049735 | 3.336418991 | 1.114387051 | 0.004777811 | 0.008733402 |
| NDC80 | 5.556844974 | 18.86187815 | 1.763135441 | 9.86191E-53 | 4.4022E-50 |
| PKIB | 8.245435317 | 21.90900943 | 1.409856689 | 3.81159E-13 | 3.74681E-12 |
| CDCA8 | 14.41299418 | 43.85132556 | 1.605250379 | 1.94151E-53 | 9.94112E-51 |
| GNPNAT1 | 12.77536601 | 26.66194622 | 1.061417471 | 1.77163E-35 | 1.71346E-33 |
| LINC02577 | 1.301859524 | 4.679122112 | 1.845664095 | 6.00021E-17 | 1.03321E-15 |
| LINC01224 | 0.786544312 | 2.281465737 | 1.536361036 | 1.11207E-07 | 4.77672E-07 |
| VWA3A | 3.580890873 | 0.955054515 | -1.906663563 | 2.08012E-10 | 1.34771E-09 |
| H2BC3 | 0.59376164 | 2.266963811 | 1.932805564 | 0.000164781 | 0.000403413 |
| SLC1A7 | 30.0109541 | 12.41193293 | -1.273761379 | 1.86725E-11 | 1.41766E-10 |
| RNVU1-31 | 3.382679233 | 7.649298473 | 1.177161064 | 0.00319688 | 0.006067207 |
| DNAH6 | 1.497398413 | 0.622031541 | -1.26739849 | 1.12095E-09 | 6.59055E-09 |
| CDH2 | 1.806327646 | 4.779782205 | 1.403885278 | 0.000226887 | 0.000541154 |
| C5orf49 | 13.44657817 | 4.960475896 | -1.438688648 | 7.01281E-17 | 1.19692E-15 |
| TWIST1 | 3.135415608 | 6.50446328 | 1.052773328 | 9.33784E-12 | 7.49827E-11 |
| TMEM132D | 1.885212698 | 0.535611155 | -1.815469391 | 5.35281E-14 | 5.96207E-13 |
| STAC2 | 3.672683333 | 1.381839509 | -1.410244443 | 1.40811E-05 | 4.16689E-05 |
| FBN2 | 0.772621429 | 3.740238645 | 2.275296728 | 9.68334E-05 | 0.000247944 |
| CIT | 24.03179034 | 10.3036921 | -1.221782744 | 2.44268E-06 | 8.29424E-06 |
| GABRA3 | 1.108890608 | 3.803118459 | 1.778065825 | 4.23478E-06 | 1.38033E-05 |
| H2BC10 | 0.900852778 | 2.319500465 | 1.364450878 | 0.008190499 | 0.014210524 |
| ABCA8 | 4.035440212 | 1.471582205 | -1.455357925 | 1.44092E-17 | 2.7296E-16 |
| CFAP65 | 1.826197751 | 0.533383267 | -1.775598526 | 6.00466E-09 | 3.12512E-08 |
| DRC7 | 1.726496561 | 0.632454714 | -1.448813373 | 2.10314E-05 | 6.03388E-05 |
| H4C2 | 1.376625265 | 2.976388845 | 1.112427124 | 0.001391906 | 0.002857174 |
| GPRIN1 | 5.230503439 | 11.4230674 | 1.126928386 | 1.55767E-23 | 5.97301E-22 |
| AC027288.3 | 2.812209127 | 1.227647942 | -1.195806991 | 4.0547E-06 | 1.32562E-05 |
| AL590666.2 | 3.975503704 | 9.595020186 | 1.271148177 | 1.36002E-11 | 1.06125E-10 |
| TNXB | 7.819161772 | 2.878985126 | -1.441453621 | 2.85321E-12 | 2.46508E-11 |
| AC236972.3 | 3.137930291 | 0.60050259 | -2.385570931 | 2.90811E-25 | 1.36831E-23 |
| AMH | 1.052198545 | 2.269432935 | 1.108924895 | 1.80606E-07 | 7.45946E-07 |
| AL096854.1 | 0.947262037 | 2.196407902 | 1.213310534 | 0.006630618 | 0.01175124 |
| CCNB2 | 12.1922373 | 39.19863831 | 1.684840651 | 2.51051E-56 | 3.97323E-53 |
| PLPPR1 | 8.131514418 | 3.86396587 | -1.073441715 | 2.31242E-11 | 1.72999E-10 |
| RSPO3 | 1.565738492 | 10.54792862 | 2.752044533 | 1.29653E-05 | 3.87293E-05 |
| TPSP2 | 1.529445503 | 3.105047809 | 1.02160678 | 0.000889828 | 0.001900656 |
| CLEC3B | 20.34842302 | 8.320390837 | -1.290193788 | 1.52976E-22 | 5.3263E-21 |
| MIR3135B | 1.635346296 | 0.503118991 | -1.700624617 | 4.91E-11 | 3.50321E-10 |
| C1QL1 | 0.721347487 | 2.468566268 | 1.774907069 | 1.90586E-13 | 1.96095E-12 |
| TFRC | 46.11068267 | 94.63797384 | 1.037318161 | 4.964E-18 | 1.0037E-16 |
| RPL32P33 | 0.688102646 | 1.413233201 | 1.038303852 | 1.27776E-08 | 6.32575E-08 |
| SUSD2 | 209.115416 | 46.92939608 | -2.155735621 | 1.88931E-27 | 1.10372E-25 |
| E2F1 | 19.7042295 | 40.27561833 | 1.031401402 | 1.27549E-28 | 8.31646E-27 |
| AC025154.2 | 8.676787698 | 3.85715166 | -1.169625157 | 4.8939E-17 | 8.50769E-16 |
| AQP7 | 4.793985053 | 2.120857769 | -1.176577538 | 1.77257E-15 | 2.48861E-14 |
| CHIAP2 | 3.188471296 | 0.546994489 | -2.543266691 | 8.12117E-12 | 6.58507E-11 |
| GINS4 | 2.122960979 | 5.401826892 | 1.347369554 | 4.15724E-36 | 4.11212E-34 |
| FHOD3 | 1.887372487 | 5.03448413 | 1.415464779 | 1.78204E-08 | 8.6057E-08 |
| SMC1B | 1.14427209 | 2.848710691 | 1.315878969 | 2.14983E-07 | 8.78966E-07 |
| RHOBTB2 | 63.42106878 | 30.55862151 | -1.053382725 | 2.79572E-17 | 5.0176E-16 |
| DCDC2B | 2.061333069 | 0.785149934 | -1.392537548 | 2.1496E-07 | 8.78966E-07 |
| FCER1A | 26.78450886 | 9.588759031 | -1.481982822 | 1.2975E-16 | 2.11105E-15 |
| CCDC114 | 5.541344841 | 2.423045219 | -1.193414821 | 1.59839E-05 | 4.68614E-05 |
| C9orf24 | 15.54246085 | 3.715644223 | -2.064530671 | 4.86557E-08 | 2.20071E-07 |
| CD1B | 3.844769709 | 1.900844356 | -1.016256781 | 2.51844E-10 | 1.61665E-09 |
| TMEM158 | 4.975078571 | 10.81947224 | 1.120838913 | 1.33775E-17 | 2.55641E-16 |
| RFC3 | 11.69197698 | 23.67346886 | 1.017752224 | 2.22969E-32 | 1.81386E-30 |
| SERPINB7 | 0.751317989 | 3.458812218 | 2.202781139 | 4.11484E-07 | 1.60789E-06 |
| HIF1A-AS3 | 1.969259127 | 4.456867862 | 1.178377226 | 2.7679E-14 | 3.23184E-13 |
| GGT6 | 21.22405476 | 8.485308831 | -1.322661229 | 1.56019E-17 | 2.92372E-16 |
| AFF3 | 2.431301587 | 1.038496414 | -1.227232627 | 1.80357E-15 | 2.52805E-14 |
| AL022098.1 | 1.498667593 | 0.650007703 | -1.205151707 | 1.95696E-15 | 2.72986E-14 |
| CFAP161 | 1.63904418 | 0.591479947 | -1.47045358 | 0.000100569 | 0.000256603 |
| GJB3 | 8.492752513 | 27.78406089 | 1.709953363 | 3.69671E-10 | 2.31247E-09 |
| MELK | 7.90366164 | 28.36788745 | 1.843665627 | 2.97351E-53 | 1.43794E-50 |
| PGC | 4639.25278 | 465.4758987 | -3.317114081 | 1.10438E-19 | 2.70791E-18 |
| IL1R2 | 4.165653307 | 8.759975963 | 1.072384137 | 4.21648E-15 | 5.64218E-14 |
| C2CD4A | 11.04788704 | 23.05738187 | 1.061458233 | 0.000124683 | 0.000312722 |
| HMGA1P3 | 0.745249471 | 1.584780279 | 1.088487481 | 9.89988E-17 | 1.65242E-15 |
| LTF | 138.5652624 | 68.72661142 | -1.011624893 | 8.87114E-05 | 0.000228458 |
| INSL4 | 3.404632143 | 14.07427404 | 2.047489675 | 0.001180787 | 0.002458302 |
| AL008729.2 | 32.8294705 | 16.3069409 | -1.009505316 | 9.07544E-23 | 3.23097E-21 |
| AREG | 81.84166111 | 168.6680908 | 1.043279733 | 0.002828742 | 0.005431297 |
| APOH | 39.23354537 | 15.65867776 | -1.325125317 | 1.51543E-07 | 6.34947E-07 |
| CAMK2N2 | 1.983033995 | 5.527331408 | 1.478873707 | 6.77993E-17 | 1.16059E-15 |
| NEIL3 | 2.221123413 | 9.496012417 | 2.096032266 | 5.81243E-47 | 1.21914E-44 |
| LINC01518 | 0.666220238 | 1.339542165 | 1.007668908 | 5.13927E-07 | 1.97548E-06 |
| PAX8 | 1.640522884 | 4.130794223 | 1.332263476 | 0.030445986 | 0.045934174 |
| KLK8 | 0.976848148 | 2.875670916 | 1.557692371 | 0.000514275 | 0.001151513 |
| AL138760.1 | 1.622473677 | 3.27169429 | 1.011842875 | 0.000529598 | 0.001182477 |
| DIO3OS | 1.936060979 | 0.779819256 | -1.311912708 | 6.62608E-13 | 6.28286E-12 |
| AC112722.1 | 1.635298148 | 0.804956906 | -1.022570238 | 6.03226E-12 | 5.01268E-11 |
| LRRC18 | 1.820382804 | 0.656929548 | -1.470431299 | 1.01983E-07 | 4.40878E-07 |
| DNAI2 | 3.742817328 | 0.85653579 | -2.127539202 | 1.32125E-10 | 8.84341E-10 |
| TK1 | 66.72124431 | 162.8902732 | 1.287682358 | 2.16461E-38 | 2.44699E-36 |
| HLF | 15.81756296 | 6.233443625 | -1.343426043 | 1.81273E-23 | 6.87535E-22 |
| MELTF | 4.568765212 | 16.52458074 | 1.854737459 | 2.18827E-21 | 6.64844E-20 |
| FAM81B | 7.63518664 | 3.276851262 | -1.220353234 | 5.64277E-07 | 2.15333E-06 |
| ITLN1 | 153.3390415 | 3.345373572 | -5.518413934 | 4.88927E-08 | 2.21084E-07 |
| YBX2 | 2.768863757 | 6.046326228 | 1.126764755 | 1.36921E-07 | 5.78136E-07 |
| TMPRSS2 | 81.90601124 | 39.09639788 | -1.066933646 | 3.36879E-23 | 1.24781E-21 |
| AL691432.4 | 2.155585714 | 1.013325963 | -1.088981601 | 2.41734E-14 | 2.85118E-13 |
| CENPM | 10.9180041 | 22.26832131 | 1.028283661 | 3.39123E-29 | 2.32433E-27 |
| EXO1 | 3.606028042 | 12.96683772 | 1.846344165 | 4.24682E-55 | 3.69665E-52 |
| LY6K | 2.571430423 | 6.078205378 | 1.241074305 | 3.79131E-07 | 1.4889E-06 |
| DKK1 | 12.03215754 | 36.48699462 | 1.600486961 | 2.86049E-05 | 8.02032E-05 |
| RFC4 | 13.58066958 | 30.77200113 | 1.180063655 | 1.04426E-40 | 1.35667E-38 |
| RN7SL8P | 2.522540608 | 1.16006919 | -1.12066664 | 7.96354E-12 | 6.47232E-11 |
| SLC5A9 | 2.293503968 | 0.952582271 | -1.2676378 | 8.31925E-14 | 9.02366E-13 |
| C13orf46 | 2.554058598 | 1.243575232 | -1.038297837 | 1.75258E-12 | 1.56789E-11 |
| KLK6 | 3.07395582 | 13.6582587 | 2.15160523 | 1.22613E-09 | 7.15309E-09 |
| KLHL2P1 | 0.874443386 | 2.142719256 | 1.293005949 | 8.4537E-24 | 3.31465E-22 |
| CLSPN | 2.037136111 | 6.273360226 | 1.62269603 | 2.12372E-46 | 4.20134E-44 |
| EREG | 7.577706349 | 33.37416056 | 2.138898411 | 1.3264E-07 | 5.61284E-07 |
| B3GAT1-DT | 4.452933598 | 1.917095618 | -1.215833803 | 2.5358E-19 | 5.91767E-18 |
| GAP43 | 0.737060847 | 2.248417596 | 1.609054383 | 5.0059E-08 | 2.26064E-07 |
| KIF18B | 4.613547751 | 15.60226786 | 1.757807253 | 2.49791E-47 | 5.43577E-45 |
| AC104031.1 | 7.961037169 | 3.371408699 | -1.23960487 | 8.18189E-06 | 2.53901E-05 |
| AP000695.1 | 1.373284392 | 3.061437981 | 1.156579035 | 1.49641E-19 | 3.59327E-18 |
| SLC34A3 | 0.649254894 | 1.432323971 | 1.141500957 | 0.000136472 | 0.000339843 |
| AC156455.1 | 0.910342857 | 2.231250199 | 1.293370391 | 5.19757E-05 | 0.000139593 |
| ELOVL4 | 1.323139286 | 4.219668127 | 1.673164596 | 5.35372E-07 | 2.05022E-06 |
| PADI3 | 1.96035119 | 8.556277955 | 2.125871218 | 2.16044E-06 | 7.39794E-06 |
| ATAD2 | 15.17696389 | 33.6383415 | 1.148223365 | 1.05489E-37 | 1.13361E-35 |
| SLC7A11 | 9.711656746 | 20.821533 | 1.100286956 | 1.974E-12 | 1.75602E-11 |
| SHE | 14.65750886 | 7.070980611 | -1.051657721 | 2.36864E-19 | 5.55736E-18 |
| ADH1B | 23.55549749 | 5.956126228 | -1.983617567 | 3.66637E-27 | 2.05234E-25 |
| ALPG | 6.070018783 | 2.685170385 | -1.176687345 | 0.000143772 | 0.000356086 |
| TEDC2 | 5.121829233 | 10.25100212 | 1.001033895 | 2.47905E-27 | 1.41501E-25 |
| CLGN | 3.750601455 | 13.47201109 | 1.844771358 | 5.85997E-08 | 2.62185E-07 |
| PRDM16-DT | 3.146025926 | 0.992269788 | -1.664726227 | 1.02806E-22 | 3.63769E-21 |
| FAM222A-AS1 | 0.672361111 | 1.712235657 | 1.348573088 | 2.761E-08 | 1.29628E-07 |
| CENPN | 4.174945767 | 8.957438313 | 1.101328746 | 1.12044E-42 | 1.61205E-40 |
| SCTR | 34.26913214 | 9.795662351 | -1.806694705 | 8.02958E-25 | 3.56599E-23 |
| LINC02315 | 0.92334709 | 3.293988313 | 1.834890467 | 3.68507E-08 | 1.69943E-07 |
| CAPN6 | 14.12170291 | 6.70752324 | -1.074062018 | 9.60953E-09 | 4.85186E-08 |
| CLTRN | 10.41776429 | 4.938314343 | -1.076955122 | 2.63341E-06 | 8.89675E-06 |
| UPK1B | 4.729350132 | 32.38840126 | 2.775763397 | 0.008563403 | 0.014786778 |
| BPIFB1 | 303.0179927 | 111.3316497 | -1.444539677 | 0.000368225 | 0.000845145 |
| LHFPL3 | 2.519454762 | 1.145341434 | -1.137333812 | 2.48554E-15 | 3.43965E-14 |
| LINC01612 | 1.979830291 | 0.816977689 | -1.277008184 | 8.34986E-12 | 6.76106E-11 |
| KNSTRN | 9.722935053 | 20.33253101 | 1.064326025 | 1.16005E-37 | 1.23898E-35 |
| CFAP57 | 3.964425132 | 1.301666467 | -1.606751854 | 6.86627E-16 | 1.02254E-14 |
| C15orf48 | 26.14444431 | 64.00728911 | 1.291731801 | 2.50514E-14 | 2.94476E-13 |
| KLK1 | 1.460272222 | 3.597435126 | 1.300731331 | 0.010149019 | 0.017215655 |
| AC011298.1 | 0.589225529 | 1.961721647 | 1.735228505 | 0.00772046 | 0.013475586 |
| RPL39L | 30.7936955 | 73.05186202 | 1.246286033 | 3.74412E-22 | 1.24155E-20 |
| TREML3P | 0.981158333 | 4.616927822 | 2.234375305 | 8.86169E-12 | 7.13567E-11 |
| MPP2 | 1.423414021 | 2.870261222 | 1.01182669 | 3.24631E-05 | 8.9949E-05 |
| SLC6A4 | 2.101116931 | 0.878970319 | -1.257270099 | 3.93674E-06 | 1.2902E-05 |
| MAGEA12 | 3.166243915 | 15.89802324 | 2.328003087 | 2.83818E-07 | 1.13821E-06 |
| NMRAL2P | 3.62816336 | 12.0075583 | 1.726631493 | 5.22645E-06 | 1.67626E-05 |
| TOP2A | 33.12223095 | 105.1222512 | 1.666196322 | 2.82384E-49 | 7.80322E-47 |
| BTG2 | 250.7048229 | 124.7816559 | -1.00658389 | 5.60422E-23 | 2.02415E-21 |
| CPB2 | 5.328054101 | 1.499696082 | -1.828938567 | 1.05486E-10 | 7.1651E-10 |
| SELENBP1 | 288.570922 | 105.2848262 | -1.454628405 | 5.17355E-29 | 3.46409E-27 |
| WDR76 | 5.840959127 | 12.99598227 | 1.153788486 | 5.63533E-38 | 6.2092E-36 |
| TPPP3 | 92.57160397 | 29.92657922 | -1.62914234 | 7.01282E-17 | 1.19692E-15 |
| IGF2BP3 | 2.03047672 | 8.668058898 | 2.09389047 | 7.98636E-24 | 3.13848E-22 |
| BRCA2 | 1.559626058 | 3.173879814 | 1.025047334 | 1.40379E-31 | 1.10582E-29 |
| TRPM8 | 2.194844444 | 7.644072709 | 1.800222806 | 0.00053643 | 0.001196549 |
| CYP4F3 | 2.998044709 | 13.10992663 | 2.128565808 | 0.000108207 | 0.000274602 |
| MYOZ1 | 6.963228968 | 3.45023506 | -1.01306181 | 4.09445E-15 | 5.49578E-14 |
| AC245041.1 | 1.375027381 | 4.769925365 | 1.794506345 | 0.006128021 | 0.01094743 |
| SPINK13 | 6.508910979 | 2.277084396 | -1.51522842 | 0.014321396 | 0.023353426 |
| CNGA4 | 2.216575397 | 0.748428619 | -1.566395805 | 1.20912E-10 | 8.15873E-10 |
| ADGRD1 | 11.4373418 | 5.14443672 | -1.152666762 | 4.70395E-18 | 9.5333E-17 |
| FHL5 | 4.707249339 | 2.311458499 | -1.026080813 | 3.62263E-15 | 4.90789E-14 |
| SFRP5 | 3.545952646 | 1.563314143 | -1.181565557 | 8.24924E-05 | 0.000213866 |
| AKR1C7P | 0.662945635 | 2.08751089 | 1.654821252 | 2.07127E-10 | 1.34247E-09 |
| CDC20 | 27.76147447 | 95.17759973 | 1.777537877 | 3.04713E-51 | 1.02014E-48 |
| C1orf87 | 2.141582407 | 0.525743426 | -2.026246381 | 5.43359E-09 | 2.85174E-08 |
| VIPR1 | 8.613225529 | 4.138453519 | -1.057461853 | 5.60425E-21 | 1.6425E-19 |
| H3C3 | 1.055825794 | 3.765833931 | 1.834597564 | 1.11244E-08 | 5.57467E-08 |
| CDCP1 | 27.22317447 | 56.29574163 | 1.048190489 | 1.00256E-16 | 1.6702E-15 |
| LGI3 | 6.156091138 | 1.372747875 | -2.164947912 | 7.40341E-09 | 3.80083E-08 |
| E2F7 | 1.120590608 | 4.185036653 | 1.900980952 | 1.09154E-35 | 1.0616E-33 |
| SPOCK1 | 4.791162169 | 13.68979216 | 1.514652993 | 2.703E-14 | 3.16452E-13 |
| AL365181.3 | 6.933111905 | 20.14061421 | 1.538532729 | 1.36362E-13 | 1.4344E-12 |
| CCDC33 | 1.808361508 | 0.759756109 | -1.251074839 | 0.014912144 | 0.024239545 |
| KIF14 | 2.158940476 | 7.687447078 | 1.832181106 | 8.74341E-54 | 4.91013E-51 |
| DUSP5P1 | 0.524951323 | 1.556264807 | 1.567832007 | 1.26784E-18 | 2.72156E-17 |
| CCNB1 | 29.07252262 | 88.37704369 | 1.604015413 | 5.7234E-55 | 4.7447E-52 |
| EGLN3 | 18.18564749 | 43.76387404 | 1.26694016 | 3.48546E-17 | 6.16649E-16 |
| AKAP12 | 11.45334722 | 39.35298353 | 1.780703732 | 2.26773E-05 | 6.4709E-05 |
| H2AC16 | 0.642819577 | 2.003282935 | 1.639880424 | 3.02296E-10 | 1.91998E-09 |
| C11orf16 | 4.741913492 | 1.934227025 | -1.293712206 | 1.05214E-13 | 1.12167E-12 |
| GREM1 | 9.094998677 | 19.39610166 | 1.092621387 | 1.13583E-15 | 1.63689E-14 |
| ZNF474 | 2.175518651 | 0.929951195 | -1.226132476 | 0.001230182 | 0.002551991 |
| CDHR3 | 7.402438889 | 2.775699668 | -1.415149199 | 1.89097E-11 | 1.43504E-10 |
| C6 | 4.040658466 | 1.181002324 | -1.77457861 | 7.7951E-17 | 1.32395E-15 |
| AC007906.2 | 21.76967368 | 5.929107105 | -1.876433019 | 7.33683E-10 | 4.42267E-09 |
| DRC1 | 5.776434392 | 2.393108699 | -1.27129331 | 0.005136734 | 0.009324859 |
| ASF1B | 19.66701336 | 48.79691428 | 1.311012034 | 2.29488E-45 | 4.16162E-43 |
| CDC45 | 6.199035714 | 19.0622838 | 1.620605254 | 1.39645E-46 | 2.79434E-44 |
| AC016877.3 | 1.175035979 | 2.418655644 | 1.041500448 | 9.68018E-15 | 1.21501E-13 |
| TSNAXIP1 | 1.79968254 | 0.811671846 | -1.148773963 | 1.302E-05 | 3.8879E-05 |
| AC012511.1 | 5.344766931 | 2.289554582 | -1.223060079 | 6.7148E-20 | 1.69446E-18 |
| DTHD1 | 2.942678175 | 1.050838048 | -1.485589429 | 1.88436E-05 | 5.45385E-05 |
| AP003390.1 | 1.178449206 | 2.424710159 | 1.040922727 | 3.93907E-09 | 2.1211E-08 |
| VGF | 2.348123413 | 13.71868367 | 2.546561918 | 1.52278E-13 | 1.59124E-12 |
| LAMC2 | 77.50580595 | 241.5898512 | 1.640183559 | 3.22174E-17 | 5.75845E-16 |
| FAM72B | 0.550051984 | 1.987042297 | 1.852982707 | 3.51937E-55 | 3.22467E-52 |
| RNU1-38P | 2.067764947 | 0.374551461 | -2.464836343 | 6.87569E-15 | 8.8661E-14 |

Supplementary Table 2 Legend: Risk related differential expressed genes were generated from prognostic signature risk groups，a total of 1098 genes were obtained.
